# The difluoromethylene (CF_2_) group in aliphatic chains: Synthesis and conformational preference of palmitic acids and nonadecane containing CF_2_ groups

**DOI:** 10.3762/bjoc.10.4

**Published:** 2014-01-06

**Authors:** Yi Wang, Ricardo Callejo, Alexandra M Z Slawin, David O’Hagan

**Affiliations:** 1 EaStCHEM School of Chemistry, University of St. Andrews, St. Andrews, Fife KY16 9ST, UK

**Keywords:** difluoromethylene, fatty acids, fluorination, organic fluorine chemistry, organo-fluorine, palmitic acid

## Abstract

The syntheses of palmitic acids and a nonadecane are reported with CF_2_ groups located 1,3 or 1,4 to each other along the aliphatic chain. Specifically 8,8,10,10- and 8,8,11,11-tetrafluorohexadecanoic acids (**6b** and **6c**) are prepared as well as the singly modified analogue 8,8-difluorohexadecanoic acid (**6a**). Also 8,8,11,11-tetrafluorononadecane (**27**) is prepared as a pure hydrocarbon containing a 1,4-di-CF_2_ motif. The modified palmitic acids are characterized by differential scanning calorimetry (DSC) to determine melting points and phase behaviour relative to palmitic acid (62.5 °C). It emerges that **6c**, with the CF_2_ groups placed 1,4- to each other, has a significantly higher melting point (89.9 °C) when compared to the other analogues and palmitic acid itself. It is a crystalline compound and the structure reveals an extended *anti-*zig-zag chain. Similarly 8,8,11,11-tetrafluorononadecane (**27**) adopts an extended *anti*-zig-zag structure. This is rationalized by dipolar relaxation between the two CF_2_ groups placed 1,4 to each other in the extended *anti-*zig-zag chain and suggests a design modification for long chain aliphatics which can introduce conformational stability.

## Introduction

**Figure 1:**
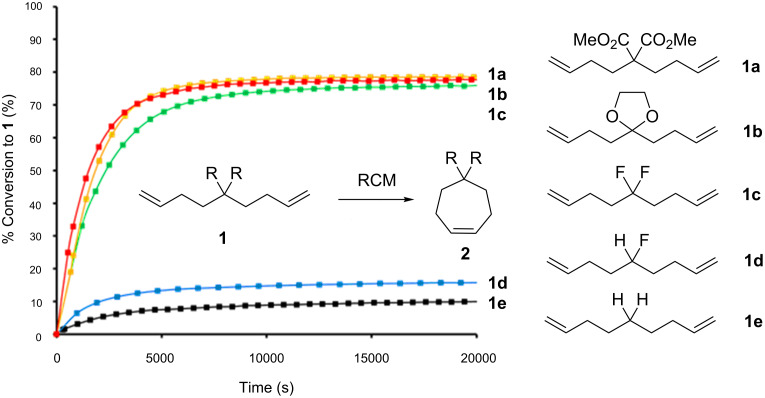
The CF_2_ group in **1c** accelerates RCM reactions relative to CHF (**1d**) and CH_2_ (**1e**) and with a similar rate to classical or Thorpe–Ingold substituents such as the ketal **1a** and dicarboxylate ester **1b** [8].

The selective replacement of hydrogen by fluorine is widely practised in bio-organic and medicinal chemistry [1–4]. It is generally perceived that fluorine exerts only a moderate steric influence relative to hydrogen in organic compounds, but that the electronegativity of fluorine can have significant electronic influences [5]. The difluoromethylene (CF_2_) functionality has received considerably less attention as a functional group for modifying the properties of organic molecules, relative to –F and –CF_3_ groups. However we have recently become interested in the CF_2_ group, and in particular have noticed that the replacement of the two hydrogen atoms of a methylene by two fluorine atoms leads to widening of the C–CF_2_–C angle (~118°) and a narrowing of the F–C–F angle (104°) relative to tetrahederal geometry [6–7]. This deviation of classical sp^3^, towards sp^2^ hybridisation, imparts certain properties to the CF_2_ group in that it can accommodate angle strain. For example CF_2_ compounds display an apparent Thorpe–Ingold effect relative to CH_2_ in ring closing metathesis reactions (RCM) to cycloheptene [8]. Comparison of the rates of reaction with different substituents at the C-5 position of the diene precursors **1a–d**, revealed that the CF_2_ substituent in **1c** was as effective as the dicarboxylate **1a** or ketal **1b** in promoting RCM (Figure 1). This is attributed to C–CF_2_–C angle widening, which absorbs angle strain in the resultant cycloheptene **2c**.

**Figure 2:**
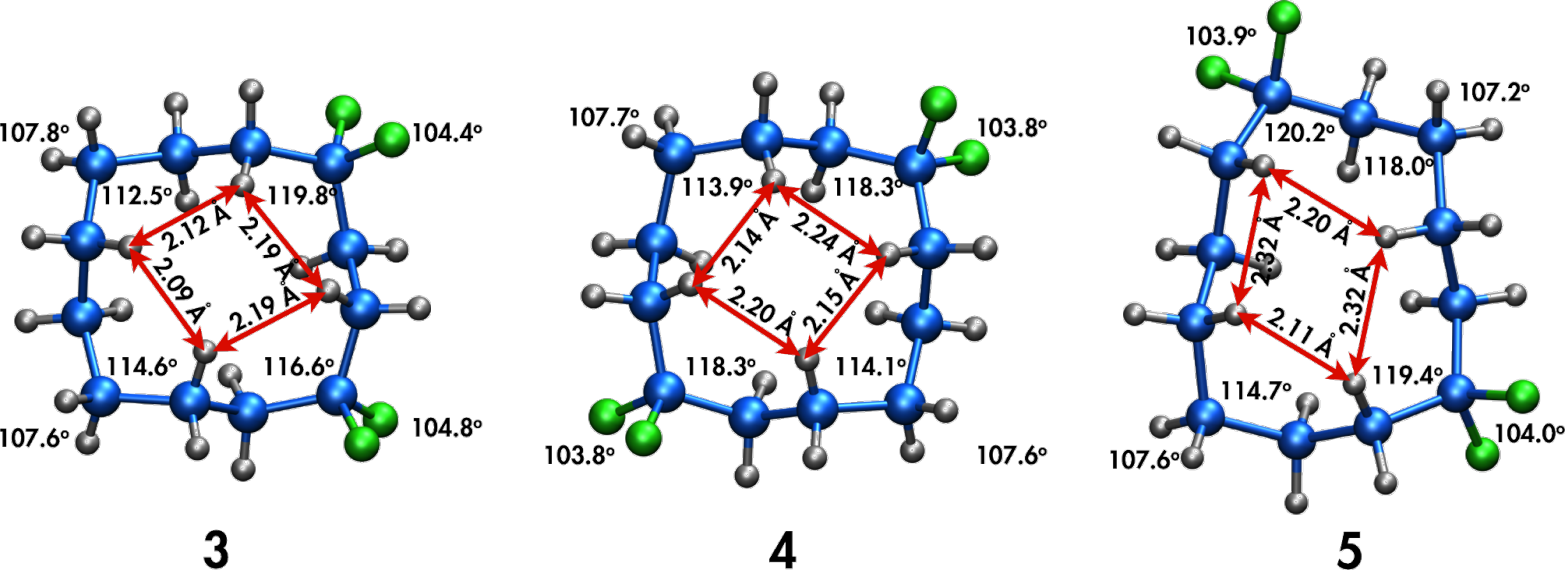
X-ray structures of a) 1,1,4,4- (**3**) b) 1,1,7,7- (**4**) and c) 1,1,6,6- (**5**) tetrafluorocyclododecanes. The CF_2_ groups locate at the corners, even for **5** which gives rise to a distorted ring conformation [6–7].

In another study we have prepared cyclododecanes **3**–**5** with regiospecific placement of two CF_2_ groups around the ring [6] (Figure 2). X-ray structures reveal that the CF_2_ groups only ever occupy corner locations. This is a result of several factors including C–CF_2_–C angle widening, which relaxes 1,4-torsional strain across corner positions, lengthening the contact distance between those H(1)···H(4) interactions relative to those with CH_2_ at the corner. Also if the CF_2_ locates at an edge this would require that a C–F bond project into the middle of the ring. The larger steric influence of the fluorine, projecting into the tightly packed arrangement of endo orientated hydrogen atoms, raises the energy of such conformations. For cyclododecane, placing the CF_2_ groups 1,4 (**3**) or 1,7 (**4**) to each other, stabilizes the [3.3.3.3] square like conformation of the ring. However if the CF_2_ groups are placed 1,6 to each other as in **5**, this introduces considerable distortion of the ring conformation as shown in Figure 2, because the CF_2_ avoids an edge location, which would place a fluorine atom *endo* and unfavourably into the centre of the ring.

**Figure 3:**
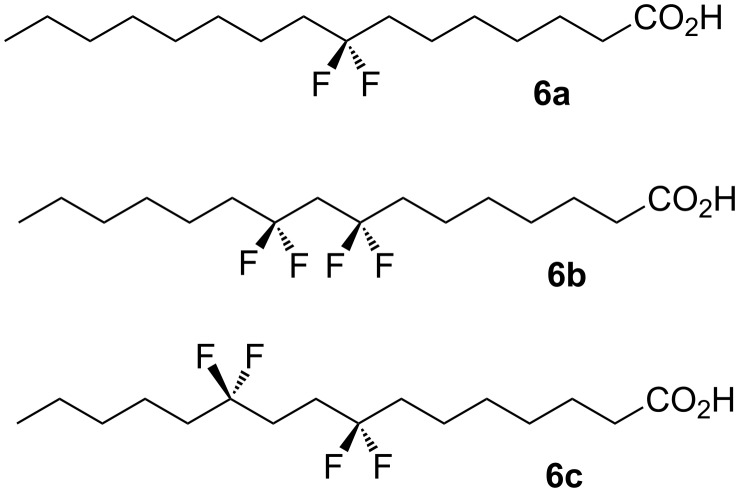
Synthesis targets: Palmitic acid analogues **6a–c**.

As part of an on-going interest in the behaviour and influence of the CF_2_ group we have now explored the effect of locating two CF_2_ groups along an extended aliphatic chain. Long chain fatty acids present tractable model systems as they are solid materials and their physical properties are well described [9]. In this study we selected the three palmitic acid analogues **6a–c** shown in Figure 3, as targets for synthesis and comparative analysis.

Palmitic acid **6a** containing a single CF_2_ group at C-8 was prepared as a control compound. The location for CF_2_ substitution in the middle of the aliphatic chain was selected as it is sufficiently remote from the carboxylic acid head group to have any electronic influence. Two additional analogues **6b** and **6c** were prepared, each with two CF_2_ groups, located 1,3 and 1,4 from each other respectively. These targets were designed to explore the significance on properties and chain stability of co-locating the CF_2_ groups at different distances from each other.

## Results and Discussion

### Synthesis of the palmitic acids **6a–c**

**Scheme 1:**
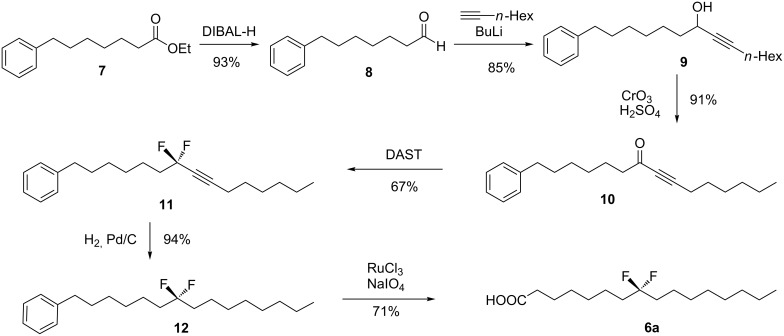
Synthesis route to 8,8-difluorohexadecanoic acid (**6a**).

As a general strategy palmitic acids **6a–c** were prepared by aryl oxidation of long chain pentadecabenzenes [10–11]. The introduction of the CF_2_ groups was carried out by treatment of the appropriate precursor ketone with diethylaminosulfur trifluoride (DAST) [12–13]. The synthesis of palmitic acid **6a** is illustrated in Scheme 1. At the outset aldehyde **8** was condensed with the acetylide of 1-octyne to afford propargylic alcohol **9**, an alcohol which was readily oxidized to ketone **10**. Treatment with DAST afforded difluoromethyleneacetylene **11** in good yield. The fluorination of propargylic ketones, to generate difluoromethyleneacetylenes, is methodology developed by Grée et al. [14–18] and it proved to be very reliable in our hands. An efficient hydrogenation generated the C-8 substituted difluoromethylenepentadecabenzene **12**. Finally biphasic ruthenium tetroxide-catalyzed aryl oxidation gave the palmitic acid **6a** in 24% overall yield as illustrated in Scheme 1 [10–11].

**Scheme 2:**
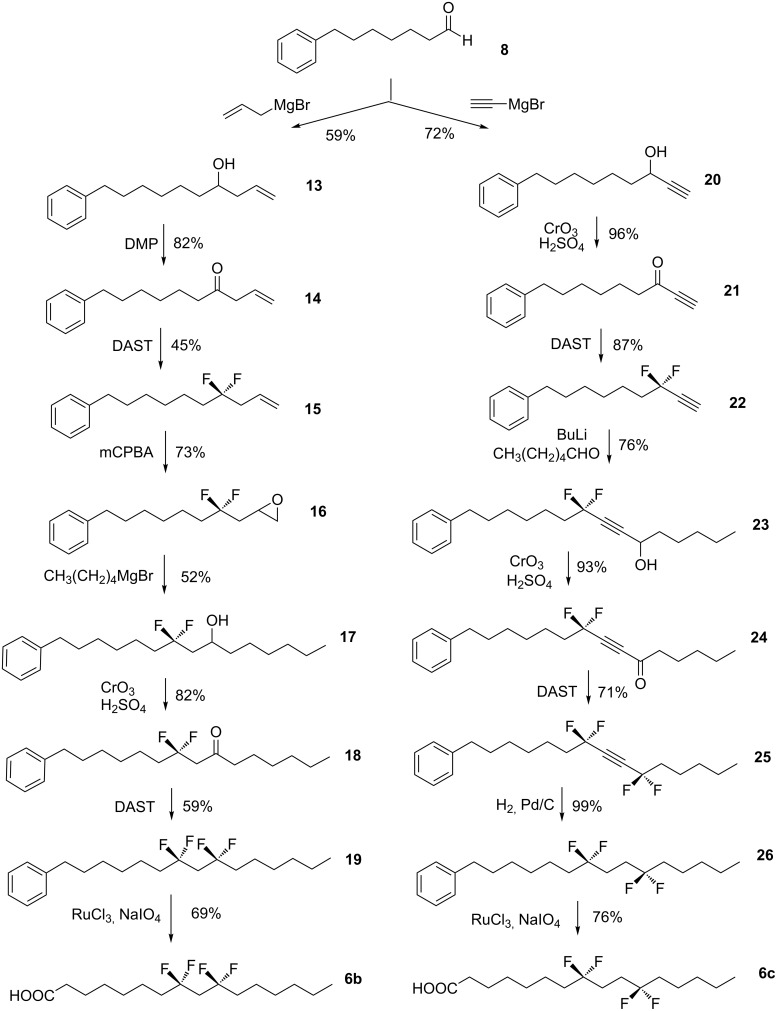
The synthesis of palmitic acid analogues **6b** and **6c**.

For palmitic acid **6b**, it was required to introduce the CF_2_ groups 1,3 to each other. This was achieved by sequential preparation of appropriate precursor ketones as illustrated in Scheme 2. For the first CF_2_ group ketone **14** was treated with DAST. Conversion to the CF_2_ group occurred in modest (45%) yield. Generally aliphatic ketones are less efficiently converted to CF_2_ groups with DAST in comparison to propargylic ketones. Progression of the resultant CF_2_ containing olefin **15** by epoxidation, chain extension and then oxidation, to ketone **18**, generated the second fluorination substrate of the synthesis. DAST treatment gave pentadecabenzene **19**, which was again oxidised by RuO_3_ to the corresponding palmitic acid **6b**.

Palmitic acid **6c** was prepared again relying on the methodology developed by Grée et al. [14–17] for introduction of the CF_2_ groups. Thus treatment of ketone **21** with DAST resulted in an efficient conversion to difluoromethyleneacetylene **22**. This terminal acetylene is amenable to acetylide formation on treatment with BuLi [19–20] and condensation with hexaldehyde gave propargylic alcohol **23**. The lithium methylenedifluoroacetylide (RCF_2_C≡CLi) reaction to form a C–C bond, provides a particularly useful synthon to access this 1,4-di-CF_2_ motif. Oxidation and then treatment of the resultant ketone **24**, with DAST generated the tetrafluoroacetylene **25**. Complete hydrogenation of the triple bond proved efficient and the resultant tetrafluoropentadecabenzene **26** was readily oxidized to palmitic acid **6c** as illustrated in Scheme 2. This completed the syntheses of the palmitic acid analogues **6a**–**c**.

**Figure 4:**
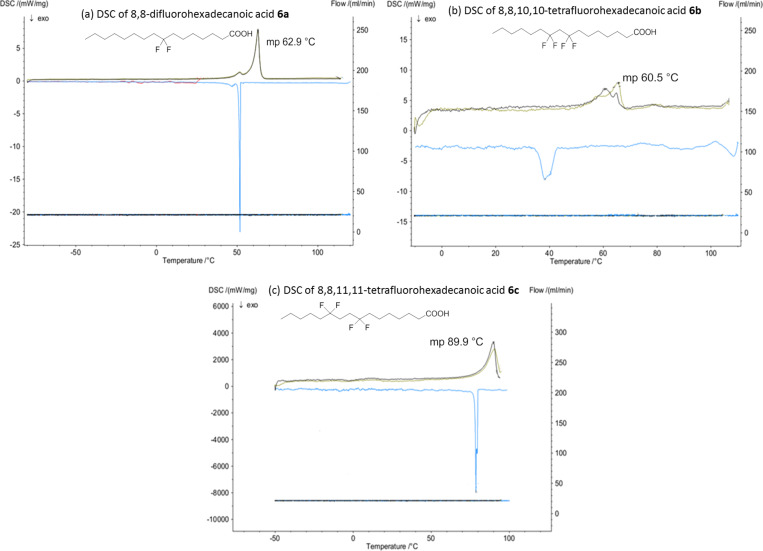
DSC traces for the three palmitic acid analogues **6a–c**.

Differential scanning calorimetry (DSC) data was then measured for all three of the palmitic acid samples **6a–c** over a temperature range of −150 to 400 °C. In this way accurate melting point values were obtained. The melting point of C-8 difluorinated palmitic acid **6a** (62.9 °C) was very similar to the natural palmitic acid (62.5 °C), Thus a single CF_2_ substitution, certainly at this location, has very little influence on the melting point. For palmitic acid **6b**, with the two CF_2_-groups placed 1,3 to each other, the melting point (69 °C) is also similar to palmitic acid, but the phase behaviour is more complex as evidenced by the broad DSC profiles. This palmitic acid **6b** was amorphous in nature and was not a crystalline solid, unlike the other two analogues **6a** and **6c** which formed crystals (Figure 4).

The tetrafluorinated palmitic acid **6c**, with the CF_2_ groups located 1,4 from each other displays a sharp and significantly higher melting point (89.9 °C) than the other two palmitic acids **6a** and **6b**.

**Figure 5:**
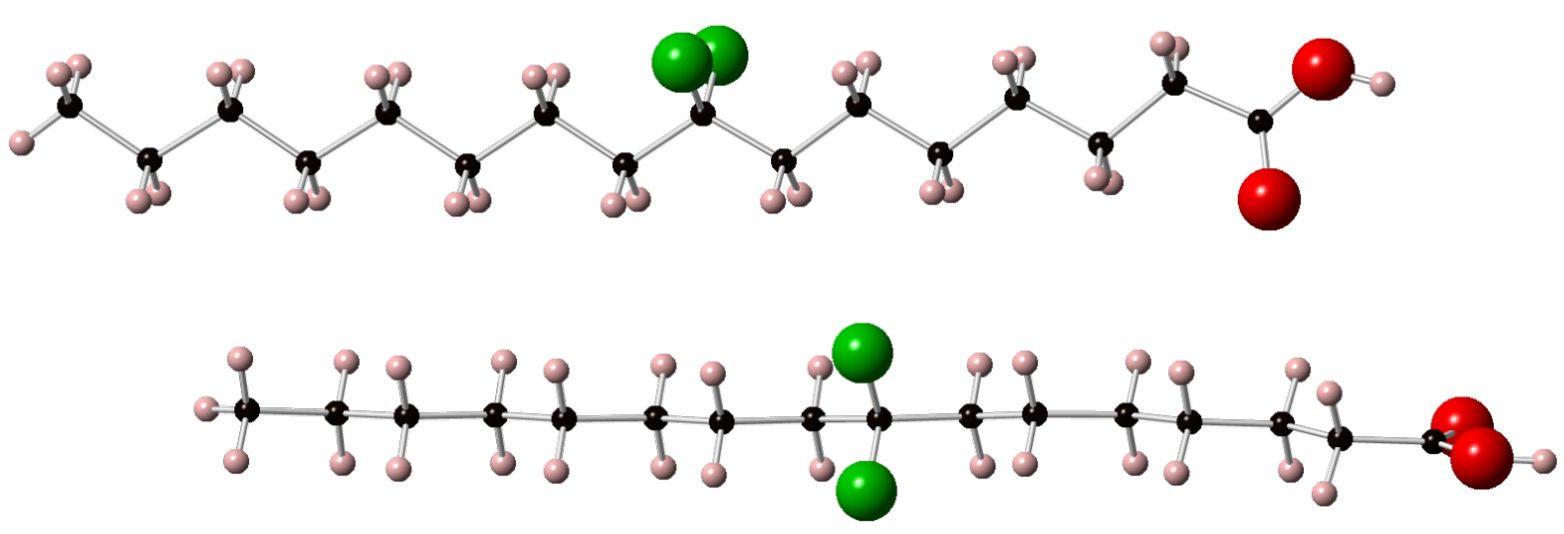
The X-ray crystal structures of 8,8-difluorohexadecanoic acid (**6a**).

**Figure 6:**
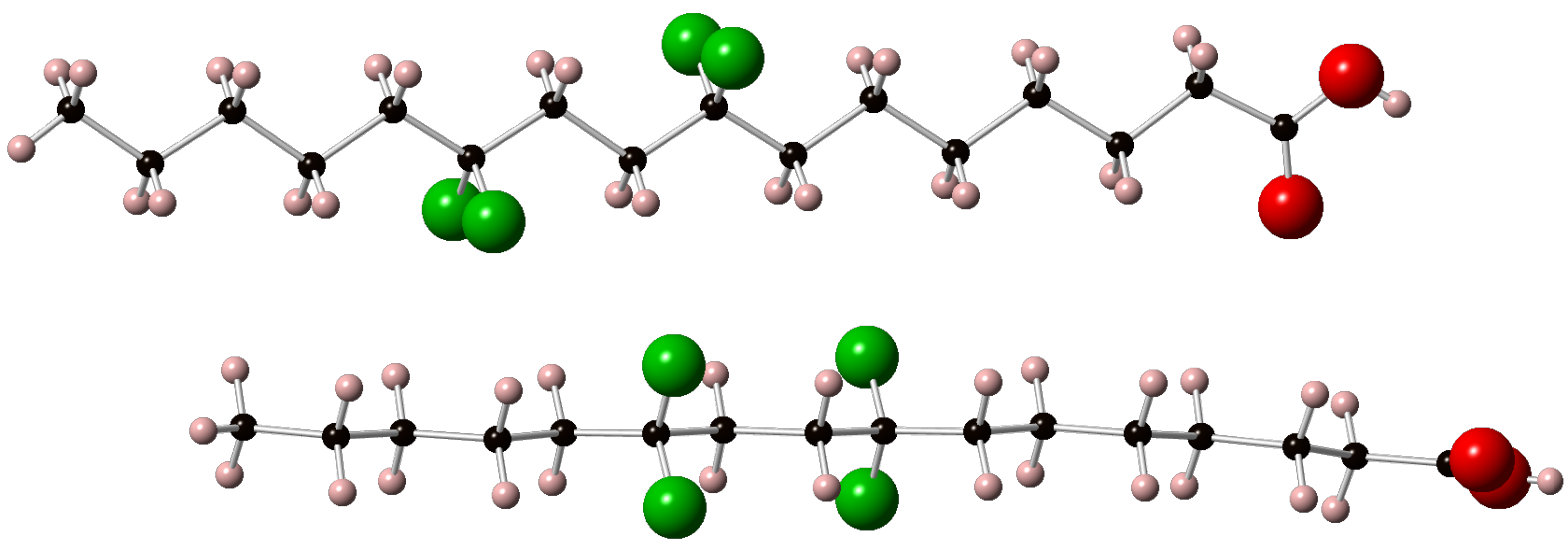
The X-ray structure of 8,8,11,11-tetrafluorohexadecanoic acid (**6c**).

Palmitic acids **6a** and **6c** were crystalline solids and single crystal X-ray diffraction data were obtained for these compounds. As described above analogue **6b** was amorphous in nature and despite considerable effort a single crystal could not be obtained for **6b**. The resultant structures for **6a** and **6c** are shown in Figure 5 and Figure 6 respectively. In each case two molecules as they appear within the unit cell are presented in the image, allowing a view from above and to the side of the extended chain. The closest CF···HC contacts are 2.88 Å in **6a** and 2.85 Å in **6c**, much longer than any meaningful organic fluorine hydrogen bond [21]. The C–CF_2_–C angle in **6a** (Figure 5) is 117° and as expected, wider than the other C–CH_2_–C angles which are typically ~112.5°. For **6c** (Figure 6) the C–CF_2_–C angles are 115.6° (at C-8) and 116.3° (at C-11) also consistently wider that the aliphatic C–CH_2_–C angles. The significantly higher melting point and good crystallinity of **6c** can be attributed to the relative orientation of the two CF_2_ groups. They are pointing perfectly *anti-*parallel to each other such that their dipoles cancel out in the extended *anti-*zig-zag chain conformation. We are currently exploring if this is a special situation whereby CF_2_ groups positioned 1,4 from each other can add conformational stability to aliphatic chains in other systems.

**Scheme 3:**
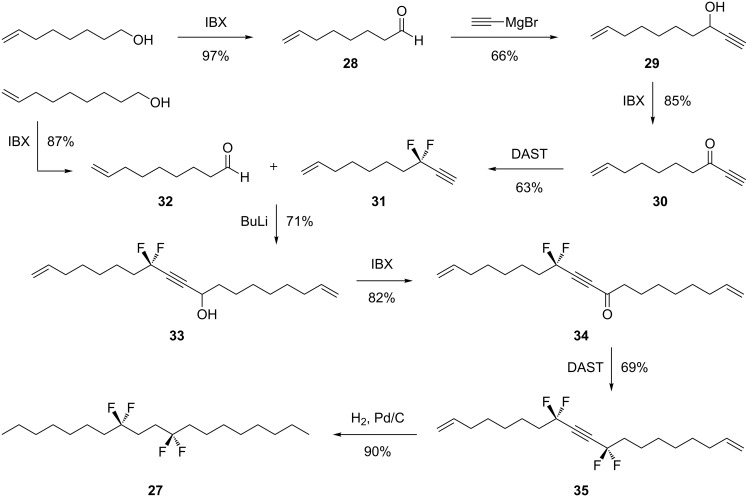
Synthesis route to the tetrafluorinated alkane **27**.

**Figure 7:**
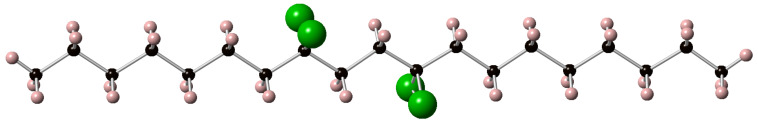
The X-ray structure of 8,8,11,11-tetrafluorononadecane (**27**).

It occurred to us that the interactions of the carboxylate groups in palmitic acid **6c**, may be dictating overall stability and conformation of the alkyl chain in the solid state. Thus it appeared appropriate to prepare a true hydrocarbon chain to further investigate the conformational preference of the 1,4-di-CF_2_ motif. Accordingly we selected to prepare tetrafluorononadecane **27**. This is a long chain hydrocarbon with the 1,4-di-CF_2_ motif placed centrally. The synthetic route to **27** is illustrated in Scheme 3. The strategy for incorporating the two CF_2_ groups followed that used for the preparation of palmitic acid **6c**. In this case propargylic ketone **30** was treated with DAST to generate difluoroacetylene **31**. The resultant acetylene could then be deprotonated for conjugation to aldehyde **32**. Oxidation and then fluorination of ketone **34** with DAST, introduced the second CF_2_ group and generated tetrafluoroacetylene **35**. Finally hydrogenation of the central acetylene group gave the saturated tetrafluorononadecane **27**. This compound proved to be a crystalline solid (mp 35–37 °C) with a melting point very similar to nonadecane (32–35 °C). A suitable crystal was subject to X-ray structure analysis and the resultant structure is shown in Figure 7. It is clear that the alkyl chain of **27** is extended in a similar conformation to that found in palmitic acid **6c** and we conclude that this is the preferred conformation of this motif in a hydrocarbon chain.

## Conclusion

In conclusion, we have synthesised three palmitic acid analogues **6a**–**c** carrying regiospecifically located CF_2_ groups. The tetrafluorononadecane **27** was also prepared as an example of a true hydrocarbon. Relatively efficient synthesis protocols were devised for placing the CF_2_ groups 1,3 and 1,4 to each other. The CF_2_ groups of **6b**, **6c** and **27** were introduced sequentially from appropriate precursor ketones, using DAST. In particular, the methodology of Grée et al., enabled the efficient introduction of CF_2_ groups from propargylic ketones in the syntheses of **6a, 6c** and **27**. A useful C–C bond forming reaction involved a lithium methylenedifluoroacetylide (RCF_2_C≡CLi) condensation with an aldehyde, offers an efficient strategy for the preparation of the 1,4-di-CF_2_ motif after suitable functional group manipulations.

**Figure 8:**
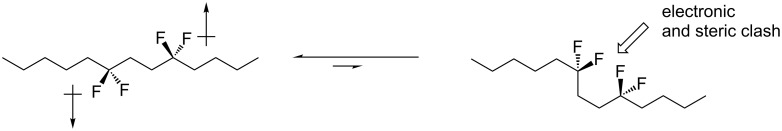
Conformational interconversion of 1,4-di-CF_2_ motif.

The non-crystalline nature of **6b** presumably arises due to chain disorder from linear 1,3-repulsions between the fluorines, so the preferred conformation of this motif could not be determined in this study. The melting point of palmitic acid **6c** (89.9 °C) was notable in that it was significantly higher than that of the two other analogues **6a** and **6b**, and also of palmitic acid itself. The solid state structure of **6c** and **27** show that the 1,4-di-CF_2_ motif prefers an *anti-*zig-zag conformation. We attribute this preference to intramolecular dipole–dipole relaxation which is maximised in the extended *anti-*zig-zag chain conformation (Figure 8). Also repulsive through space 1,4-F···F interactions will be disfavoured if the chain undergoes *gauche* conformational disorder. These contributing factors suggest that the 1,4-di-CF_2_ motif (R–CF_2_CH_2_CH_2_CF_2_–R) will be useful for adding conformational stability to aliphatic chains.

## Supporting Information

File 1 Experimental part.
