# Supplementary material for: The difluoromethylene (CF2) group in aliphatic chains: Synthesis and conformational preference of palmitic acids and nonadecane containing CF2 groups
Source: Beilstein J Org Chem. 2014 Jan 6;10:18–25. doi: 10.3762/bjoc.10.4 (PMC3896246; doi:10.3762/bjoc.10.4)

Supporting Information

for

The difluoromethylene (CF_2) group in aliphatic chains: Synthesis and conformational preference of palmitic acids and nonadecane containing CF_2 groups

Yi Wang, Ricardo Callejo, Alexandra M. Z. Slawin and David O'Hagan*

Address: EaStCHEM School of Chemistry, University of St. Andrews, St. Andrews, Fife KY16 9ST, UK.

Email: David O'Hagan - do1@st-andrews.ac.uk

*Corresponding author

Contents **Page**

General information S1

Preparation and characterisation S2

NMR spectra S13

General information

All commercially availble reagents were purchased from Acros, Alfa Aesar, Fisher Scientific, Fluorochem or Sigma-Aldrich and used without further purification unless otherwise stated. All glassware was flame-dried and allowed to cool down under high vacuum. Dry solvents DCM, Et_2O, hexane, THF were obtained from the MBraun SPS-800 Solvent Purification System, by passing the solvent through two drying columns under an argon atmosphere.

Thin layer chromatography (TLC) was performed using Merck TLC silica gel 60 F_{254} aluminium-backed plates. Compounds were visualised by either UV light (254 nm) or by the use of potassium permanganate stain or molybdenum-based stain. Column chromatography was performed using Merck silica gel 60 (40–63 μm).

NMR spectra were acquired on either Bruker Avance 300 (^{1}H at 300 MHz, ^{13}C at 75 MHz, ^{19}F at 282 MHz), Bruker Avance II 400 spectrometer (^{1}H at 400 MHz, ^{13}C at 100 MHz, ^{19}F at 376 MHz) or Bruker Avance III 500 spectrometer (^{1}H at 500 MHz, ^{13}C at 125 MHz, ^{19}F at 470.6 MHz). Chemical shifts (δ) are reported in parts per million (ppm) and are quoted relative to the residual peak of $CDCl_3$ or CD_2Cl_2. Coupling constants (*J*) are given in Hertz (Hz). ^{13}C NMR and ^{19}F NMR spectra were recorded with ^{1}H decoupling. Signal splitting patterns are described as: s – singlet, t – triplet, tt – triplet of triplets, q – quartet, quin – quintet, abq – AB quartet, m – multiplet.

Mass spectrometric data was acquired by electron impact ionisation (EI), electrospray ionisation (ESI) or chemical ionisation (CI). At the University of St Andrews LRMS and HRMS examination was carried out by Mrs. C. Horsburgh on a Waters Micromass LCT (ESI) or GCT (CI) spectrometer. At the National Mass Spectrometry Service Centre, Swansea, the LRMS assessment was performed on Thermofisher DSQ-II spectrometer (CI) and HRMS was performed on Finnigan MAT 95 XP (EI). Values are reported as a ratio of mass to charge (*m*/*z*).

Preparation and characterisation

7-Phenylheptanal (8) To a solution of methyl 7-phenylheptanoate **7** (5.5 g, 25 mmol) in CH_2Cl_2 (50 mL) was added DIBAL-H (27 mL, 1 M in THF, 27 mmol) dropwise at −78 °C. After 30 min, the reaction was quenched with saturated Rochelle's salt solution (100 mL). The organic layer was separated and washed with 1 M HCl (30 mL) and brine (30 mL). Then it was dried over $MgSO_4$ and concentrated to yield the aldehyde (4.4 g, 93%) as a colourless oil; ^{1}H NMR (400 MHz, $CDCl_3$) δ_H 9.77 (t, *J* = 1.8 Hz, 1H, CHO), 7.31-7.27 (m, 3H, ArH), 7.21-7.18 (m, 2H, ArH), 2.63 (t, *J* = 7.7 Hz, 2H, $ArCH_2$), 2.43 (td, *J* = 7.3, 1.8 Hz, 2H, C**H**$_2$CHO), 1.69-1.61 (m, 4H, 2 x CH_2), 1.40-1.36 (m, 4H, 2 x CH_2); ^{13}C NMR (125 MHz, $CDCl_3$) δ_C 202.8 (CH), 142.6 (C), 128.4 (2 x CH), 128.2 (2 x CH), 125.6 (CH), 43.9 (CH_2), 35.8 (CH_2), 31.2 (CH_2), 29.1(CH_2), 29.0 (CH_2), 22.0 (CH_2); LRMS (ES^+) calcd for $C_{13}H_{18}ONa$ $[M+Na]^+$: 213.12, found: 213.05.

1-Phenylpentadec-8-yn-7-ol (9). To a solution of 7-phenylheptanal (3 g, 15.7 mmol) in THF (50 mL) at −78 °C was added *n*-BuLi (10 mL, 1.58 mol/L) and warmed up to 0 °C over 30 mins. Then it was cooled down to −78 °C again and 1-octyne (1.73 g, 15.7 mmol) was added dropwise. The resulting mixture was stirred for 1 h at rt. The reaction was quenched with aq. NH_4Cl solution (50 mL) and extracted with EtOAc (3 × 20 mL). The combined organic layers were dried over $MgSO_4$ and concentrated in vacuo. Purification over silica gel, eluting with 5:1 hexane/EtOAc, yielded the alcohol (4.0 g, 85%) as a colourless oil; ^{1}H NMR (300 MHz, $CDCl_3$) δ_H 7.34-7.29 (m, 3H, ArH), 7.22-7.18 (m, 2H, ArH), 4.41-4.35 (m, 1H, C**H**OH), 2.64 (t, *J* = 7.4 Hz, 2H, $ArCH_2$), 2.27-2.10 (m, 2H, $\equiv CCH_2$), 1.76-1.61 (m, 6H, 3 x CH_2), 1.53-1.30 (m, 12H, 6 x CH_2), 0.93 (t, *J* = 6.4 Hz, 3H, CH_3); ^{13}C NMR (125 MHz, $CDCl_3$) δ_C 142.8 (C), 128.4 (2 x CH), 128.2 (2 x CH), 125.5 (CH), 85.6 (≡C), 81.2 (≡C), 62.7 (CH), 38.1 (CH_2), 35.9 (CH_2), 31.4 (CH_2), 31.3 (CH_2), 29.2 (CH_2), 29.1 (CH_2), 28.6 (CH_2), 28.5 (CH_2), 25.1 (CH_2), 22.5 (CH_2), 18.7 (CH_2), 14.0 (CH_3); HRMS (EI^+) calcd for $C_{21}H_{36}ON$ $[M+NH_4]^+$: 318.2791, found: 318.2784.

1-Phenylpentadec-8-yn-7-one (10). To a solution of 1-phenylpentadec-8-yn-7-ol (4 g, 13.3 mmol) in acetone (20 mL) at 0 °C was slowly added a solution of CrO_3 (1.5 g, 15 mmol) in H_2SO_4 (5 mL) and H_2O (10 mL). The mixture was stirred for 30 min at rt. The reaction was quenched with H_2O (20 mL) and extracted with diethyl ether (3 × 20 mL). The combined organic layers were washed with sat. $NaHCO_3$ solution, dried over $MgSO_4$ and concentrated in vacuo to give the ketone (3.6 g, 91%) as a yellow oil; ^1H NMR (300 MHz, $CDCl_3$) 7.31-7.26 (3H, m , ArH), 7.20-7.16 (2H, m, ArH), 2.61 (2H, t, J = 7.4 Hz, $ArCH_2$), 2.52 (2H, t, J = 7.4 Hz , $O{=}CCH_2$), 2.36 (2H, t, J = 7.0 Hz , $\equiv CCH_2$), 1.69-1.53 (8H, m, 4 x CH_2), 1.37-1.28 (8H, m, 4 x CH_2), 0.90 (3H, t, J = 6.8 Hz, CH_3); ^{13}C NMR (125 MHz, $CDCl_3$) δ_C 188.6 (C=O), 142.7 (C), 128.4 (2 x CH), 128.2 (2 x CH), 125.6 (CH), 94.4 (≡C), 80.9 (≡C), 45.5 (CH_2), 35.8 (CH_2), 31.3 (CH_2), 31.2 (CH_2), 29.0 (CH_2), 28.8 (CH_2), 28.5 (CH_2), 27.7 (CH_2), 24.1 (CH_2), 22.5 (CH_2), 18.9 (CH_2), 14.0 (CH_3); HRMS (EI^+) Exact mass calcd for $C_{21}H_{31}O$ $[M+H]^+$: 299.2369, found: 299.2367.

(7,7-Difluoropentadec-8-yn-1-yl)benzene (11). Neat DAST (2.33 mL, 17.6 mmol) was added to 1-phenylpentadec-8-yn-7-one (2.6 g, 8.8 mmol) under argon atmosphere. The mixture was heated at 50 °C for 4 h and cooled down to rt. It was diluted with EtOAc (50 mL), washed with saturated $NaHCO_3$ solution (100 mL). The organic layer was separated, dried over $MgSO_4$ and solvent removed in vacuo. Purification over silica with 2% EtOAc in hexane gave the difluoro product (1.9 g, 67%) as a colourless oil; $\{^{19}F\}^1H$ NMR (400 MHz, $CDCl_3$) 7.31-7.27 (3H, m , ArH), 7.27-7.17 (2H, m, ArH), 2.62 (2H, t, J = 7.4 Hz, $ArCH_2$), 2.27 (2H, t, J = 7.0 Hz , $\equiv CCH_2$), 2.02-1.96 (2H, m, CH_2), 1.66-1.50 (8H, m, 4 x CH_2), 1.39-1.28 (8H, m, 4 x CH_2), 0.90 (3H, t, J = 7.0 Hz, CH_3); ^{13}C NMR (125 MHz, $CDCl_3$) δ_C 142.6 (C), 128.4 (2 x CH), 128.2 (2 x CH), 125.6 (CH), 115.1 (t, J = 230 Hz, CF_2), 88.6 (≡C), 74.0 (≡C), 39.4 (t, J = 26.4 Hz, CH_2), 35.9 (CH_2), 31.3 (CH_2), 31.2 (CH_2), 29.0 (CH_2), 28.8 (CH_2), 28.4 (CH_2), 27.7 (CH_2), 24.1 (CH_2), 22.9 (t, J = 3.8 Hz, CH_2), 22.5 (CH_2), 18.3 (CH_2), 14.0 (CH_3); $\{^1H\}^{19}F$ NMR (376 MHz, $CDCl_3$) δ_F -81.3 (CF_2). HRMS (CI^+) Exact mass calcd for $C_{21}H_{30}F_2$ $[M^+]$: 320.2310, found: 320.2304.

(7,7-Difluoropentadecyl)benzene (12). Palladium on activated carbon (50 mg) was added to a solution of (7,7-difluoropentadec-8-yn-1-yl)benzene (400 mg, 1.25 mmol) in CH_2Cl_2 and stirred under hydrogen atmosphere for 18 h. The mixture was filtered through silica plug and solvent removed in vacuo. Purification over silica with 2% EtOAc in hexane gave the saturated product (380 mg, 94%) as a colourless oil; $\{^{19}F\}^1H$ NMR (500 MHz, $CDCl_3$) 7.29-7.26 (2H, m , ArH), 7.18-7.17 (3H, m, ArH), 2.60 (2H, t, J = 7.7 Hz , $ArCH_2$), 1.80-1.76 (4H, m, 2 x CH_2), 1.62 (2H, m, CH_2), 1.43 (4H, m, 2 x CH_2), 1.35 (4H, m, 2 x CH_2), 1.29-1.27 (10H, m, 5 x CH_2). 0.88 (3H, t, J = 6.6 Hz, CH_3); ^{13}C NMR (125 MHz, $CDCl_3$) δ_C 142.7 (C), 128.4 (2 x CH), 128.2 (2 x CH), 125.6 (CH), 125.4 (t, J = 240 Hz, CF_2), 36.2 (2 x t, J = 25.5 Hz, 2 x CH_2), 35.9 (CH_2), 31.8 (CH_2), 31.3 (CH_2), 29.4 (2 x CH_2), 29.2 (CH_2), 29.1 (CH_2), 29.0 (CH_2), 22.6 (CH_2), 22.3 (2 x t, J = 4.4 Hz, 2 x CH_2), 14.1 (CH_3); $\{^1H\}^{19}F$ NMR (470 MHz, $CDCl_3$) δ_F -97.6 (CF_2). HRMS (CI^+) Exact mass calcd for $C_{21}H_{34}F_2$ $[M^+]$: 324.2623, found: 324.2619.

8,8-Difluorohexadecanoic acid (6a). $RuCl_3{\cdot}xH_2O$ (20 mg, 0.08 mmol) and $NaIO_4$ (3.4 g, 16 mmol) was added to a solution of (7,7-difluoropentadecyl)benzene (300 mg, 0.92 mmol) in $CCl_4/MeCN/H_2O$ (2 mL/2 mL/3 mL) and stirred at 25 °C for 18 h. The mixture

acidified with 1 M HCl (1 mL), then filtered through a pad of celite, dried over $MgSO_4$ and concentrated in vacuo. Purification over silica with 20% EtOAc in hexane gave the acid (190 mg, 71%) as a white crystalline solid, m.p. 52 °C; $\{^{19}F\}^1H$ NMR (300 MHz, $CDCl_3$) 2.37 (2H, t, J = 7.4 Hz , CH_2), 1.82-1.77 (4H, m, 2 x CH_2), 1.70-1.61 (4H, m, 2 x CH_2), 1.48-1.42 (4H, m, 2 x CH_2), 1.38-1.29 (12H, m, 6 x CH_2), 0.89 (3H, t, J = 6.4 Hz, CH_3); ^{13}C NMR (75 MHz, $CDCl_3$) δ_C 178.6 (C=O), 125.4 (t, J = 238 Hz, CF_2), 36.3 (2 x t, J = 25.4 Hz, 2 x CH_2), 33.7 (CH_2), 31.8 (CH_2), 29.4 (CH_2), 29.3 (CH_2), 29.1 (CH_2), 29.0 (CH_2), 28.8 (CH_2), 24.5 (CH_2), 22.6 (CH_2), 22.3 (CH_2, t, J = 4.4 Hz,), 22.1 (CH_2, t, J = 4.7 Hz,), 14.1 (CH_3); $\{^1H\}^{19}F$ NMR (282 MHz, $CDCl_3$) δ_F -98.2 (CF_2). HRMS (ESI^-) Exact mass calcd for $C_{16}H_{29}F_4O_2$ $[M-H]^-$: 291.2141, found: 291.2133.

10-Phenyldec-1-en-4-ol (13). To a solution of 7-phenylheptanal (7.6 g, 40 mmol) in THF (200 mL) at 0 °C was added allylmagnesium bromide (45 mL, 45 mmol, 1.0 M in THF) and the resulting mixture was stirred for 30 min at rt. The reaction was quenched with aq. NH_4Cl solution (50 mL) and extracted with EtOAc (3 × 20 mL). The combined organic layers were dried over $MgSO_4$ and concentrated in vacuo. Purification over silica gel, eluting with 10:1 hexane/EtOAc, yielded the alcohol (5.5 g, 59%) as a colourless oil; ^1H NMR (400 MHz, $CDCl_3$) δ_H 7.29-7.25 (m, 2H, ArH), 7.18-7.16 (m, 3H, ArH), 5.88-5.77 (m, 1H, =CH), 5.16-5.14 (m, 1H, $=CH_2$), 5.12-5.10 (m, 1H, $=CH_2$), 3.66-3.60 (m, 1H, C**H**OH), 2.60 (t, J = 7.6 Hz, 2H, $ArCH_2$), 2.32-2.26 and 2.16-2.09 (m, 2H, HOCHC**H**$_2$), 1.64-1.43 (m, 6H, 3 x CH_2), 1.34-1.33 (m, 4H, 2 x CH_2); ^{13}C NMR (100 MHz, $CDCl_3$) δ_C 142.8 (C), 134.9 (CH), 128.4 (2 x CH), 128.2 (2 x CH), 125.5 (CH), 118.1 (CH_2), 70.6 (CHOH), 41.9 (CH_2), 36.7 (CH_2), 35.9 (CH_2), 31.4 (CH_2), 29.5 (CH_2), 29.2 (CH_2), 25.6 (CH_2); HRMS (ES^+) calcd for $C_{16}H_{28}ON$ $[M+NH_4]^+$: 250.2165, found: 250.2163.

10-Phenyldec-1-en-4-one (14). To a solution of 10-phenyldec-1-en-4-ol (5.5 g, 23.7 mmol) in dichlromethane (100 mL) at 0 °C was slowly added Dess-Martin periodinane (17 g, 40 mmol). The mixture was stirred for 30 min at room temperature. The reaction was quenched with H_2O (50 mL), washed with aqueous saturated $NaHCO_3$ solution (2 × 50 mL) and extracted with dichloromethane (3 × 20 mL). The combined organic layers were dried over $MgSO_4$ and concentrated in vacuo to give the ketone (4.5 g, 82%) as a colourless oil; ^1H NMR (400 MHz, $CDCl_3$) δ_H 7.20-7.16 (m, 2H, ArH), 7.08-7.07 (m, 3H, ArH), 5.88-5.78 (m, 1H, =CH), 5.10-5.01 (m, 2H, $=CH_2$), 3.07-3.05 (m, 2H, C**H**$_2$CH**=**), 2.50 (t, J = 7.7 Hz, 2H, $ArCH_2$), 2.33 (t, J = 7.4 Hz, 2H, CH_2C**=**O), 1.56-1.44 (m, 4H, 2 x CH_2), 1.25-1.22 (m, 4H, 2 x CH_2); ^{13}C NMR (100 MHz, $CDCl_3$) δ_C 208.7 (C=O), 142.6 (C), 130.6 (CH), 128.2 (2 x CH), 128.1 (2 x CH), 125.5 (CH), 118.6 (CH_2), 47.6 (CH_2), 42.2 (CH_2), 35.8 (CH_2), 31.2 (CH_2), 28.9 (2 x CH_2), 23.4 (CH_2); HRMS (ES^+) calcd for $C_{16}H_{23}O$ $[M+H]^+$: 231.1743, found: 231.1745.

(7,7-Difluorodec-9-en-1-yl)benzene (15). Neat DAST (6.4 g, 40 mmol) was added to 10-phenyldec-1-en-4-one (4.5 g, 19.6 mmol) under argon atmosphere. The reaction was heated at 50 °C for 4 hours and cooled down to room temperature. The reaction mixture was diluted with EtOAc (50 mL) and carefully quenched with saturated $NaHCO_3$ solution (100 mL). The organic layer was separated, dried over $MgSO_4$ and solvent removed in vacuo. Purification over silica with 2% EtOAc in hexane gave the difluoro

product (2.2 g, 45%) as a colourless oil ; {^{19}F}^{1}H NMR (500 MHz, $CDCl_3$) δ_H 7.23-7.19 (m, 2H, ArH), 7.21-7.20 (m, 3H, ArH), 5.87-5.78 (m, 1H, =CH), 5.24-5.20 (m, 2H, $=CH_2$), 2.65 (m, 2H, C**H**$_2$CH**=**), 2.62 (t, *J* = 6.1 Hz, 2H, $ArCH_2$), 1.84-1.80 (m, 2H, CH_2), 1.63 (m, 2H, CH_2), 1.50 (m, 2H, CH_2), 1.38 (m, 4H, 2 x CH_2); ^{13}C NMR (100 MHz, $CDCl_3$) δ_C 142.7 (C), 129.9 (t, *J* = 6 Hz, =CH), 128.4 (2 x CH), 128.2 (2 x CH), 125.6 (CH), 124.3 (t, *J* = 241 Hz, CF_2), 119.9 ($=CH_2$) , 65.9 (CH_2), 41.1 (t, *J* = 26 Hz, CH_2), 35.9 (CH_2), 31.6 (CH_2), 29.2 (CH_2), 22.6 (CH_2), 15.2 (CH_2); {^{1}H}^{19}F NMR (470 MHz, $CDCl_3$) δ_F -97.2 (CF_2); HRMS (ES$^+$) Exact mass calcd for $C_{16}H_{22}F_2$ [M$^+$]: 252.1684, found: 252.1683.

2-(2,2-Difluoro-8-phenyloctyl)oxirane (16). To a solution of (7,7-difluorodec-9-en-1-yl)benzene (2.0 g, 7.7 mmol) in dichloromethane (50 mL) was added mCPBA (6.9 g, 40 mmol) and the mixture was stirred for 10 hour at room temperature. The reaction mixture was washed with aqueous saturated $NaHCO_3$ solution (3 × 50 mL) and H_2O (50 mL), and extracted with dichloromethane (3 × 20 mL). The combined organic layers were dried over $MgSO_4$, concentrated in vacuo and purification over silica with 5% EtOAc/hexane to give the epoxide (1.5 g, 73%) as a colourless oil; ^{1}H NMR (300 MHz, $CDCl_3$) δ_H 7.30-7.26 (m, 2H, ArH), 7.20-7.16 (m, 3H, ArH), 3.14-3.08 (m, 1H, OCH), 2.84-2.81 (m, 1H, OCH), 2.61 (t, *J* = 7.7 Hz, $ArCH_2$), 2.53-2.51 (m, 1H, OCH), 2.18-1.81 (m, 4H, 2 x CH_2), 1.67-1.58 (m, 2H, CH_2), 1.52-1.47 (m, 2H, CH_2), 1.39-1.35 (m, 4H, 2 x CH_2); ^{13}C NMR (75 MHz, $CDCl_3$) δ_C 142.6 (C), 128.3 (2 x CH), 128.2 (2 x CH), 125.6 (CH), 124.2 (t, *J* = 241 Hz, CF_2), 46.9 (m, CH_2), 46.2 (CH_2), 39.9 (t, *J* = 25.8 Hz, CH_2), 36.6 (t, *J* = 24.6 Hz,CH_2), 35.8 (CH_2), 31.3 (CH_2), 29.1 (CH_2), 29.0 (CH_2), 22.0 (t, *J* = 4.5 Hz, CH_2); {^{1}H}^{19}F NMR (282 MHz, $CDCl_3$) δ_F -96.7 (q, *J* = 250 Hz, CF_2); HRMS (ES$^-$) Exact mass calcd for $C_{16}H_{21}OF_2$ [M-H]$^-$: 267.1555, found: 267.1553.

9,9-Difluoro-15-phenylpentadecan-7-ol (17). To a solution of 2-(2,2-difluoro-8-phenyloctyl)oxirane (1.0 g, 3.7 mmol) and CuI (50 mg) in THF (50 mL) at 0 °C was added pentylmagnesium chloride (2.5 mL, 5 mmol, 2.0 M in THF) and the resulting mixture was stirred for 30 min at rt. The reaction was quenched with aq. NH_4Cl solution (10 mL) and extracted with EtOAc (3 × 10 mL). The combined organic layers were dried over $MgSO_4$ and concentrated in vacuo. Purification over silica gel, eluting with 5% EtOAc/hexane, yielded the alcohol (0.65 g, 52%) as a colourless oil; {^{19}F}^{1}H NMR (500 MHz, $CDCl_3$) 7.33-7.30 (2H, m, ArH), 7.01-7.20 (3H, m, ArH), 4.05-4.02 (1H, m, CHOH), 2.64 (2H, t, *J* = 7.7 Hz, $ArCH_2$), 2.05-1.86 (4H, m, 2 x CH_2), 1.67-1.65 (2H, m, CH_2), 1.52-1.32 (16H, m, 8 x CH_2), 0.93 (3H, t, *J* = 6.4 Hz, CH_3); ^{13}C NMR (125 MHz, $CDCl_3$) δ_C 142.6 (C), 128.4 (2 x CH), 128.2 (2 x CH), 125.6 (CH), 125.5 (t, *J* = 241 Hz, CF_2), 66.8 (CH), 43.3 (t, *J* = 23.5 Hz, CH_2), 37.6 (CH_2), 37.2 (t, *J* = 25.0 Hz, CH_2), 35.9 (CH_2), 31.8 (CH_2), 31.3 (CH_2), 29.2 (CH_2), 29.1 (2 x CH_2), 25.3 (CH_2), 22.6 (CH_2), 22.2 (CH_2), 14.1 (CH_3); {^{1}H}^{19}F NMR (476 MHz, $CDCl_3$) δ_F -96.1 (abq, CF_2). HRMS (ESI$^+$) Exact mass calcd for $C_{21}H_{34}F_2ONa$ [M+Na]$^+$: 363.2471, found: 363.2464.

9,9-Difluoro-15-phenylpentadecan-7-one (18). To a solution of 9,9-difluoro-15-phenylpentadecan-7-ol (0.60 g, 1.8 mmol) in acetone (20 mL) at 0 °C was slowly added a solution of CrO_3 (0.4 g, 4 mmol) in H_2SO_4 (3 mL) and H_2O (6 mL). The mixture was stirred for 30 min at rt. The reaction was quenched with H_2O (20 mL) and extracted with

diethyl ether (3 × 20 mL). The combined organic layers were dried over $MgSO_4$ and concentrated in vacuo to give the ketone (0.50 g, 82%) as a colourless oil; $\{^{19}F\}^1H$ NMR (500 MHz, $CDCl_3$) 7.21-7.17 (2H, m , ArH), 7.11-7.08 (3H, m, ArH), 2.86 (2H, s, CH_2), 2.52 (2H, t, J = 7.7 Hz , $ArCH_2$), 2.42 (2H, t, J = 7.3 Hz, CH_2) 2.09 (2H, s, CH_2), 1.88-1.84 (2H, m, CH_2), 1.56-1.49 (4H, m, 2 x CH_2), 1.40-1.36 (2H, m, CH_2), 1.29-1.26 (4H, m, 2 x CH_2), 1.23-1.18 (6H, m, 3 x CH_2), 0.80 (3H, t, J = 6.8 Hz, CH_3); ^{13}C NMR (125 MHz, $CDCl_3$) δ_C 204.7 (C=O), 142.6 (C), 128.3 (2 x CH), 128.2 (2 x CH), 125.6 (CH), 122.8 (t, J = 242 Hz, CF_2), 48.7 (t, J = 26.2 Hz, CH_2), 44.2 (CH_2), 36.3 (t, J = 22.0 Hz, CH_2), 35.8 (CH_2), 31.5 (CH_2), 31.2 (CH_2), 29.0 (CH_2), 28.9 (CH_2), 28.6 (CH_2), 23.2 (CH_2), 22.4 (CH_2), 22.1 (t, J = 4.2 Hz, CH_2), 14.0 (CH_3); $\{^1H\}^{19}F$ NMR (476 MHz, $CDCl_3$) δ_F -93.3 (s, CF_2). HRMS (ESI^+) Exact mass calcd for $C_{21}H_{32}F_2ONa$ $[M+Na]^+$: 361.2315, found: 361.2312.

(7,7,9,9-Tetrafluoropentadecyl)benzene (19). Neat DAST (0.81 g, 5.0 mmol) was added to 9,9-difluoro-15-phenylpentadecan-7-one (0.50 g, 1.5 mmol) under argon atmosphere. The reaction was heated at 50 °C for 4 hours and cooled down to rt. The reaction mixture was diluted with EtOAc (20 mL), washed with saturated $NaHCO_3$ solution (50 mL) and extracted with EtOAc (2 × 20 mL). The organic layer was combined and dried over $MgSO_4$ and concentrated in vacuo. Purification over silica with 3% EtOAc in hexane gave the tetrafluoro product (0.32 g, 59%) as a colourless oil; $\{^{19}F\}^1H$ NMR (500 MHz, $CDCl_3$) 7.32-7.29 (2H, m , ArH), 7.22-7.19 (3H, m, ArH), 2.63 (2H, t, J = 7.7 Hz , $ArCH_2$), 2.46 (2H, s, CH_2), 1.95-1.92 (4H, m, 2 x CH_2), 1.68-1.63 (2H, m, CH_2), 1.52-1.47 (4H, m, 2 x CH_2), 1.38-1.33 (10H, m, 5 x CH_2), 0.92 (3H, t, J = 6.8 Hz, CH_3); ^{13}C NMR (125 MHz, $CDCl_3$) δ_C 142.6 (C), 128.4 (2 x CH), 128.2 (2 x CH), 125.6 (CH), 122.5 (2 x t, J = 242 Hz, 2 x CF_2), 42.3 (quin, J = 26.9 Hz, CH_2), 36.9 (2 x t, J = 23.8 Hz, 2 x CH_2), 35.9 (CH_2), 31.5 (CH_2), 31.3 (CH_2), 29.1 (CH_2), 29.0 (CH_2), 22.5 (CH_2), 22.0 (2 x CH_2), 14.0 (CH_3); $\{^1H\}^{19}F$ NMR (476 MHz, $CDCl_3$) δ_F -93.4 (m, 2 x CF_2). HRMS (ESI^+) Exact mass calcd for $C_{21}H_{32}F_4Na$ $[M+Na]^+$: 383.2322, found: 383.2332.

8,8,10,10-Tetrafluorohexadecanoic acid (6b). $RuCl_3 \cdot xH_2O$ (10 mg, 0.04 mmol) and $NaIO_4$ (1.7 g, 8 mmol) was added to a solution of (7,7,9,9-tetrafluoropentadecyl)benzene (150 mg, 0.42 mmol) in $CCl_4/MeCN/H_2O$ (2 mL/2 mL/3 mL) and stirred at 25 °C for 18 h. The mixture acidified with 1 M HCl (1 mL), then filtered through a pad of celite, dried over $MgSO_4$ and concentrated in vacuo. Purification over silica with 20% EtOAc in hexane gave the acid (95 mg, 69%) as a white crystalline solid, m.p. 60 °C; $\{^{19}F\}^1H$ NMR (500 MHz, $CDCl_3$) 2.45 (2H, s, CH_2), 2.36 (2H, t, J = 7.4 Hz, CH_2), 1.94-1.90 (4H, m, 2 x CH_2), 1.68-1.62 (2H, m, CH_2), 1.52-1.45 (4H, m, 2 x CH_2), 1.38-1.31 (10H, m, 5 x CH_2), 0.90 (3H, t, J = 6.9 Hz, CH_3); ^{13}C NMR (125 MHz, $CDCl_3$) δ_C 179.0 (C=O), 122.5 (2 x t, J = 245 Hz, 2x CF_2), 42.4 (t, J = 26.8 Hz, CH_2), 36.8 (m, 2 x CH_2), 33.8 (CH_2), 31.5 (CH_2), 28.9 (CH_2), 28.8 (CH_2), 28.7 (CH_2), 24.4 (CH_2), 22.5 (CH_2), 22.0 (CH_2), 21.9 (CH_2), 14.0 (CH_3); $\{^1H\}^{19}F$ NMR (476 MHz, $CDCl_3$) δ_F -93.4 (m, 2 x CF_2). HRMS (ESI^+) Exact mass calcd for $C_{16}H_{28}F_4O_2Na$ $[M+Na]^+$: 351.1916, found: 351.1917.

9-Phenylnon-1-yn-3-ol (20). To a solution of 7-phenylheptanal (4.4 g, 23 mmol) in THF (100 mL) at 0 °C was added ethynylmagnesium bromide (60 mL, 30 mmol, 0.5 M in

THF) and the resulting mixture was stirred for 30 min at rt. The reaction was quenched with aq. NH_4Cl solution (50 mL) and extracted with EtOAc (3 × 20 mL). The combined organic layers were dried over $MgSO_4$ and concentrated in vacuo. Purification over silica gel, eluting with 10:1 hexane/EtOAc, yielded the alcohol (3.6 g, 72%) as a colourless oil; ^{1}H NMR (400 MHz, $CDCl_3$) δ_H 7.22-7.19 (m, 3H, ArH), 7.12-7.09 (m, 2H, ArH), 4.32-4.27 (m, 1H, C**H**OH), 2.53 (t, J = 7.6 Hz, 2H, $ArCH_2$), 2.39 (d, J = 2.4 Hz, 1H, ≡CH), 1.72 (d, J = 5.5 Hz, 1H, OH), 1.67-1.61 (m, 2H, CH_2), 1.57-1.53 (m, 2H, CH_2), 1.41-1.37 (m, 2H, CH_2), 1.31-1.27 (m, 4H, 2 x CH_2); ^{13}C NMR (100 MHz, $CDCl_3$) δ_C 142.7 (C), 128.4 (2 x CH), 128.2 (2 x CH), 125.6 (CH), 84.9 (≡C), 72.9 (≡CH), 62.3 (CHOH), 37.6 (CH_2), 35.9 (CH_2), 31.4 (CH_2), 29.1 (CH_2), 29.0 (CH_2), 24.0 (CH_2); LRMS (ES^+) calcd for $C_{15}H_{20}ONa$ $[M+Na]^+$: 239.31, found: 239.06.

9-Phenylnon-1-yn-3-one (21). To a solution of 9-phenylnon-1-yn-3-ol (3.6 g, 16.6 mmol) in acetone (30 mL) at 0 °C was slowly added a solution of CrO_3 (2.0 g, 20 mmol) in H_2SO_4 (5 mL) and H_2O (10 mL). The mixture was stirred for 30 min at rt. The reaction was quenched with H_2O (50 mL) and extracted with diethyl ether (3 × 20 mL). The combined organic layers were dried over $MgSO_4$ and concentrated in vacuo to give the ketone (3.4 g, 96%) as a yellow oil; ^{1}H NMR (400 MHz, $CDCl_3$) δ_H 7.31-7.26 (m, 2H, ArH), 7.20-7.16 (m, 3H, ArH), 3.20 (s, 1H, ≡CH), 2.63-2.56 (m, 4H, 2 x CH_2), 1.71-1.60 (m, 4H, 2 x CH_2), 1.39-1.34 (m, 4H, 2 x CH_2); ^{13}C NMR (100 MHz, $CDCl_3$) δ_C 187.5 (C=O), 142.6 (C), 128.4 (2 x CH), 128.2 (2 x CH), 125.6 (CH), 81.4 (≡C), 78.5 (≡CH), 45.4 (CH_2), 35.9 (CH_2), 31.3 (CH_2), 29.0 (CH_2), 28.8 (CH_2), 23.7 (CH_2); LRMS (ES^+) calcd for $C_{15}H_{18}ONa$ $[M+Na]^+$: 237.12, found: 237.03.

(7,7-Difluoronon-8-yn-1-yl)benzene (22). Neat DAST (3.5 g, 20 mmol) was added to 9-phenylnon-1-yn-3-one (2.3 g, 10.7 mmol) under argon atmosphere. The mixture was heated at 50 °C for 4 hours and cooled down to rt. It was diluted with EtOAc (50 mL), washed with saturated $NaHCO_3$ solution (100 mL). The organic layer was separated, dried over $MgSO_4$ and solvent removed in vacuo. Purification over silica with 2% EtOAc in hexane gave the difluoro product (2.2 g, 87%) as a colourless oil ; ^{1}H NMR (400 MHz, $CDCl_3$) δ_H 7.31-7.18 (m, 5H, ArH), 2.75 (t, J = 5.0 Hz, ≡CH), 2.62 (t, J = 7.6 Hz , $ArCH_2$), 2.09-1.98 (m, CH_2), 1.68-1.53 (m, 2 x CH_2), 1.40-1.37 (m, 2 x CH_2); ^{13}C NMR (125 MHz, $CDCl_3$) δ_C 142.6 (C), 128.4 (2 x CH), 128.3 (2 x CH), 125.6 (CH), 114.4 (t, J = 233 Hz, CF_2), 87.9 (t, J = 7 Hz, ≡CH), 77.9 (t, J = 41 Hz, ≡C), 39.0 (t, J = 25.4 Hz, CH_2), 35.9 (CH_2), 31.2 (CH_2), 28.9 (CH_2), 28.8 (CH_2), 22.6 (t, J = 3.5 Hz, CH_2); HRMS (EI^+) Exact mass calcd for $C_{15}H_{18}F_2$ $[M^+]$: 236.1371, found: 236.1370.

9,9-Difluoro-15-phenylpentadec-7-yn-6-ol (23). To a solution of (7,7-difluoronon-8-yn-1-yl)benzene (1.7 g, 7.2 mmol) in THF (7 mL) cooled down to −78 °C was added BuLi (5 mL, 7.5 mmol, 1.5 M in THF) and the resulting mixture was warmed up to 0 °C over 10 mins. Then hexanal (0.75 g, 7.5 mmol) was added to the mixture and stirred for 1 hour at rt. The solvent was removed in vacuo and purification over silica with 5% EtOAc/hexane to give the product (1.3 g, 76%) as a colourless oil; {^{19}F}^{1}H NMR (300 MHz, $CDCl_3$) 7.32-7.17 (5H, m , ArH), 4.45 (1H, m, CHOH), 2.62 (2H, t, J = 7.5 Hz , $ArCH_2$), 2.05-2.00 (2H, m, CH_2), 1.86 (1H, d, J = 5.8 Hz, OH), 1.77-1.71 (2H, m, CH_2), 1.66-1.53 (4H, m, 2 x CH_2), 1.49-1.30 (12H, m, 6 x CH_2), 0.91 (3H, t, J = 6.7 Hz, CH_3);

^{13}C NMR (100 MHz, $CDCl_3$) δ_C 142.6 (C), 128.4 (2 x CH), 128.3 (2 x CH), 125.6 (CH), 114.8 (t, *J* = 232 Hz, CF_2), 87.9 (C), 77.9 (C), 62.1 (CH), 39.1 (t, *J* = 26.0 Hz, CH_2), 37.1 (CH_2), 35.8 (CH_2), 31.3 (CH_2), 31.2 (CH_2), 28.9 (CH_2), 28.8 (CH_2), 24.6 (CH_2), 22.7 (t, *J* = 3.2 Hz, CH_2), 22.5 (CH_2), 13.9 (CH_3); $\{^1H\}^{19}F$ NMR (376 MHz, $CDCl_3$) δ_F -83.1 (CF_2). HRMS (CI^+) Exact mass calcd for $C_{21}H_{34}NOF2$ $[M+NH_4]^+$: 354.2603, found: 354.2601.

9,9-Difluoro-15-phenylpentadec-7-yn-6-one (24). To a solution of 9,9-difluoro-15-phenylpentadec-7-yn-6-ol (1.3 g, 3.9 mmol) in acetone (20 mL) at 0 °C was slowly added a solution of CrO_3 (0.4 g, 4 mmol) in H_2SO_4 (3 mL) and H_2O (6 mL). The mixture was stirred for 30 min at rt. The reaction was quenched with H_2O (20 mL) and extracted with diethyl ether (3 × 20 mL). The combined organic layers were dried over $MgSO_4$ and concentrated in *vacuo* to give the ketone (1.2 g, 93%) as a yellow oil; $\{^{19}F\}^1H$ NMR (400 MHz, $CDCl_3$) 7.23-7.09 (5H, m , ArH), 2.54 (2H, t, *J* = 7.3 Hz , $ArCH_2$), 2.05-1.93 (2H, m, CH_2), 1.62-1.44 (6H, m, 3 x CH_2), 1.31-1.22 (10H, m, 5 x CH_2), 0.83 (3H, t, *J* = 6.9 Hz, CH_3); ^{13}C NMR (100 MHz, $CDCl_3$) δ_C 186.5 (C=O), 142.5 (C), 128.4 (2 x CH), 128.3 (2 x CH), 125.7 (CH), 114.5 (t, *J* = 235 Hz, CF_2), 82.3 (C), 80.6 (C), 45.4 (CH_2), 38.7 (t, *J* = 25.0 Hz, CH_2), 35.8 (CH_2), 31.2 (CH_2), 30.9 (CH_2), 28.9 (CH_2), 28.7 (CH_2), 23.2 (CH_2), 22.4 (t, *J* = 3.4 Hz, CH_2), 22.3 (CH_2), 13.8 (CH_3); $\{^1H\}^{19}F$ NMR (376 MHz, $CDCl_3$) δ_F -85.9 (CF_2). HRMS (ESI^+) Exact mass calcd for $C_{21}H_{29}F_2O$ $[M+H]^+$: 335.2181, found: 335.2179.

(7,7,10,10-Tetrafluoropentadec-8-yn-1-yl)benzene (25). Neat DAST (0.96 g, 6 mmol) was added to 9,9-difluoro-15-phenylpentadec-7-yn-6-one (1.0 g, 3 mmol) under argon atmosphere. The mixture was heated at 50 °C for 4 hours and cooled down to rt. It was diluted with EtOAc (50 mL), washed with saturated $NaHCO_3$ solution (100 mL). The organic layer was separated, dried over $MgSO_4$ and solvent removed in vacuo. Purification over silica with 2% EtOAc in hexane gave the tetrafluoro product (0.76 g, 71%) as a colourless oil; $\{^{19}F\}^1H$ NMR (400 MHz, $CDCl_3$) 7.23-7.09 (5H, m , ArH), 2.54 (2H, t, *J* = 7.6 Hz , $ArCH_2$), 2.00-1.96 (2H, m, CH_2), 1.60-1.44 (4H, m, 2 x CH_2), 1.32-1.26 (4H, m, 2 x CH_2), 1.31-1.28 (8H, m, 4 x CH_2). 0.84 (3H, t, *J* = 7.0 Hz, CH_3); ^{13}C NMR (100 MHz, $CDCl_3$) δ_C 142.5 (C), 128.4 (2 x CH), 128.3 (2 x CH), 125.7 (CH), 114.3 (t, *J* = 235 Hz, CF_2), 114.2 (t, *J* = 235 Hz, CF_2), 79.0 (t, *J* = 43.8 Hz, C), 78.9 (t, *J* = 44.0 Hz, C), 38.7 (m, 2 x CH_2), 35.8 (CH_2), 31.2 (CH_2), 30.9 (CH_2), 28.9 (CH_2), 28.7 (CH_2), 22.4 (CH_2), 22.3 (CH_2), 22.1 (CH_2), 13.8 (CH_3); $\{^1H\}^{19}F$ NMR (376 MHz, $CDCl_3$) δ_F -85.9 (CF_2). HRMS (EI^+) Exact mass calcd for $C_{21}H_{28}F_4$ [M^+]: 356.2122, found: 356.2125.

(7,7,10,10-Tetrafluoropentadecyl)benzene(26). Palladium on activated carbon (50 mg) was added to a solution of (7,7,10,10-tetrafluoropentadec-8-yn-1-yl)benzene (262 mg, 0.73 mmol) in CH_2Cl_2 and stirred under hydrogen atmosphere for 18 h. The mixture was filtered through silica plug and solvent removed in vacuo. Purification over silica with 2% EtOAc in hexane gave the saturated product (260 mg, 99%) as a white solid, m.p. 34 °C; $\{^{19}F\}^1H$ NMR (400 MHz, $CDCl_3$) 7.17-7.02 (5H, m , ArH), 2.47 (2H, t, *J* = 7.7 Hz , $ArCH_2$), 1.87 (2H, s, CH_2), 1.71-1.66 (4H, m, 2 x CH_2), 1.53-1.44 (2H, m, CH_2), 1.36-1.29 (4H, m, 2 x CH_2), 1.22-1.12 (6H, m, 3 x CH_2). 0.77 (3H, t, *J* = 6.5 Hz, CH_3); ^{13}C NMR (100 MHz, $CDCl_3$) δ_C 142.6 (C), 128.4 (2 x CH), 128.3 (2 x CH), 125.6 (CH), 124.5 (2 x t, *J* = 241 Hz, 2 x CF_2), 36.7 (2 x CH_2), 35.9 (CH_2), 31.5 (CH_2), 31.3 (CH_2),

29.2 (2 x CH_2), 29.0 (2 x CH_2), 22.4 (CH_2), 22.2 (t, J = 4.4 Hz, CH_2), 22.0 (t, J = 4.6 Hz, CH_2), 13.9 (CH_3); {^{1}H}^{19}F NMR (376 MHz, $CDCl_3$) δ_F -100.1 (2 x CF_2). HRMS (EI^+) Exact mass calcd for $C_{21}H_{32}F_4$ [M^+]: 360.2435, found: 360.2438.

8,8,11,11-Tetrafluorohexadecanoic acid (6c). $RuCl_3{\cdot}xH_2O$ (10 mg, 0.04 mmol) and $NaIO_4$ (1.7 g, 8 mmol) was added to a solution of (7,7,10,10-tetrafluoropentadecyl)benzene (140 mg, 0.4 mmol) in CCl_4/MeCN/H_2O (2 mL/2 mL/3 mL) and stirred at 25 °C for 18 h. The mixture acidified with 1 M HCl (1 mL), then filtered through a pad of celite, dried over $MgSO_4$ and concentrated in vacuo. Purification over silica with 20% EtOAc in hexane gave the acid (100 mg, 76%) as a white crystalline solid, m.p. 79 °C; {^{19}F}^{1}H NMR (400 MHz, $CDCl_3$) 2.37 (2H, t, J = 7.4 Hz , CH_2), 2.02 (4H, s, 2 x CH_2), 1.86-1.81 (4H, m, 2 x CH_2), 1.70-1.62 (4H, m, 2 x CH_2), 1.52-1.42 (4H, m, 2 x CH_2), 1.38-1.22 (6H, m, 3 x CH_2), 0.91 (3H, t, J = 6.9 Hz, CH_3); ^{13}C NMR (100 MHz, $CDCl_3$) δ_C 179.1 (C=O), 124.5 (t, J = 241 Hz, CF_2), 124.4 (t, J = 241 Hz, CF_2), 36.7 (2 x t, J = 25.1 Hz, 2 x CH_2), 33.8 (CH_2), 31.5 (CH_2), 29.1 (2 x CH_2), 28.9 (CH_2), 28.7 (CH_2), 24.4 (CH_2), 22.4 (CH_2), 22.1 (CH_2), 22.0 (CH_2), 13.9 (CH_3); {^{1}H}^{19}F NMR (376 MHz, $CDCl_3$) δ_F -100.1 (CF_2), -100.2 (CF_2). HRMS (ESI^-) Exact mass calcd for $C_{16}H_{27}F_4O_2$ $[M-H]^-$: 327.1953, found: 327.1946.

7-Octenal (28). To a solution of 7-octen-1-ol (6.0 g, 46.9 mmol) in DMSO (219 mL) was added IBX[1] (15.8, 56.3 mmol) and the resulting mixture was stirred 18h at rt. The reaction was quenched with water (516 mL) and EtOAc (516 mL). The precipitated white solid was filtered and the filtrate collected. The organic layer was separated and washed with water (2 × 516 mL) and brine (516 mL), dried over anhydrous $MgSO_4$ and the solvent was removed in vacuo to give 7-octenal (5.73 g, 97%) as a yellow oil, which was used without further purification.[2]

9-Decen-1-yn-3-ol (29). To a solution of 7-octenal (3.68 g, 29.2 mmol) in THF (103 mL) at 0 °C was added ethynylmagnesium bromide (70 mL, 35 mmol, 0.5 M in THF) and the resulting mixture was stirred for 3h at rt. The reaction was quenched with aq. NH_4Cl solution (170 mL) and diethyl ether (84 mL). The aqueous layer was extracted with diethyl ether (3 × 56 mL). The combined organic layers were dried over $MgSO_4$ and concentrated in vacuo. Purification over silica gel, eluting with 5:1 hexane/EtOAc, yielded the alcohol (2.93 g, 66%) as a yellow oil; ^{1}H NMR (500 MHz, $CDCl_3$) δ_H 5.81 (1H, ddt, J = 17.0, 10.2, 6.7 Hz, =CH), 4.93-5.02 (2H, m, =CH_2), 4.37 (1H, td, J = 6.6, 2.0 Hz, OCH), 2.47 (1H, d, J = 2.2 Hz, ≡CH), 2.03-2.08 (2H, m, CH_2), 1.95 (1H, bs, OH), 1.67-1.78 (2H, m, CH_2), 1.32-1.51 (6H, m, 3 x CH_2); ^{13}C NMR (125 MHz, $CDCl_3$) δ_C 138.9 (=CH), 114.3 (=CH_2), 84.9 (≡C), 72.8 (≡CH), 62.2 (OCH), 37.5 (CH_2), 33.6 (CH_2), 28.7 (CH_2), 28.6 (CH_2), 24.8 (CH_2); HRMS (CI^+) Exact mass calcd for $C_{10}H_{17}O$ $[M+H]^+$: 153.1274, found: 153.1270.

9-Decen-1-yn-3-one (30). To a solution of 9-decen-1-yn-3-ol (1.41 g, 9.25 mmol) in DMSO (43 mL) was added IBX (3.11 g, 11.1 mmol) and the resulting mixture was stirred

---

[1] Frigerio, M.; Santagostino, M.; Sputore, S. *J. Org. Chem.* **1999**, *64,* 4537-4538.
[2] Rosenquist, A.; Samuelsson, B. *et al*. *Bioorg. Med. Chem.* **2007**, *15*, 7184-7202.

18 h at rt. The reaction was quenched with water (102 mL) and EtOAc (102 mL). The precipitated white solid was filtered and the filtrate collected. The organic layer was separated and washed with water (2 × 102 mL) and brine (102 mL), dried over anhydrous $MgSO_4$ and the solvent was removed in vacuo to give the propargylic ketone (1.19 g, 85%) as a yellow oil, which was used without further purification; ^1H NMR (300 MHz, $CDCl_3$) δ_H 5.80 (1H, ddt, *J* = 17.0, 10.2, 6.7 Hz, =CH), 4.93-5.04 (2H, m, =CH_2), 3.21 (1H, s, ≡CH), 2.60 (2H, t, *J* = 7.4 Hz, CH_2), 2.02-2.10 (2H, m, CH_2), 1.65-1.75 (2H, m, CH_2), 1.27-1.47 (4H, m, 2 x CH_2); ^{13}C NMR (75 MHz, $CDCl_3$) δ_C 187.5 (C=O), 138.6 (=CH), 114.5 (=CH_2), 81.4 (≡C), 78.3 (≡CH), 45.3 (CH_2), 33.4 (CH_2), 28.5 (CH_2), 28.3 (CH_2), 23.5 (CH_2); HRMS (CI^+) Exact mass calcd for $C_{10}H_{15}O$ $[M+H]^+$: 151.1117, found: 151.1114.

8,8-Difluoro-1-decen-9-yne (31). Neat DAST (7.3 mL, 8.9 g, 55.2 mmol) was added to 9-Decen-1-yn-3-one (2.07 g, 13.8 mmol) under argon atmosphere. The mixture was heated at 50 °C for 18 hours and cooled down to rt. Crude reaction was added **portionwise to a biphasic mixture of saturated aqueous $NaHCO_3$ solution (148 mL) and** pentane (74 mL) at 0 °C. The aqueous layer was separated and extracted with pentane (3 × 49 mL). The combined organic extracts were dried over $MgSO_4$ and concentrated by Vigreux distillation at atmospheric pressure. The concentrate was purified over silica gel, eluting with pentane. The solvent was removed by Vigreux distillation at atmospheric pressure yielding the highly volatile difluoro product (1.5 g, 63%) as a colourless liquid, which was rapidly used in the next step; ^1H NMR (500 MHz, $CDCl_3$) δ_H 5.81 (1H, ddt, *J* = 17.0, 10.3, 6.7 Hz, =CH), 4.95-5.04 (2H, m, =CH_2), 2.76 (1H, t, *J* = 4.9 Hz, ≡CH), 2.00-2.09 (4H, m, 2 x CH_2), 1.52-1.61 (2H, m, CH_2), 1.36-1.46 (4H, m, 2 x CH_2); $\{^{19}F\}^1H$ NMR (500 MHz, $CDCl_3$) δ_H 5.81 (1H, ddt, *J* = 17.0, 10.3, 6.7 Hz, =CH), 4.95-5.04 (2H, m, =CH_2), 2.76 (1H, s, ≡CH), 2.02-2.09 (4H, m, 2 x CH_2), 1.52-1.61 (2H, m, CH_2), 1.36-1.46 (4H, m, 2 x CH_2); ^{13}C NMR (125 MHz, $CDCl_3$) δ_C 138.7 (=CH), 114.5 (=CH_2), 114.3 (t, *J* = 233 Hz, CF_2), 76.6 (t, *J* = 41.4 Hz, ≡C), 75.0 (t, *J* = 6.8 Hz, ≡CH), 38.9 (t, *J* = 25.5 Hz, CH_2), 33.5 (CH_2), 28.5 (CH_2), 28.3 (CH_2), 22.5 (t, *J* = 3.4 Hz, CH_2); $\{^1H\}^{19}F$ NMR (470 MHz, $CDCl_3$) δ_F -84.0 (CF_2); ^{19}F NMR (470 MHz, $CDCl_3$) δ_F -84.0 (td, *J* = 15.0, 4.7 Hz, CF_2).

8-Nonenal (32). To a solution of 8-nonen-1-ol (1.7 g, 12.0 mmol) in DMSO (56 mL) was added IBX (8.0 g, 28.8 mmol) and the resulting mixture was stirred 18h at rt. The reaction was quenched with water (133 mL) and EtOAc (133 mL). The precipitated white solid was filtered and the filtrate collected. The organic layer was separated and washed with water (2 × 133 mL) and brine (133 mL), dried over anhydrous $MgSO_4$ and the solvent was removed in vacuo. Purification over silica gel, eluting with 7:1 hexane/EtOAc, yielded 8-nonenal (1.45 g, 87%) as a yellow oil.[3]

12,12-Difluorononadeca-1,18-dien-10-yn-9-ol (33). To a solution of 8,8-difluoro-1-decen-9-yne (1.5 g, 8.72 mmol) in THF (13.1 mL) under argon atmosphere cooled down to -78 °C was added BuLi (6 mL, 9.6 mmol, 1.6 M in THF) and the resulting mixture was warmed up to 0 °C over 1h. Then a solution of 8-nonenal (1.34 g, 9.6 mmol) in THF (13.1 mL) was added to the mixture and stirred for 1 hour at rt. The reaction was

[3] Zakrzewski, J.; Grodner, J.; Bobbitt, J. M.; Karpińska, M. *Synthesis* **2007**, 2491-2494.

quenched with aq. NH_4Cl solution (29 mL) and extracted with EtOAc (3 × 12 mL). The combined organic layers were dried over $MgSO_4$ and concentrated in vacuo. Purification over silica gel, eluting with 7:1 hexane/EtOAc, yielded the alcohol (1.93 g, 71%) as a yellow oil; ^1H NMR (500 MHz, $CDCl_3$) δ_H 5.77-5.86 (2H, m, 2 x =CH), 4.93-5.04 (4H, m, 2 x =CH_2), 4.43-4.48 (1H, m, OCH), 1.98-2.09 (6H, m, 3 x CH_2), 1.80 (1H, d, J = 6.0 Hz, OH), 1.72-1.78 (2H, m, CH_2), 1.33-1.59 (14H, m, 7 x CH_2); {^{19}F}^1H NMR (500 MHz, $CDCl_3$) δ_H 5.76-5.85 (2H, m, 2 x =CH), 4.93-5.03 (4H, m, 2 x =CH_2), 4.45 (1H, q, J = 6.2 Hz, OCH), 2.01-2.09 (6H, m, 3 x CH_2), 1.69-1.80 (2H, m, CH_2), 1.33-1.59 (14H, m, 7 x CH_2); ^{13}C NMR (125 MHz, $CDCl_3$) δ_C 139.0 (=CH), 138.6 (=CH), 114.8 (t, J = 232 Hz, CF_2), 114.5 (=CH_2), 114.2 (=CH_2), 87.8 (t, J = 6.5 Hz, ≡C), 77.9 (t, J = 40.9 Hz, ≡C), 62.0 (OCH), 39.1 (t, J = 26.0 Hz, CH_2), 37.0 (CH_2), 33.7 (CH_2), 33.5 (CH_2), 29.0 (CH_2), 28.9 (CH_2), 28.7 (CH_2), 28.5 (CH_2), 28.3 (CH_2), 24.8 (CH_2), 22.6 (t, J = 3.2 Hz, CH_2); {^1H}^{19}F NMR (470 MHz, $CDCl_3$) δ_F -82.7 (CF_2); ^{19}F NMR (470 MHz, $CDCl_3$) δ_F -82.7 (td, J = 14.9, 3.8 Hz, CF_2); HRMS (CI^+) Exact mass calcd for $C_{19}H_{34}OF_2N$ $[M+NH_4]^+$: 330.2603, found: 330.2603.

12,12-Difluorononadeca-1,18-dien-10-yn-9-one (34). To a solution of 12,12-difluorononadeca-1,18-dien-10-yn-9-ol (1.93 g, 6.19 mmol) in DMSO (29.1 mL) was added IBX (2.1 g, 7.42 mmol) and the resulting mixture was stirred 18h at rt. The reaction was quenched with water (68 mL) and EtOAc (68 mL). The precipitated white solid was filtered and the filtrate collected. The organic layer was separated and washed with water (2 × 68 mL) and brine (68 mL), dried over anhydrous $MgSO_4$ and the solvent was removed in vacuo to give the propargylic ketone (1.58 g, 82%) as a yellow oil, which was used without further purification; ^1H NMR (500 MHz, $CDCl_3$) δ_H 5.76-5.85 (2H, m, 2 x =CH), 4.93-5.04 (4H, m, 2 x =CH_2), 2.63 (2H, t, J = 7.4 Hz, CH_2), 2.03-2.13 (6H, m, 3 x CH_2), 1.66-1.72 (2H, m, CH_2), 1.53-1.60 (2H, m, CH_2), 1.32-1.46 (10H, m, 5 x CH_2); ^{13}C NMR (125 MHz, $CDCl_3$) δ_C 186.4 (C=O), 138.8 (=CH), 138.5 (=CH), 114.6 (=CH_2), 114.5 (t, J = 235 Hz, CF_2), 114.4 (=CH_2), 82.3 (t, J = 6.5 Hz, ≡C), 80.6 (t, J = 42.3Hz, ≡C), 45.4 (CH_2), 38.7 (t, J = 25.1 Hz, CH_2), 33.6 (CH_2), 33.4 (CH_2), 28.7 (CH_2), 28.63 (CH_2), 28.58 (CH_2), 28.4 (CH_2), 28.3 (CH_2), 23.5 (CH_2), 22.3 (t, J = 3.2 Hz, CH_2); {^1H}^{19}F NMR (470 MHz, $CDCl_3$) δ_F -85.4 (CF_2); ^{19}F NMR (470 MHz, $CDCl_3$) δ_F -85.4 (t, J = 15.2 Hz, CF_2); HRMS (CI^+) Exact mass calcd for $C_{19}H_{32}OF_2N$ $[M+NH_4]^+$: 328.2446, found: 328.2443.

8,8,11,11-Tetrafluorononadeca-1,18-dien-9-yne (35). Neat DAST (0.5 mL, 0.6 g, 3.7 mmol) was added to 12,12-difluorononadeca-1,18-dien-10-yn-9-one (0.28 g, 0.91 mmol) under argon atmosphere. The mixture was heated at 50 °C for 18 hours and cooled down to rt. The reaction mixture was diluted with DCM (3.3 mL), washed with saturated $NaHCO_3$ solution (3.3 mL) and extracted with DCM (2 × 3.3 mL). The organic layer was combined and dried over $MgSO_4$ and concentrated in vacuo. Purification over silica gel with hexane gave the tetrafluoro product (0.21 g, 69%) as a colourless oil; ^1H NMR (500 MHz, $CDCl_3$) δ_H 5.76-5.85 (2H, m, 2 x =CH), 4.94-5.04 (4H, m, 2 x =CH_2), 2.02-2.16 (8H, m, 4 x CH_2), 1.52-1.59 (4H, m, 2 x CH_2), 1.30-1.46 (10H, m, 5 x CH_2); ^{13}C NMR (125 MHz, $CDCl_3$) δ_C 138.8 (=CH), 138.5 (=CH), 114.6 (=CH_2), 114.4 (=CH_2), 114.2 (t, J = 236 Hz, 2 x CF_2), 79.0 (t, J = 42.5 Hz, ≡C), 78.9 (t, J = 42.3Hz, ≡C), 38.7 (t, J = 24.0 Hz, 2 x CH_2), 33.6 (CH_2), 33.4 (CH_2), 28.7 (2 x CH_2), 28.6 (CH_2), 28.4 (CH_2), 28.2 (CH_2),

22.4 (t, J = 3.0 Hz, CH_2), 22.3 (t, J = 3.1 Hz, CH_2); {^{1}H}^{19}F NMR (470 MHz, $CDCl_3$) δ_F -85.36 (t, J = 4.5 Hz, CF_2), -85.39 (t, J = 4.5 Hz, CF_2); ^{19}F NMR (470 MHz, $CDCl_3$) δ_F -85.37 (tt, J = 16.0, 5.0 Hz, CF_2), -85.40 (tt, J = 15.9, 4.6 Hz, CF_2).

8,8,11,11-Tetrafluorononadecane (27). Palladium on activated carbon (21.2 mg) was added to a solution of 8,8,11,11-tetrafluorononadeca-1,18-dien-9-yne (33.0 mg, 0.1 mmol) in ethanol (1.72 mL) and stirred under hydrogen atmosphere for 18 h. The mixture was diluted with EtOAc (3 mL), filtered through a short plug of Celite and the solvent was removed in vacuo. Purification over silica gel with hexane gave the tetrafluoro alkane (29.7 mg, 90%) as a white solid, m.p. 35-37 °C; ^{1}H NMR (500 MHz, $CDCl_3$) δ_H 1.97-2.07 (2H, m, CH_2), 1.75-1.88 (4H, m, 2 x CH_2), 1.42-1.51 (4H, m, 2 x CH_2), 1.29-1.33 (20H, m, 10 x CH_2), 0.89 (6H, t, J = 7.1 Hz, 2 x CH_3); ^{13}C NMR (125 MHz, $CDCl_3$) δ_C 124.5 (t, J = 241 Hz, 2 x CF_2), 36.8 (t, J = 25.3 Hz, 2 x CH_2), 31.8 (CH_2), 31.7 (CH_2), 29.32 (3 x CH_2), 29.29 (CH_2), 29.1 (2 x CH_2), 29.0 (CH_2), 22.64 (CH_2), 22.60 (CH_2), 22.3 (t, J = 4.0 Hz, 2 x CH_2), 14.08 (CH_3), 14.06 (CH_3); {^{1}H}^{19}F NMR (470 MHz, $CDCl_3$) δ_F -99.6 (2 x CF_2).

NMR spectra

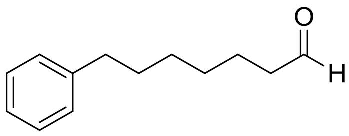

7-phenylheptanal (8).

^{1}H-NMR

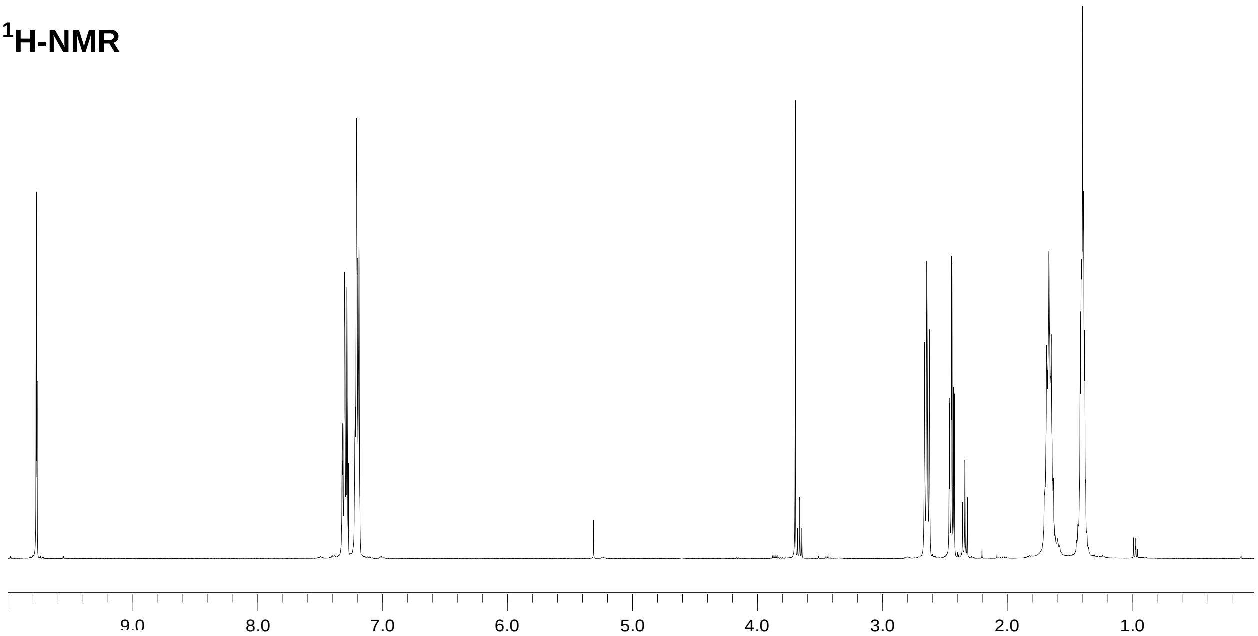

^{13}C-NMR

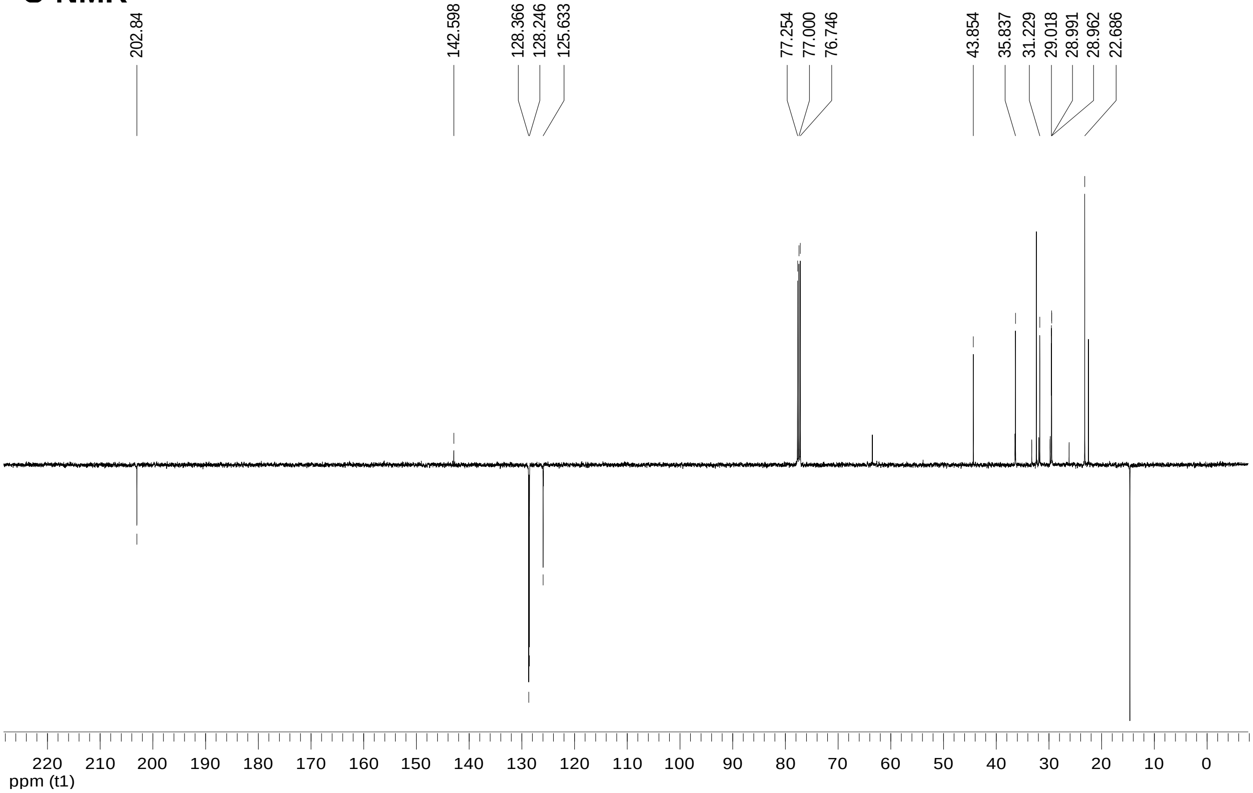

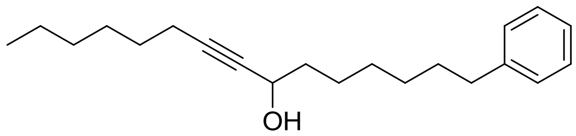

1-phenylpentadec-8-yn-7-ol (9).

^{1}H-NMR

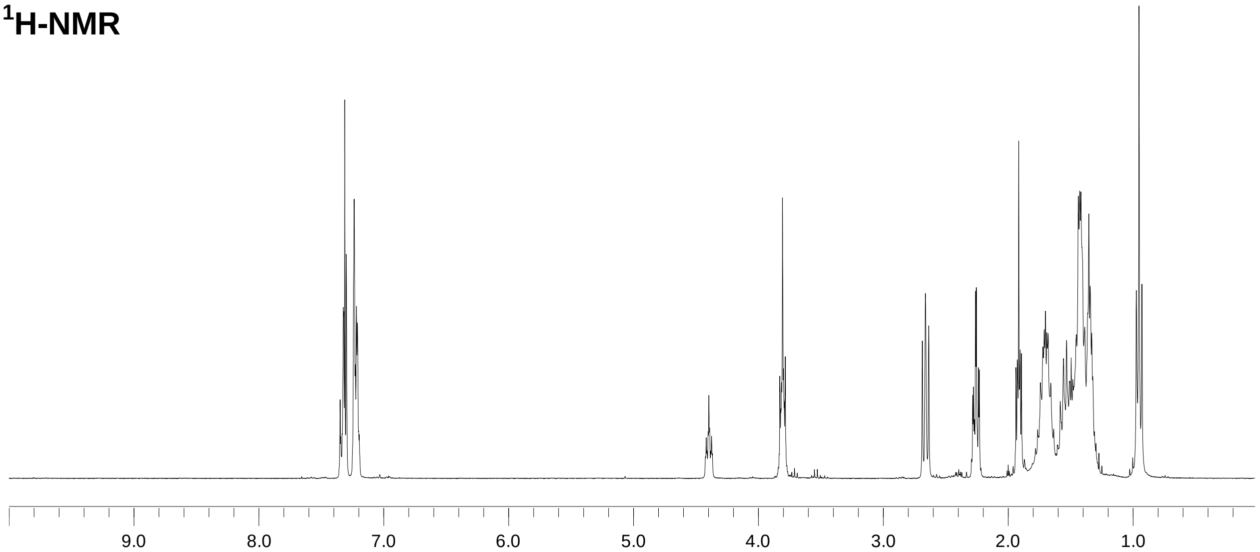

^{13}C-NMR

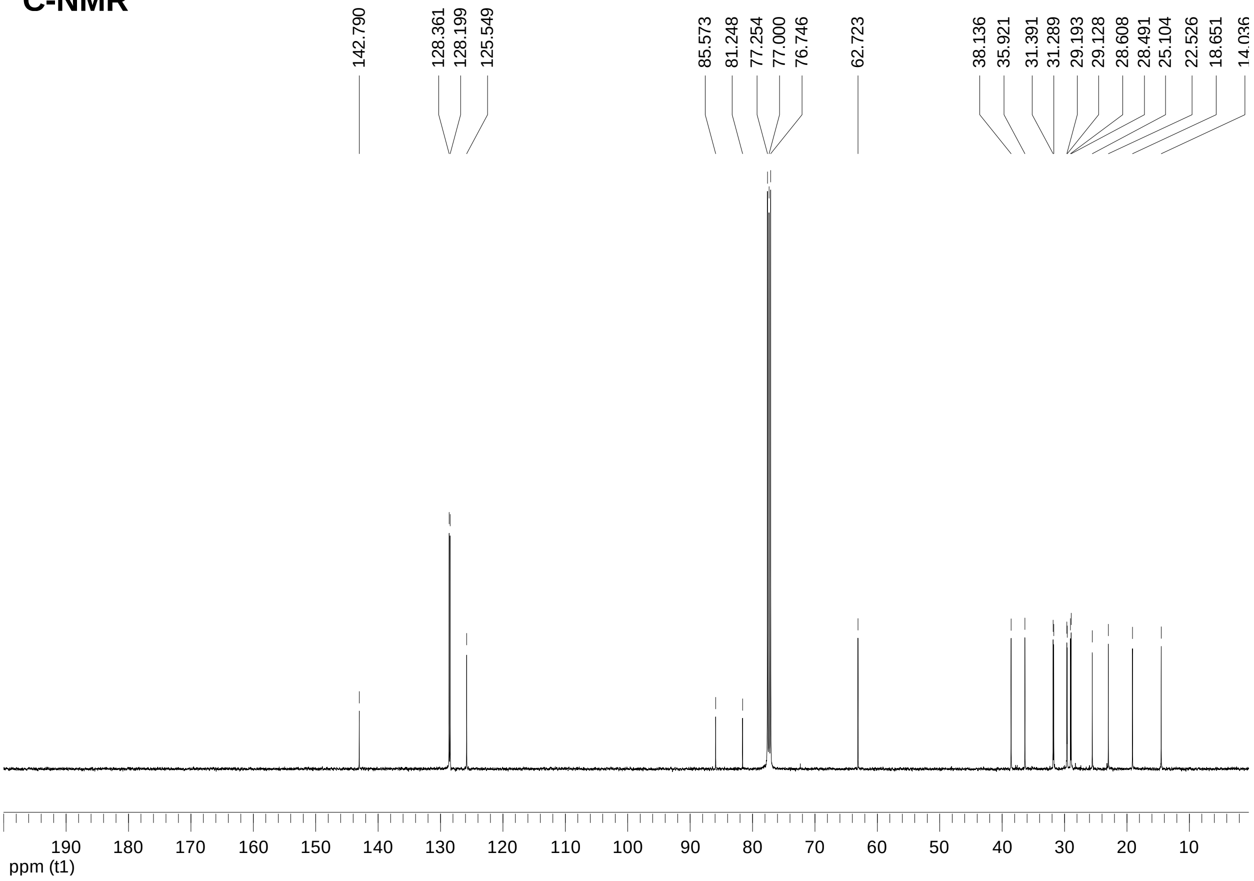

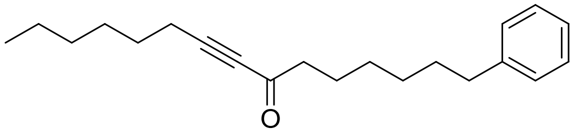

1-phenylpentadec-8-yn-7-one (10).

^{1}H-NMR

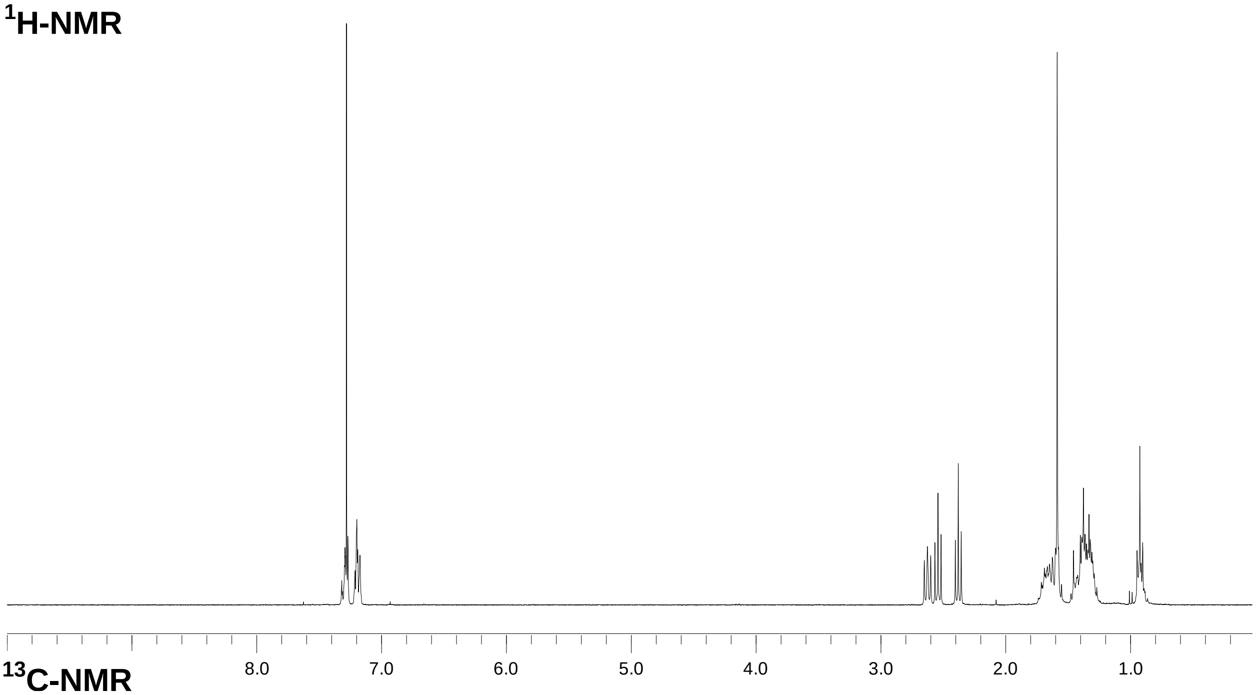

^{13}C-NMR

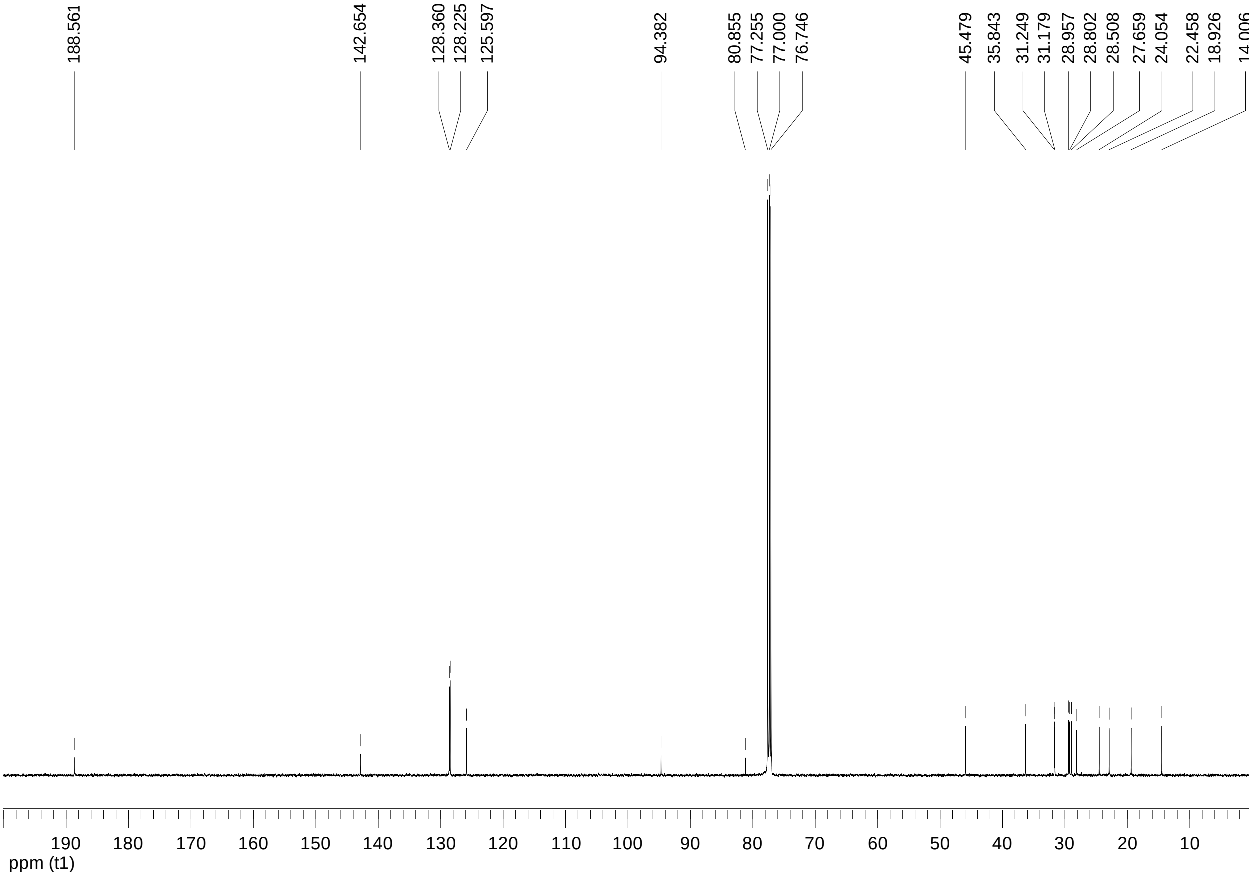

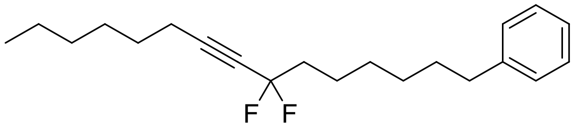

(7,7-Difluoropentadec-8-yn-1-yl)benzene (11).

^{1}H-NMR

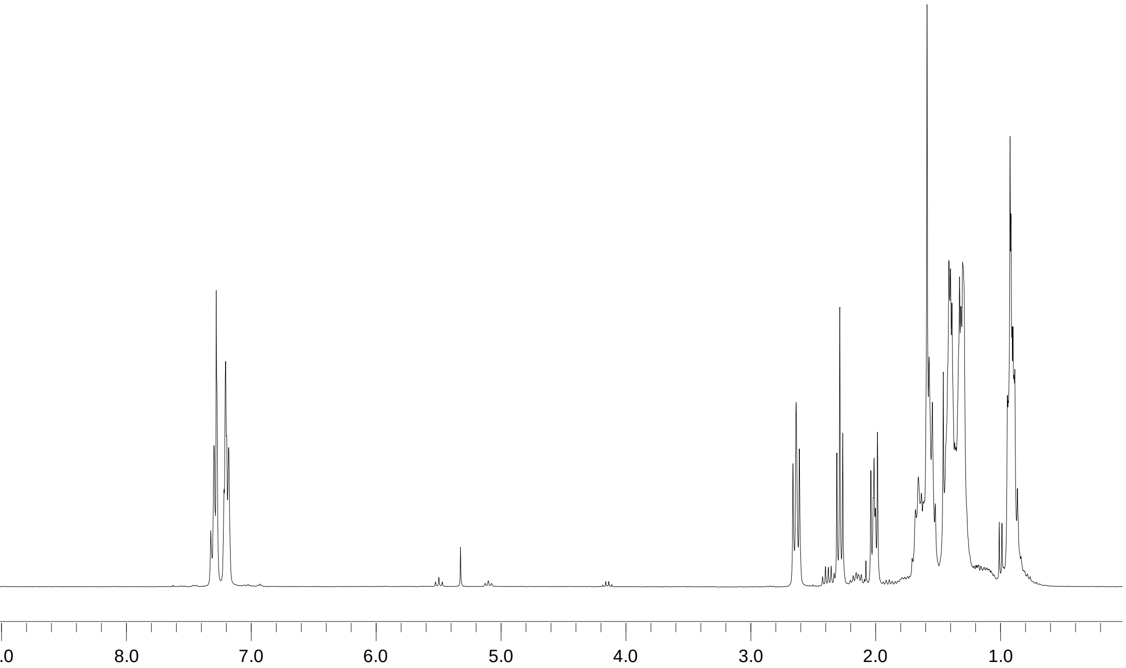

^{13}C-NMR

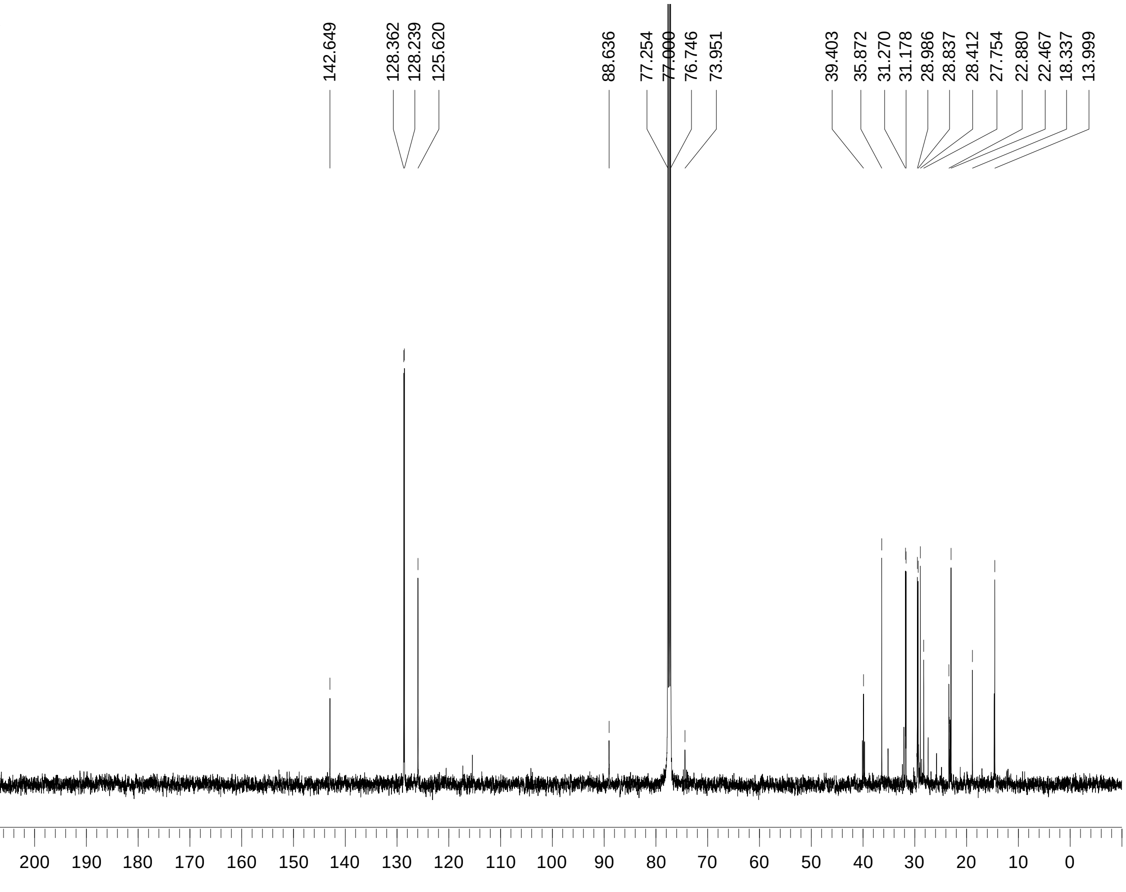

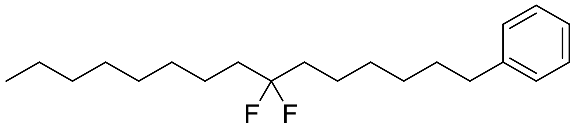

(7,7-Difluoropentadecyl)benzene (12).

^{1}H-NMR

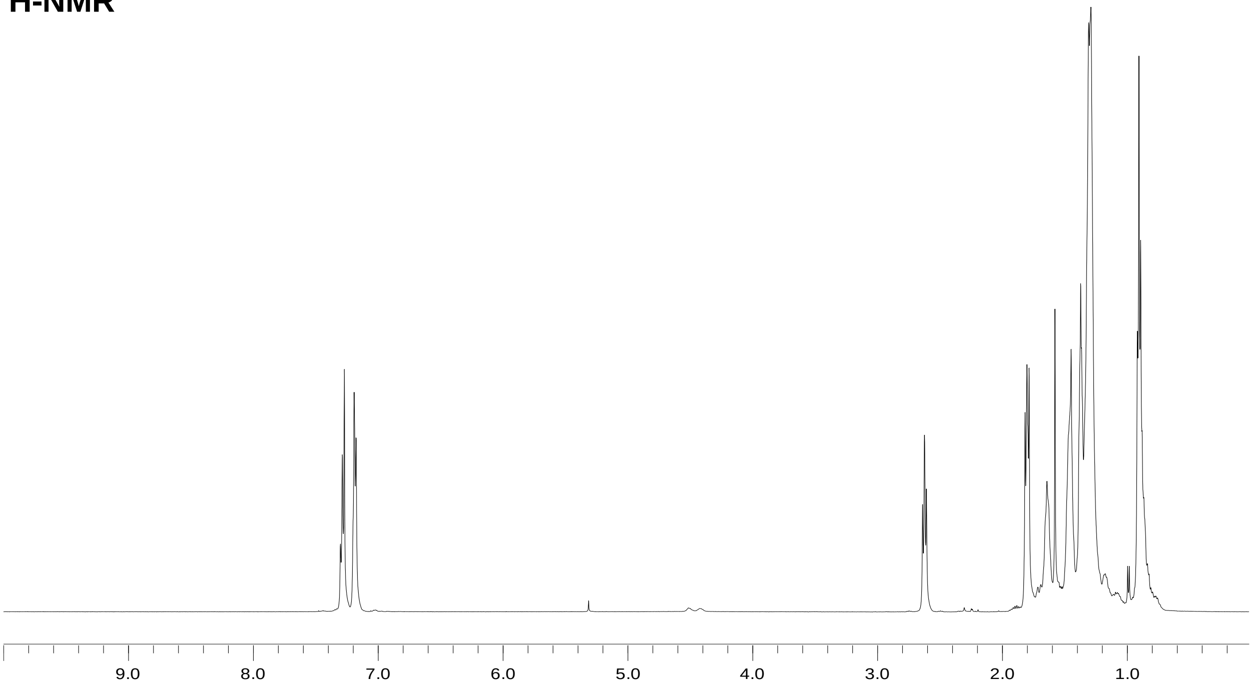

^{13}C-NMR

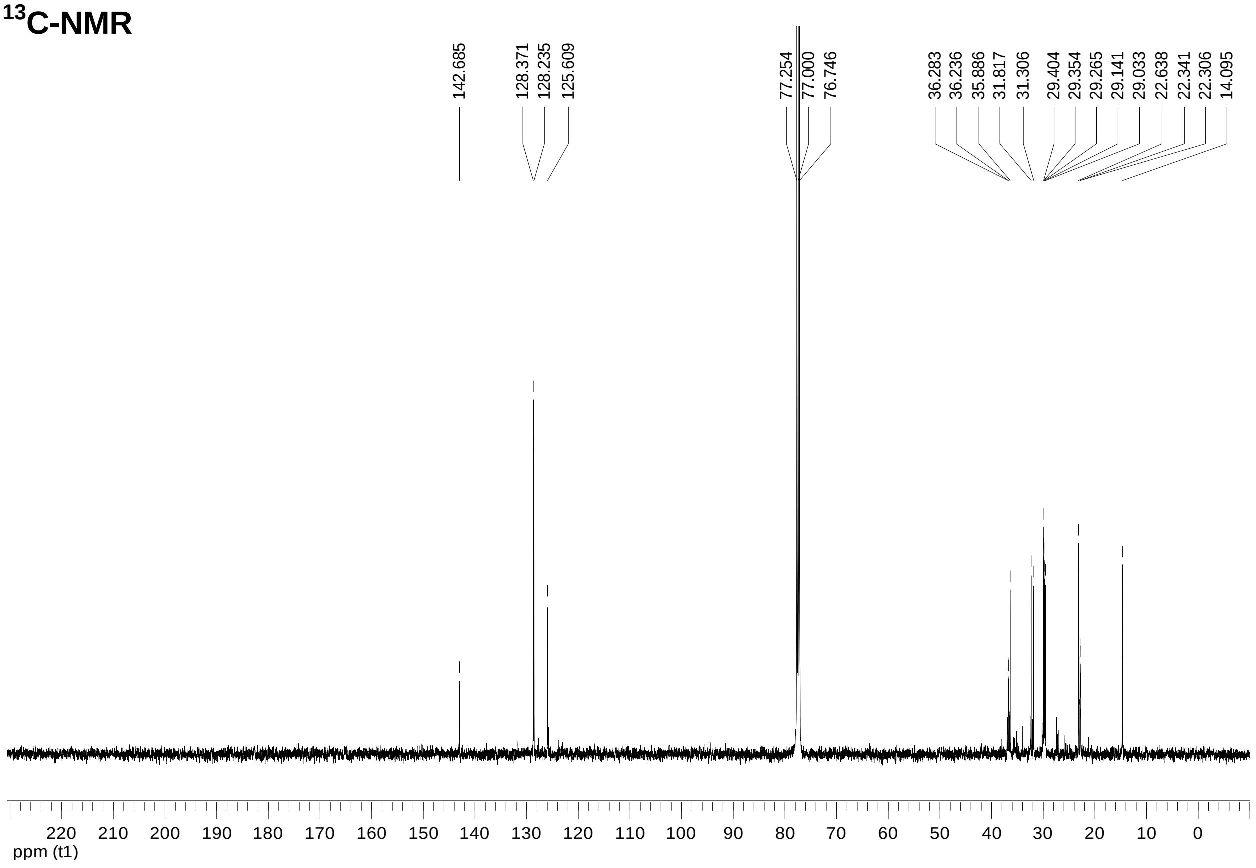

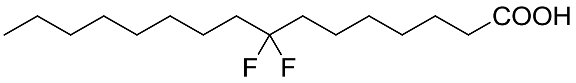

8,8-Difluorohexadecanoic acid (6a).

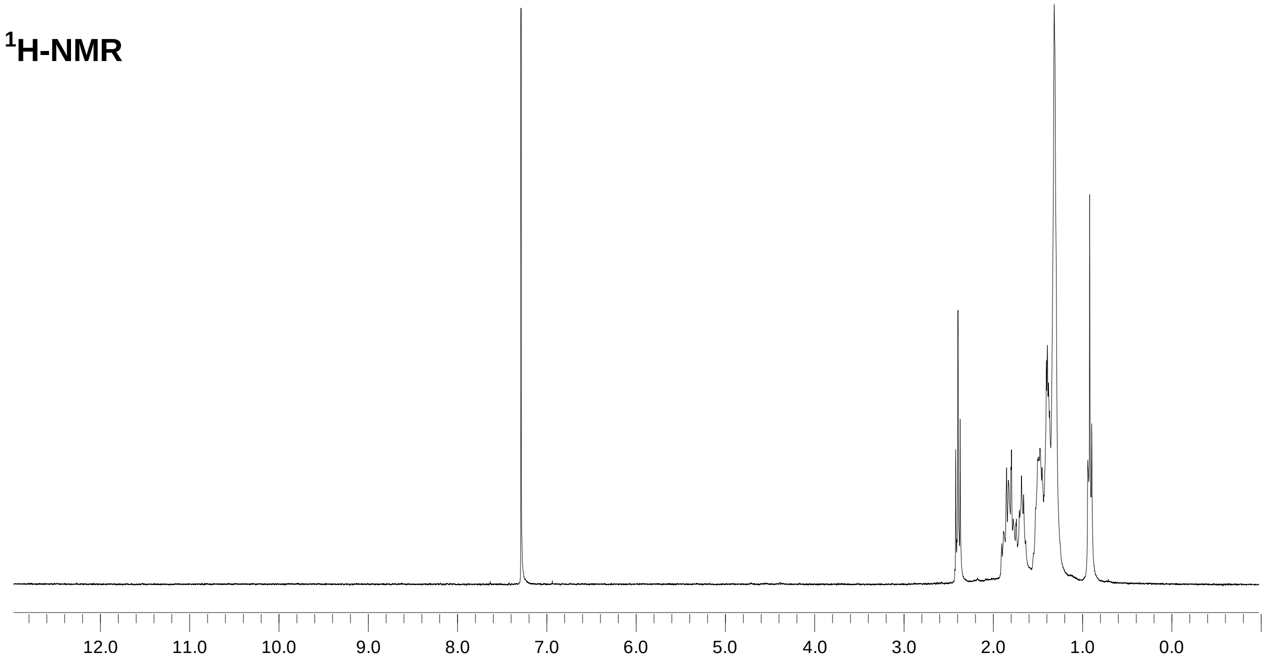

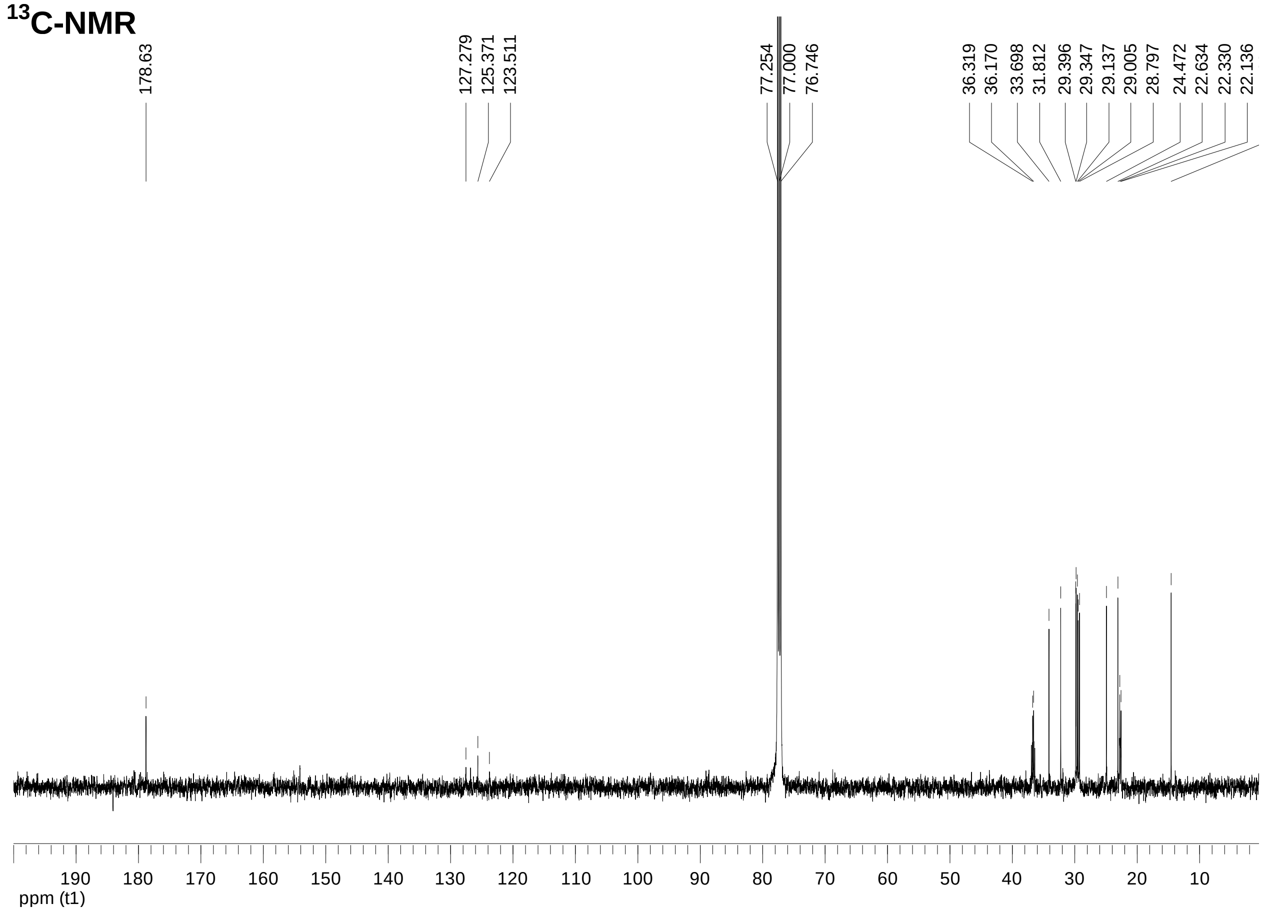

^{19}F-NMR

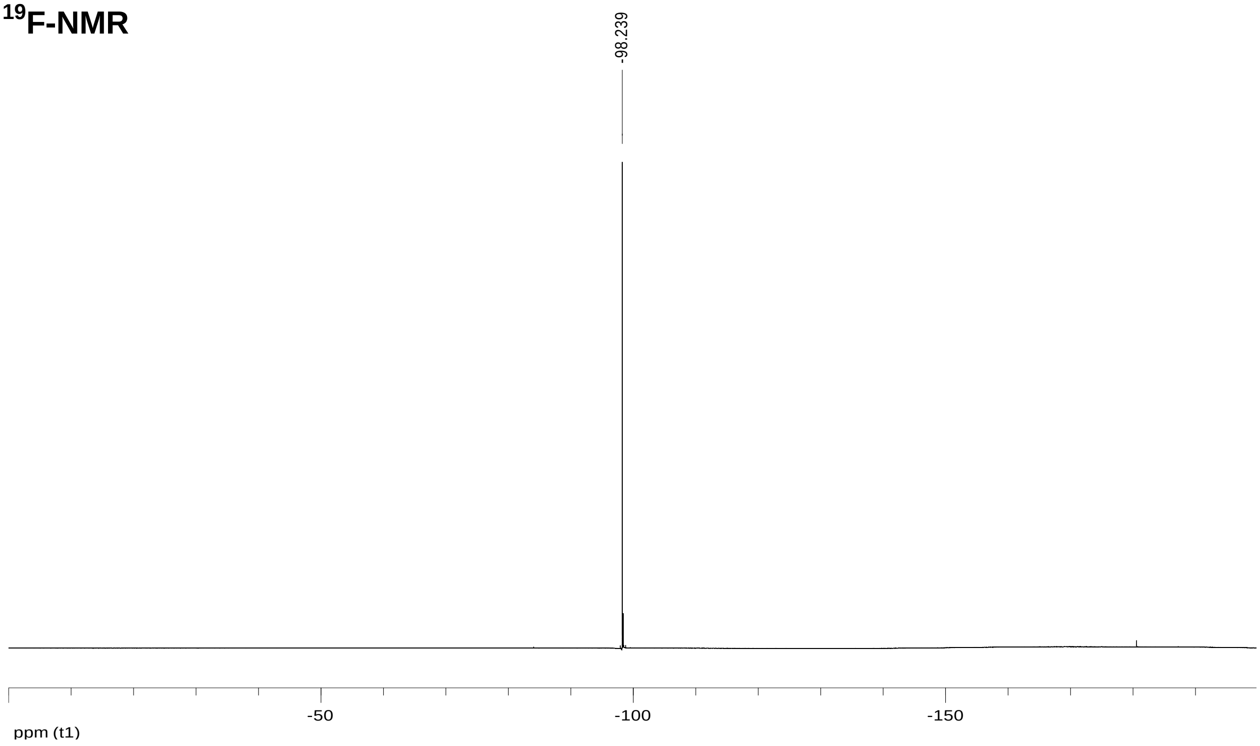

10-Phenyldec-1-en-4-ol (13).

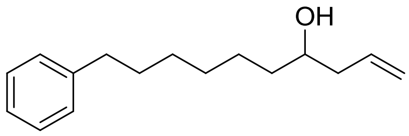

^{1}H-NMR

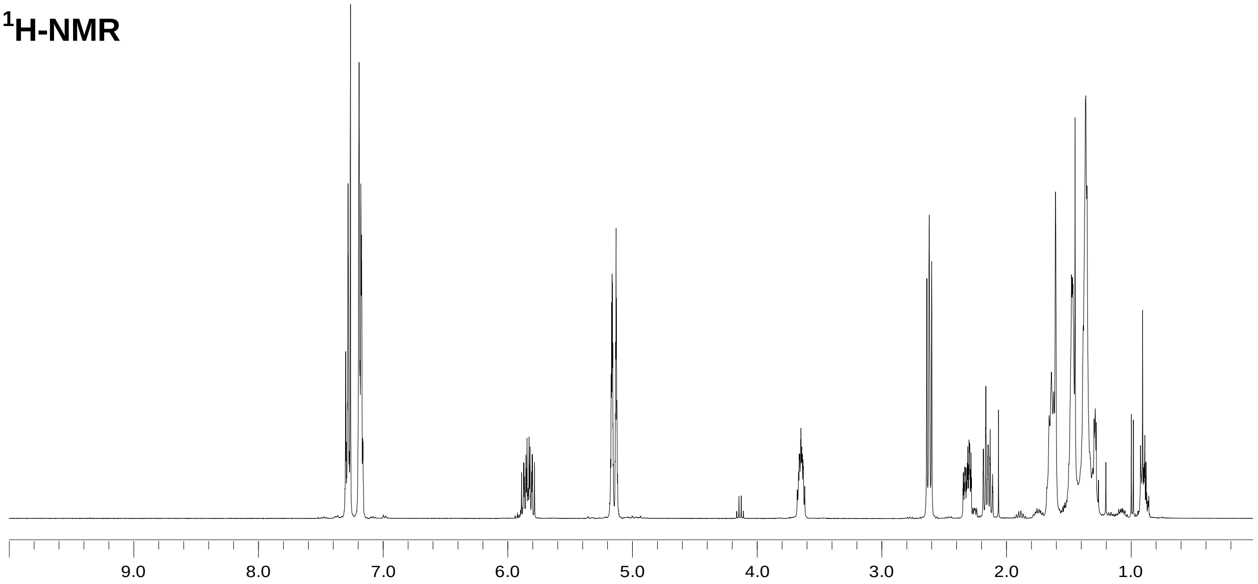

^{13}C-NMR

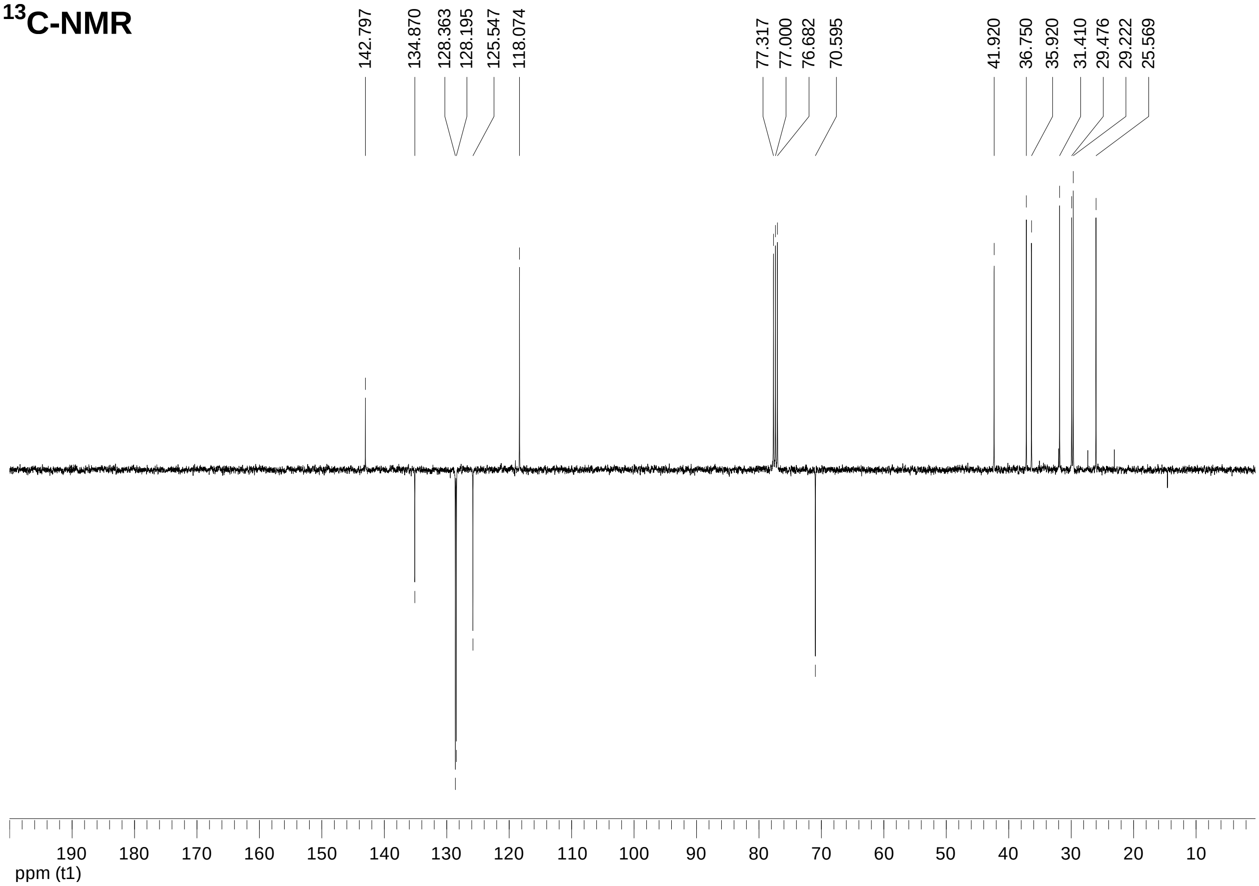

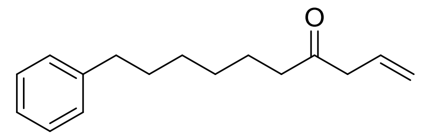

10-phenyldec-1-en-4-one (14).

^{1}H-NMR

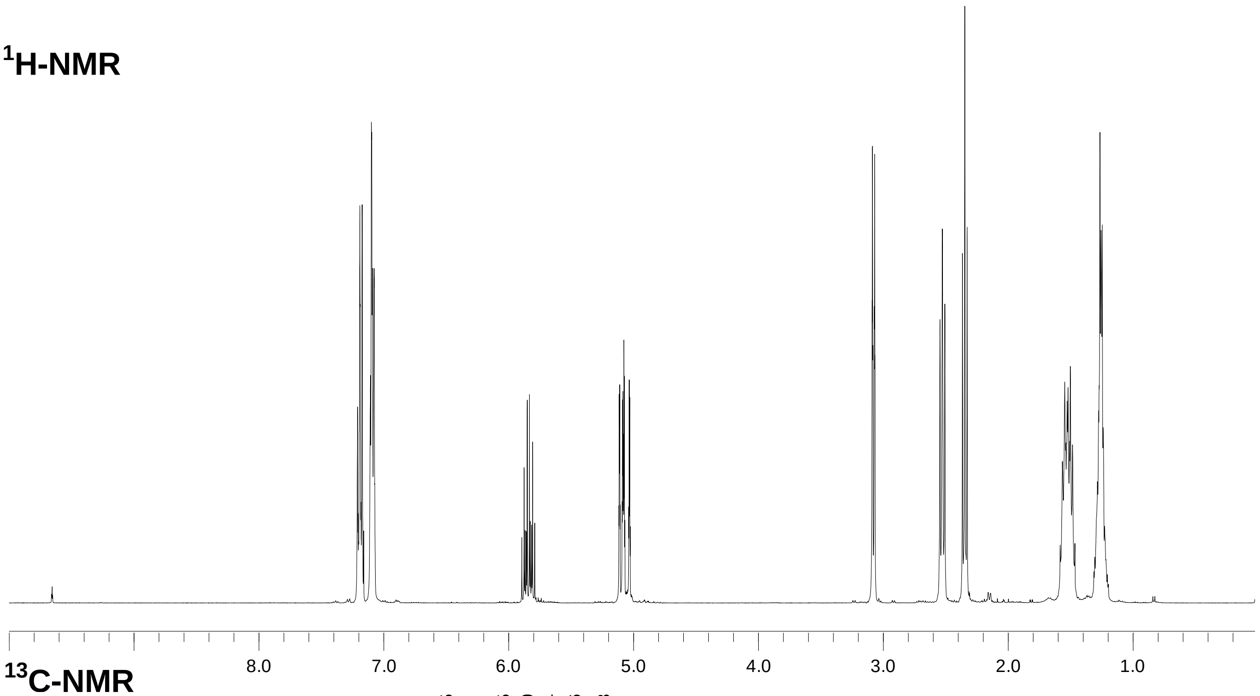

^{13}C-NMR

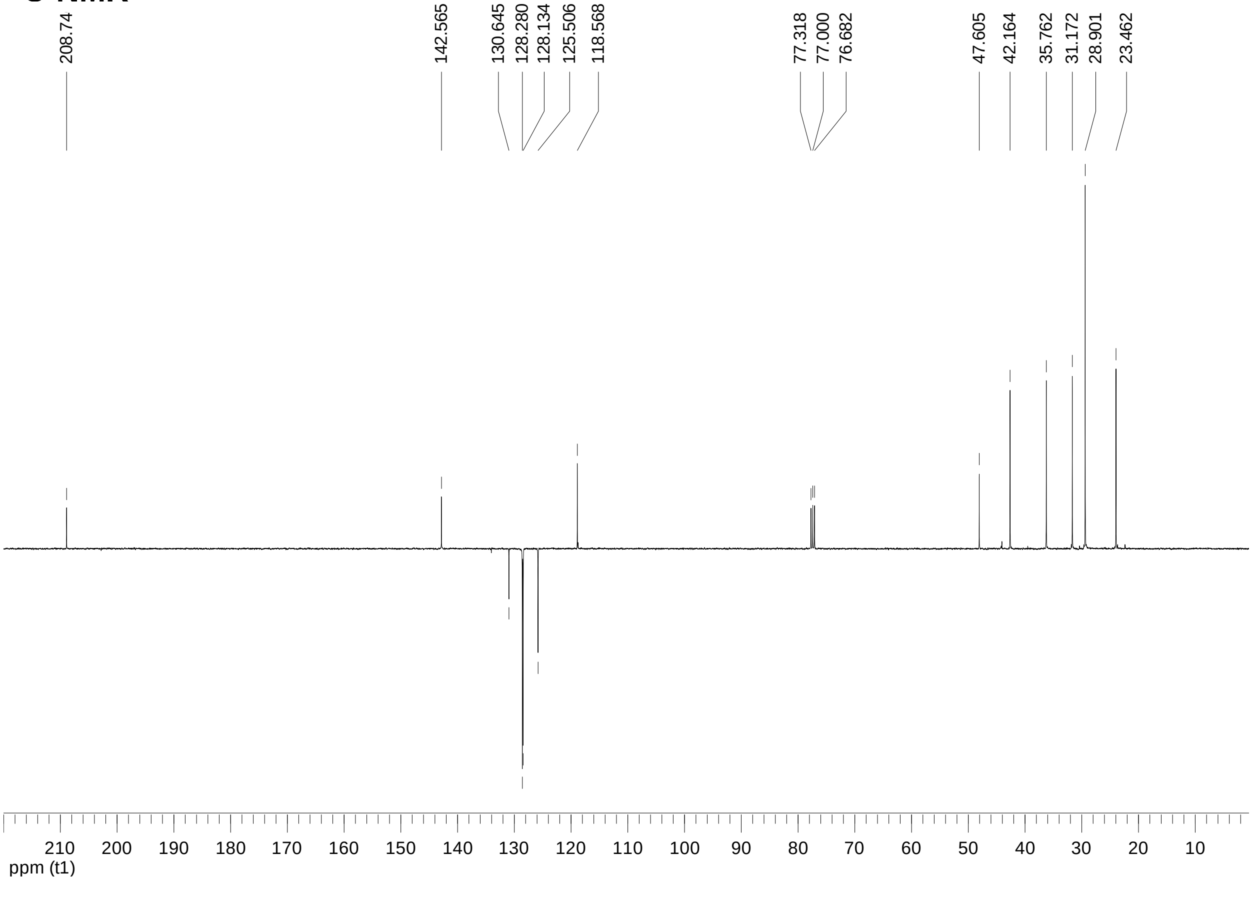

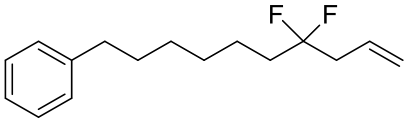

(7,7-difluorodec-9-en-1-yl)benzene (15).

^{1}H-NMR

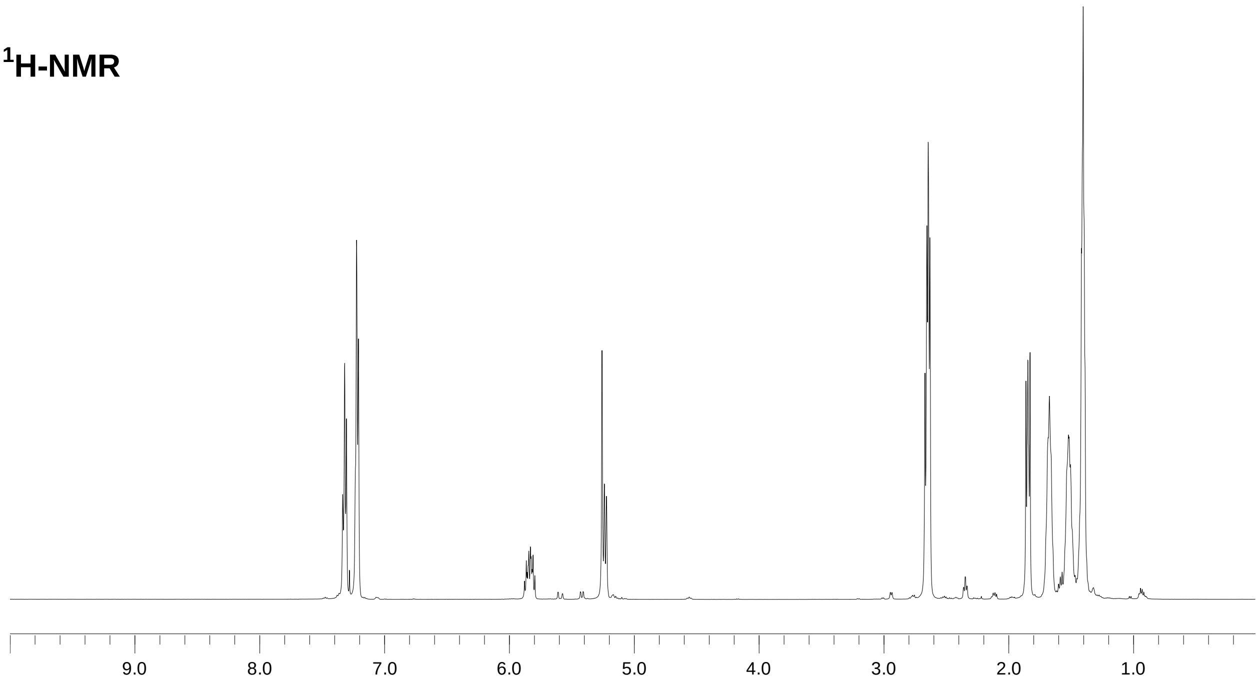

^{13}C-NMR

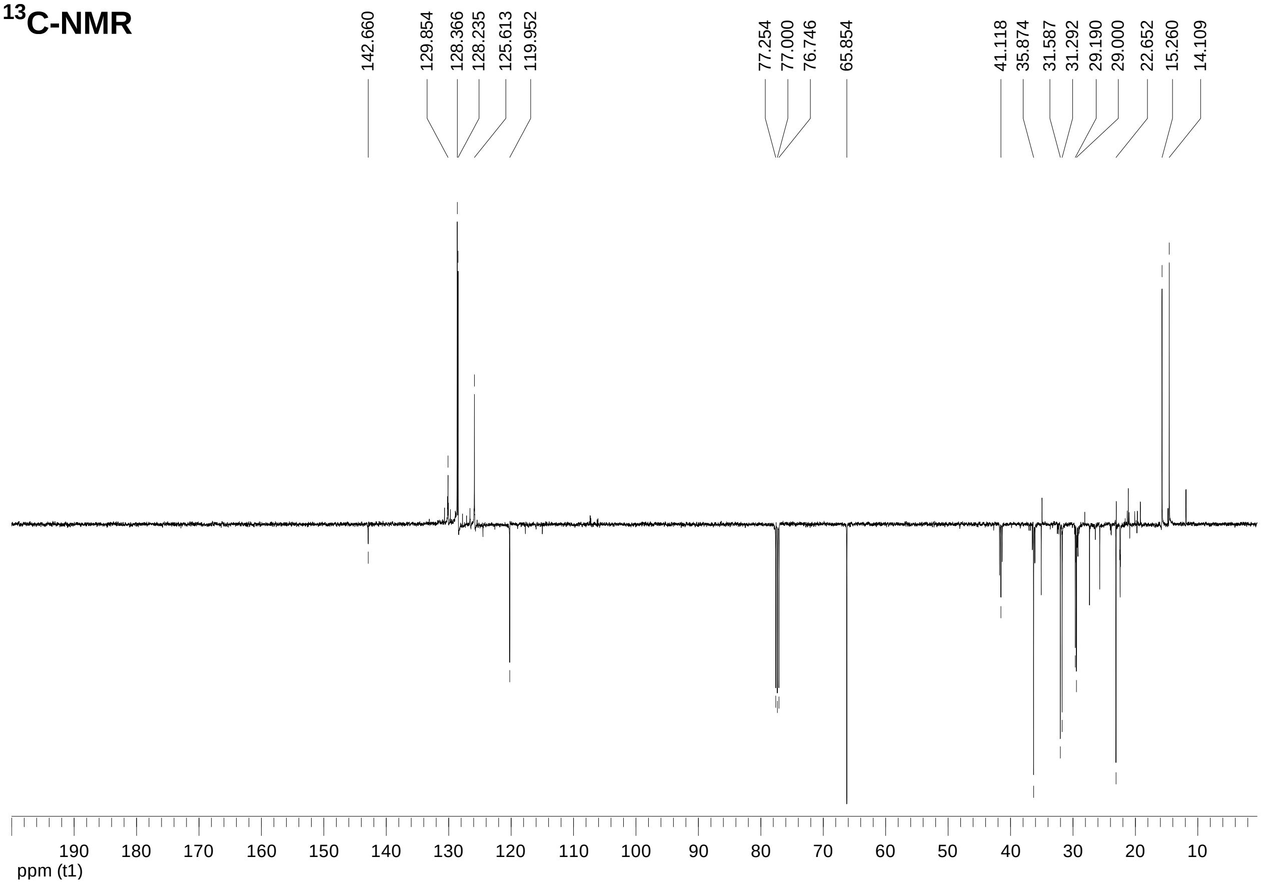

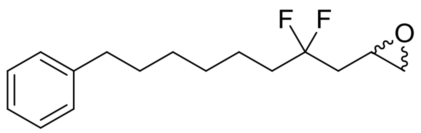

2-(2,2-difluoro-8-phenyloctyl)oxirane (16).

^{1}H-NMR

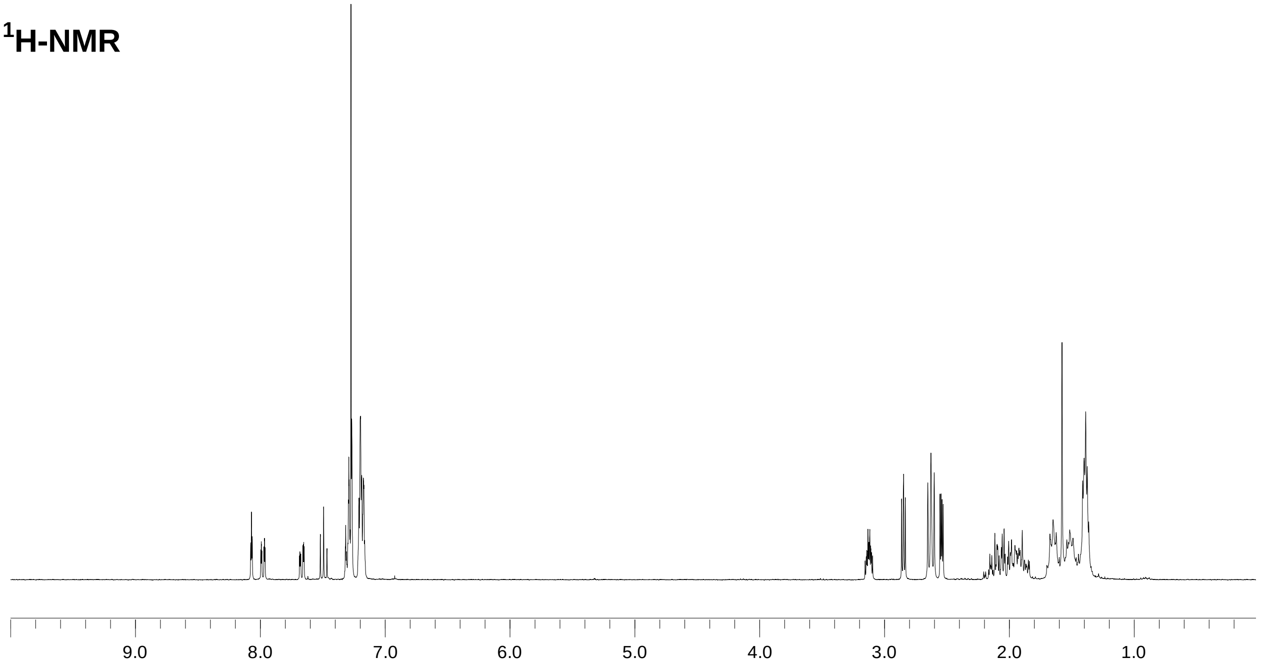

^{13}C-NMR

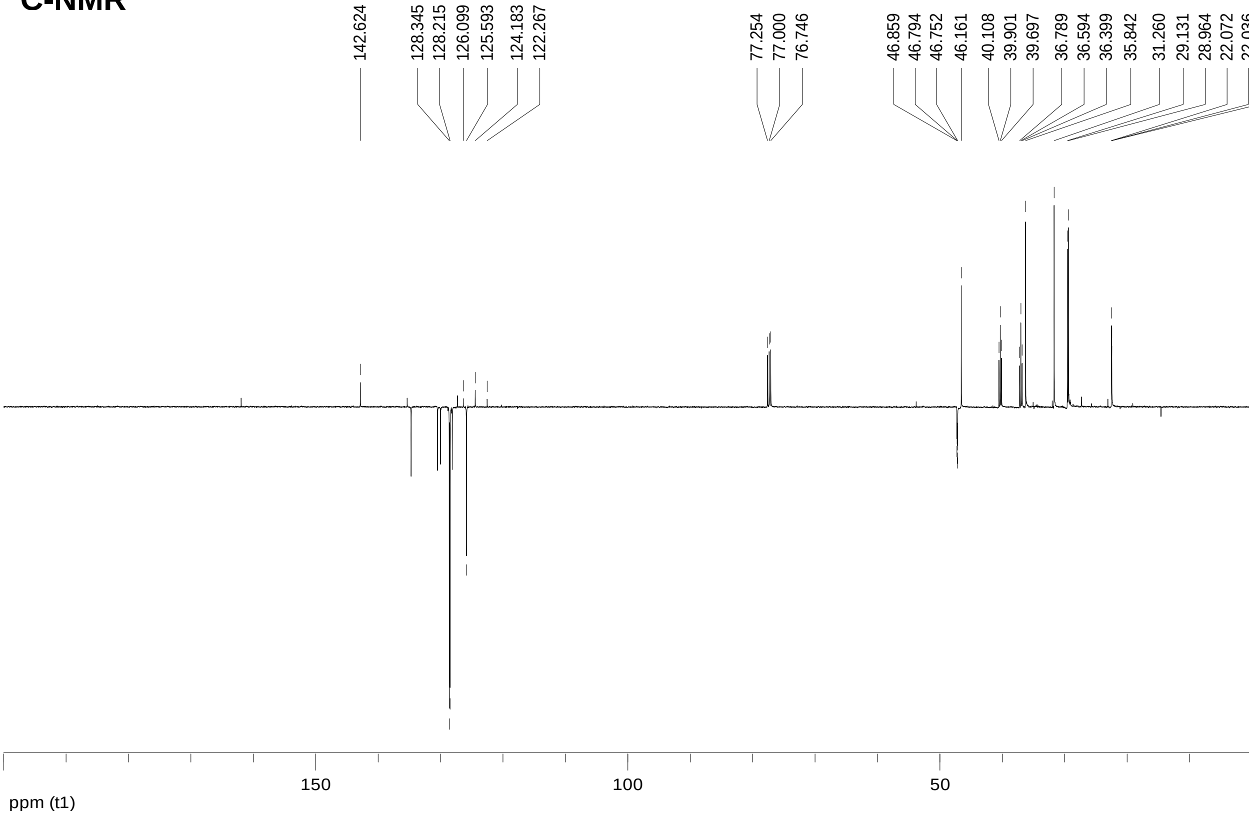

9,9-difluoro-15-phenylpentadecan-7-ol (17).

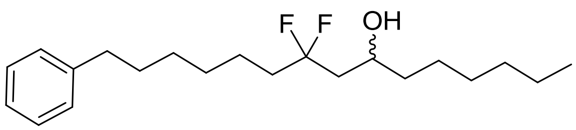

^{1}H-NMR

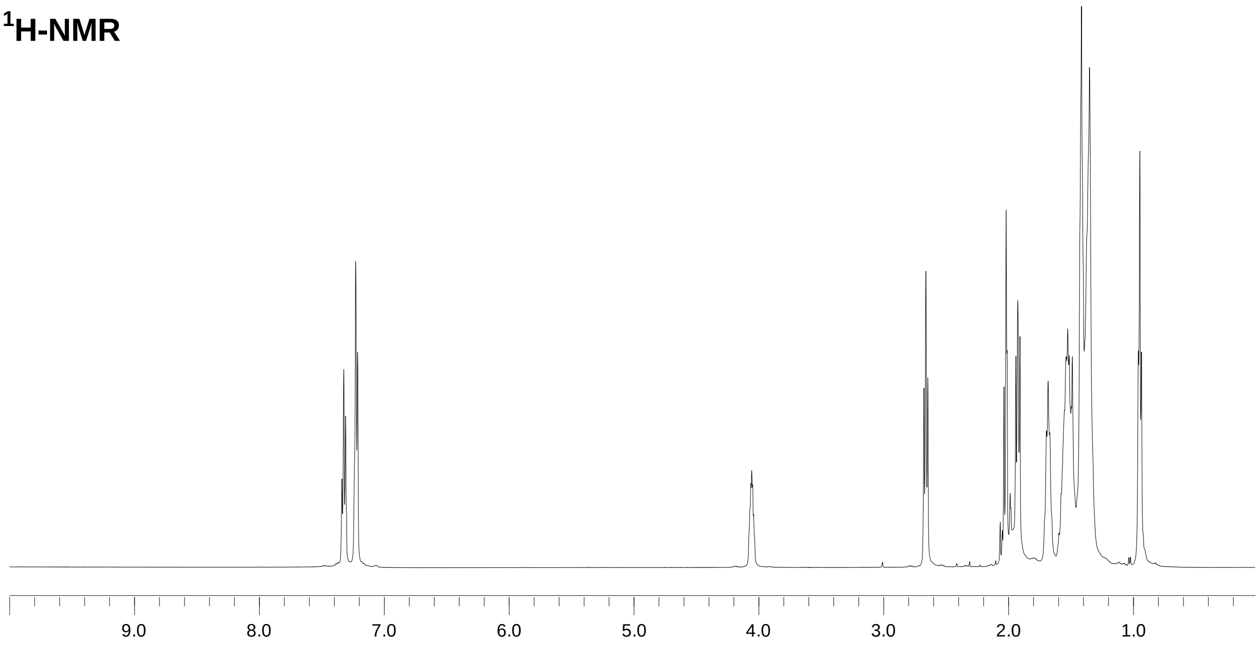

^{13}C-NMR

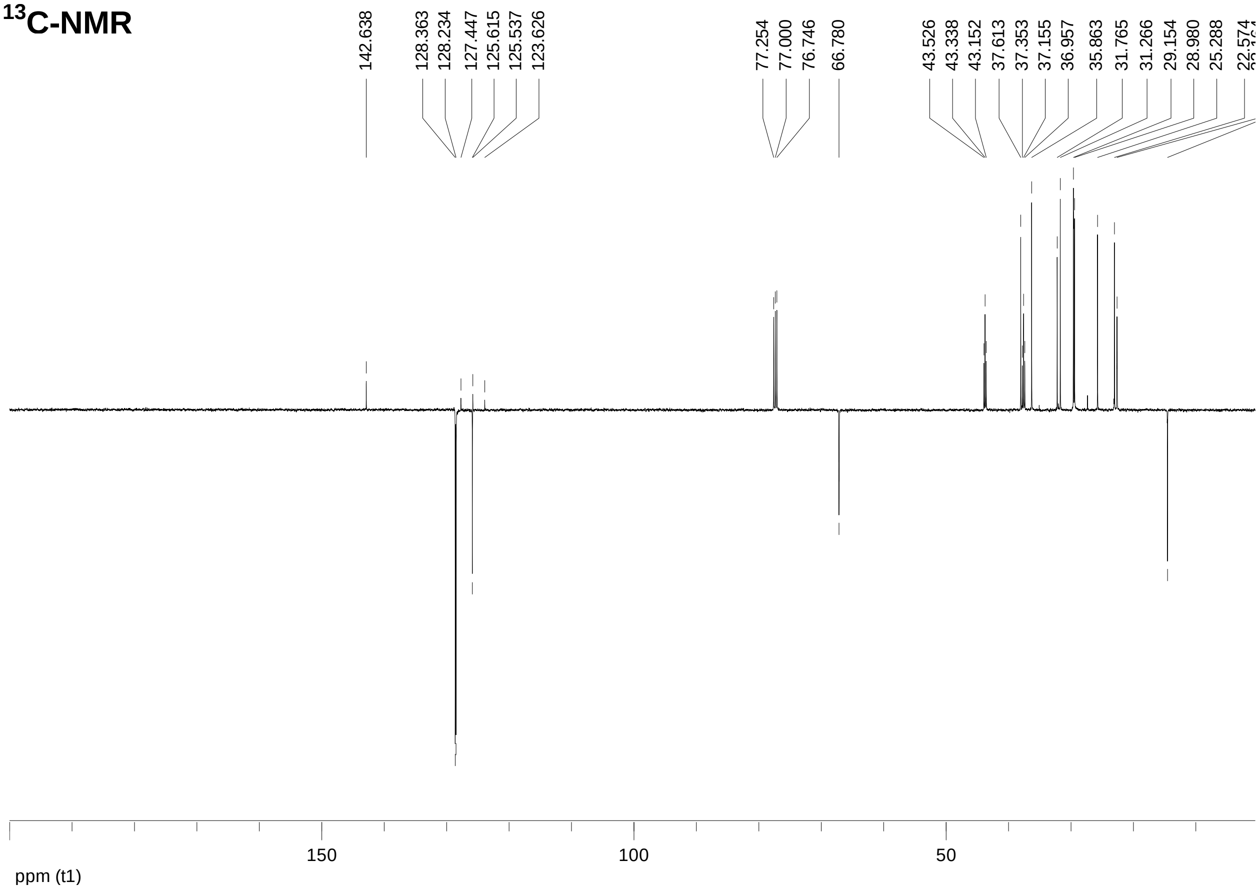

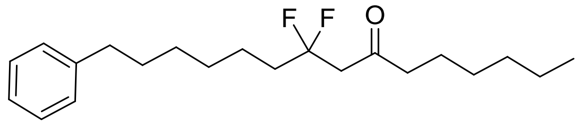

9,9-difluoro-15-phenylpentadecan-7-one (18).

^{1}H-NMR

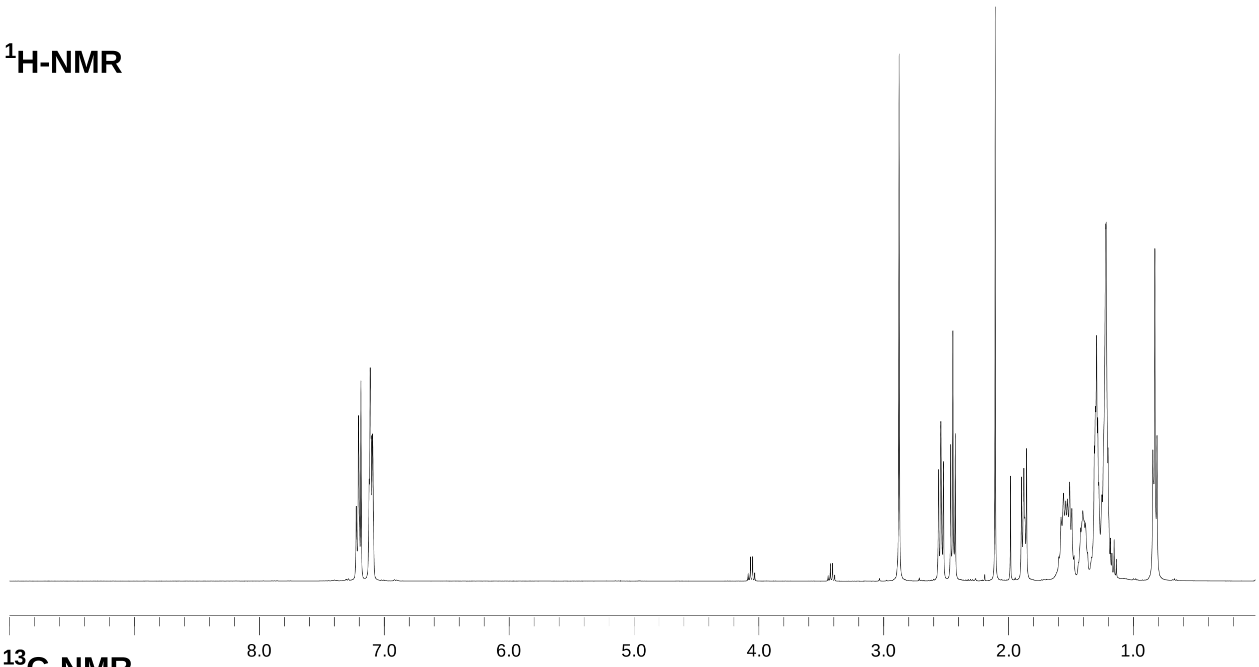

^{13}C-NMR

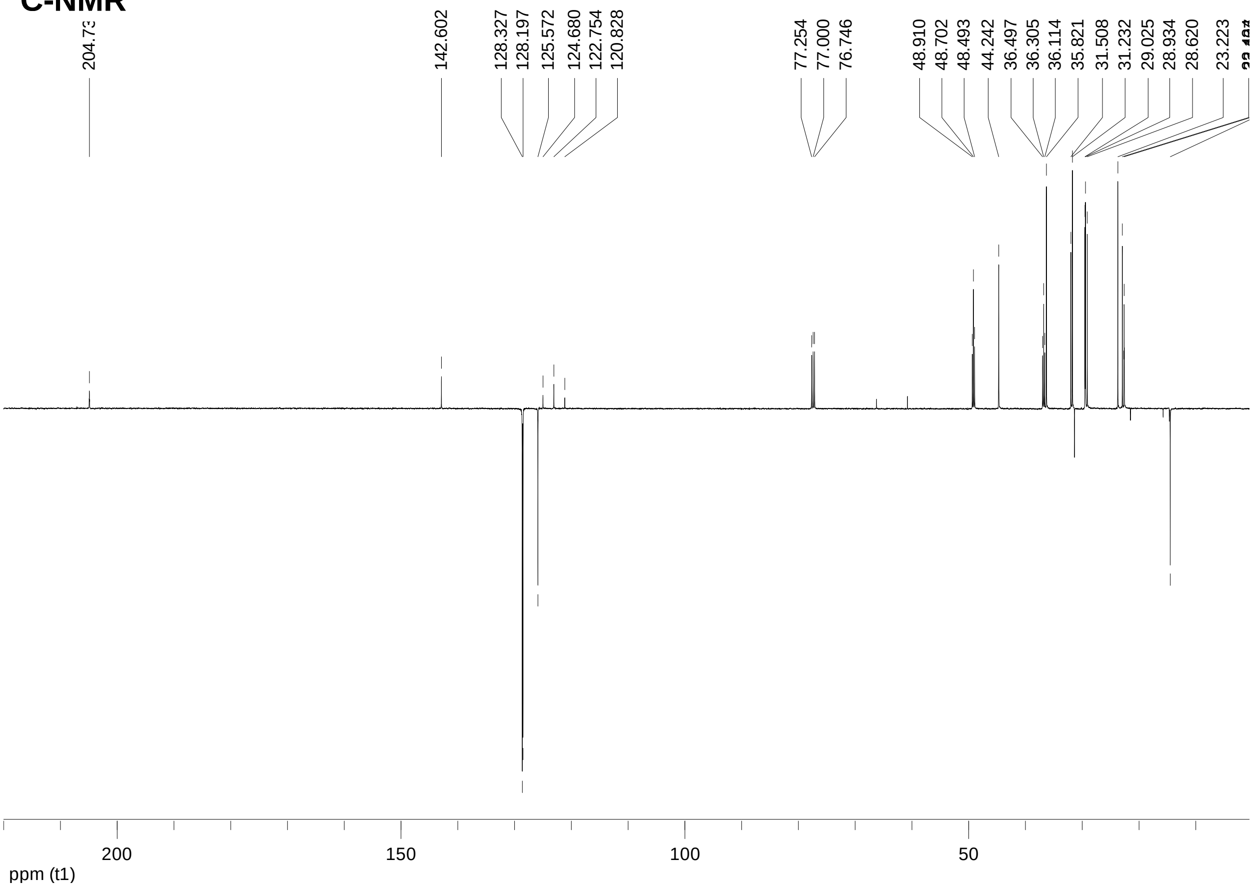

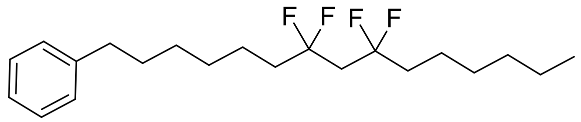

(7,7,9,9-tetrafluoropentadecyl)benzene (19).

^{1}H-NMR

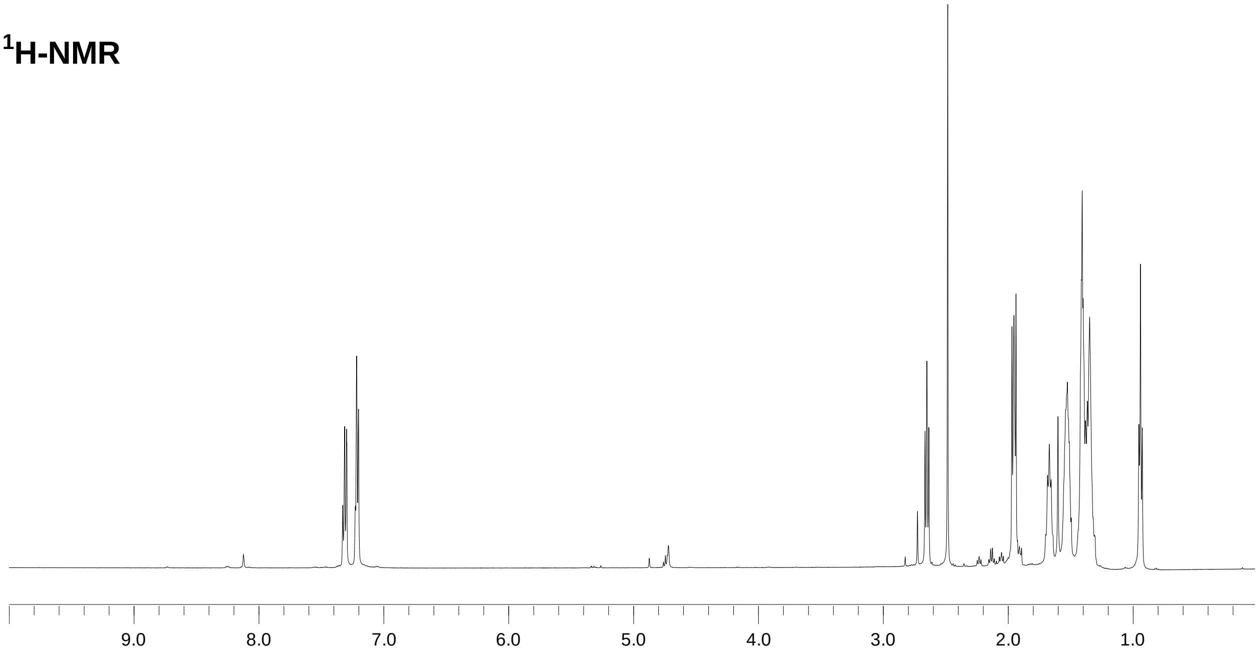

^{13}C-NMR

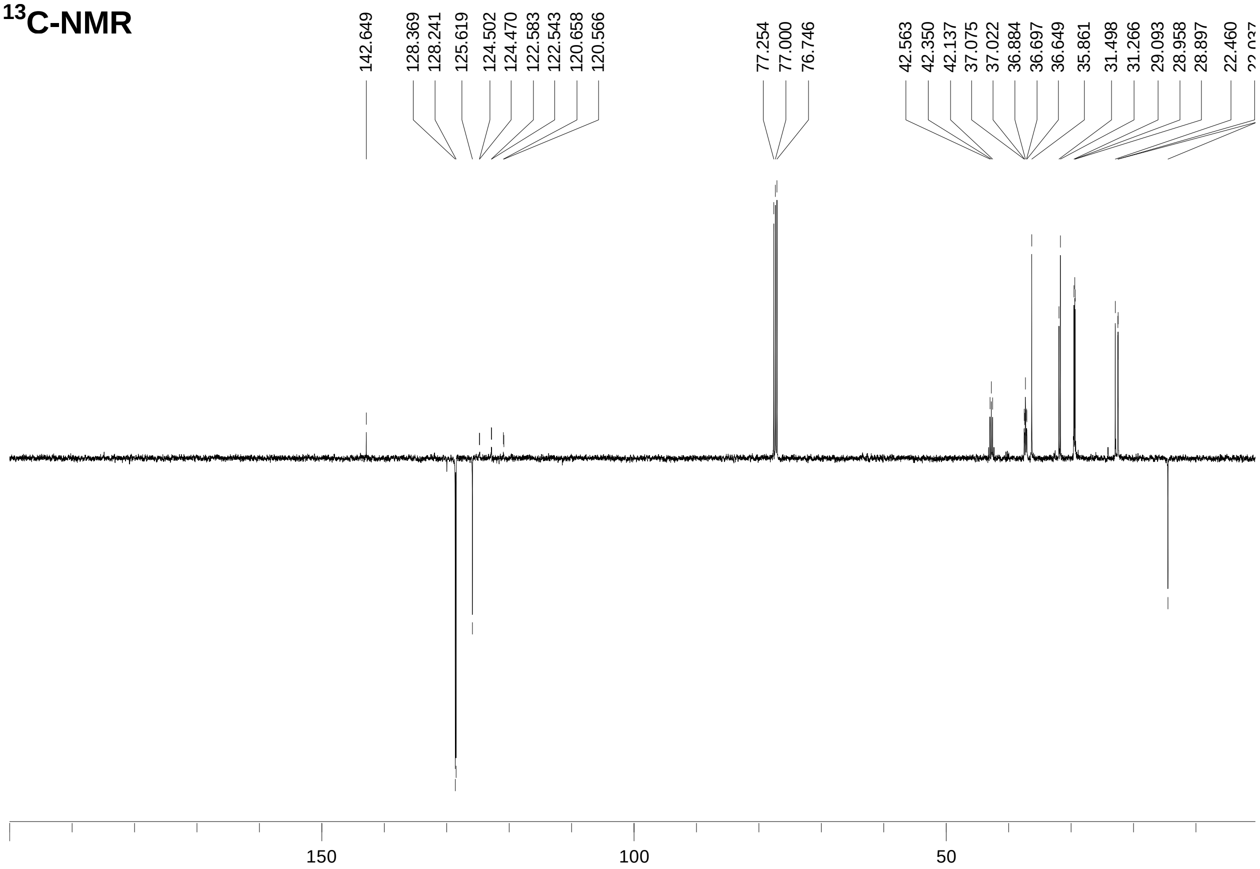

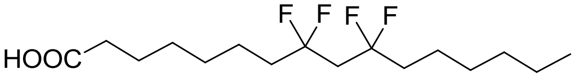

8,8,10,10-tetrafluorohexadecanoic acid (6b).

^{1}H-NMR

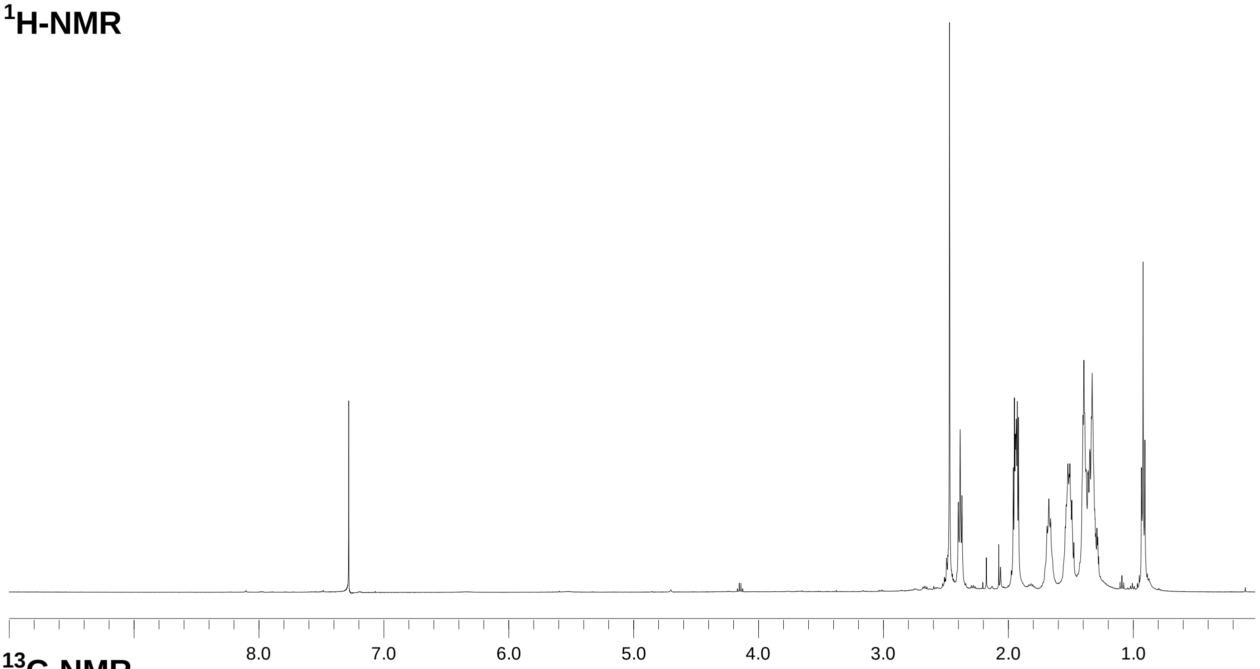

^{13}C-NMR

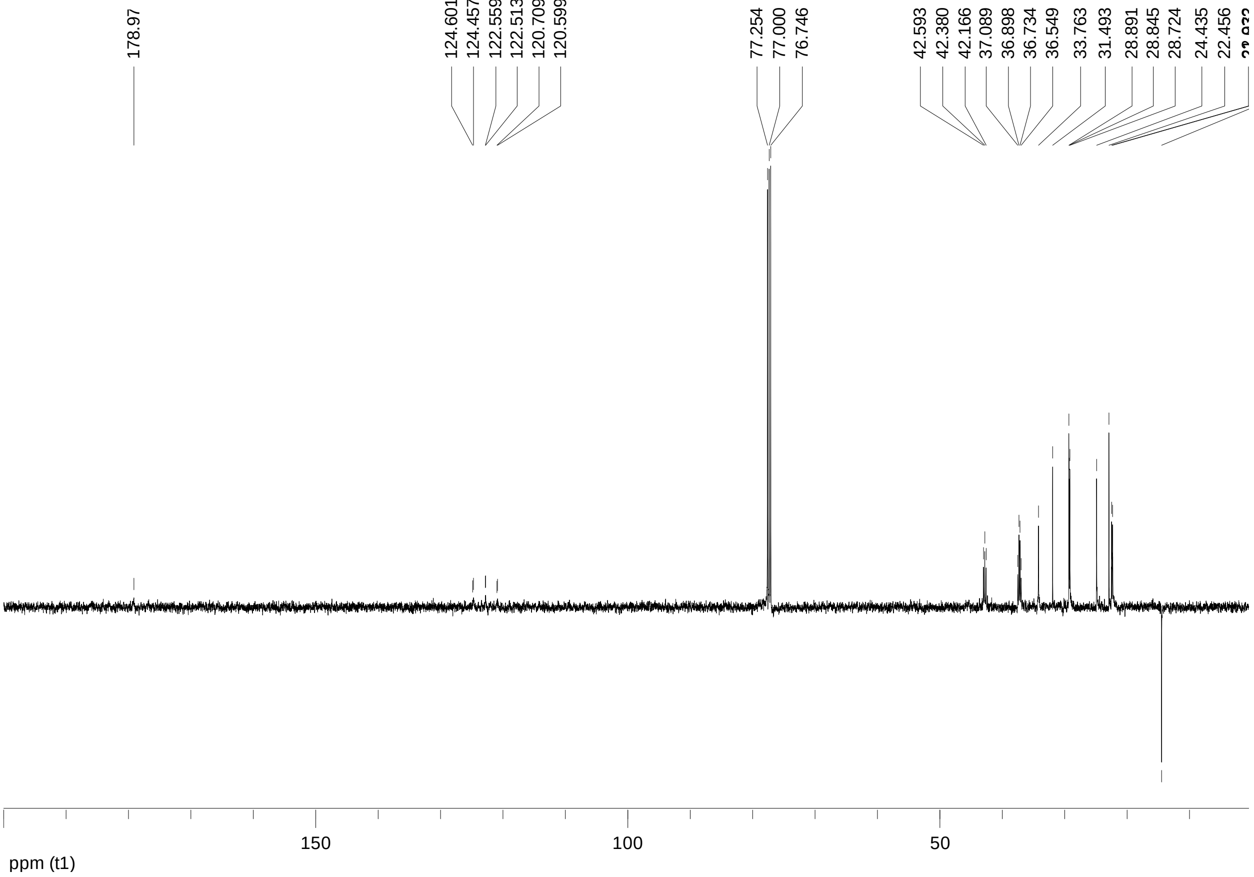

^{19}F-NMR

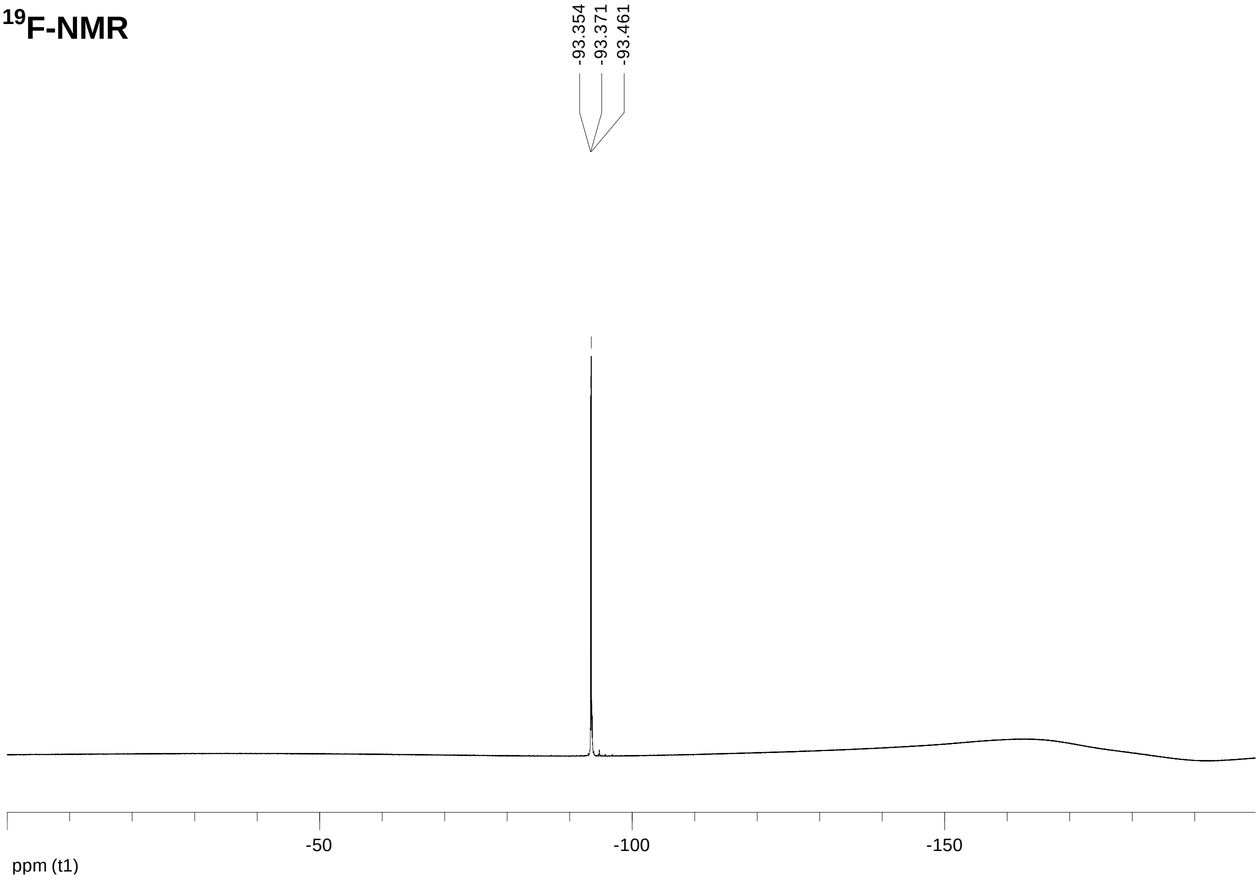

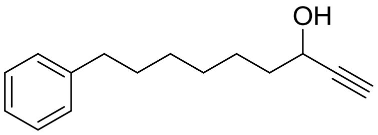

9-phenylnon-1-yn-3-ol (20).

^{1}H-NMR

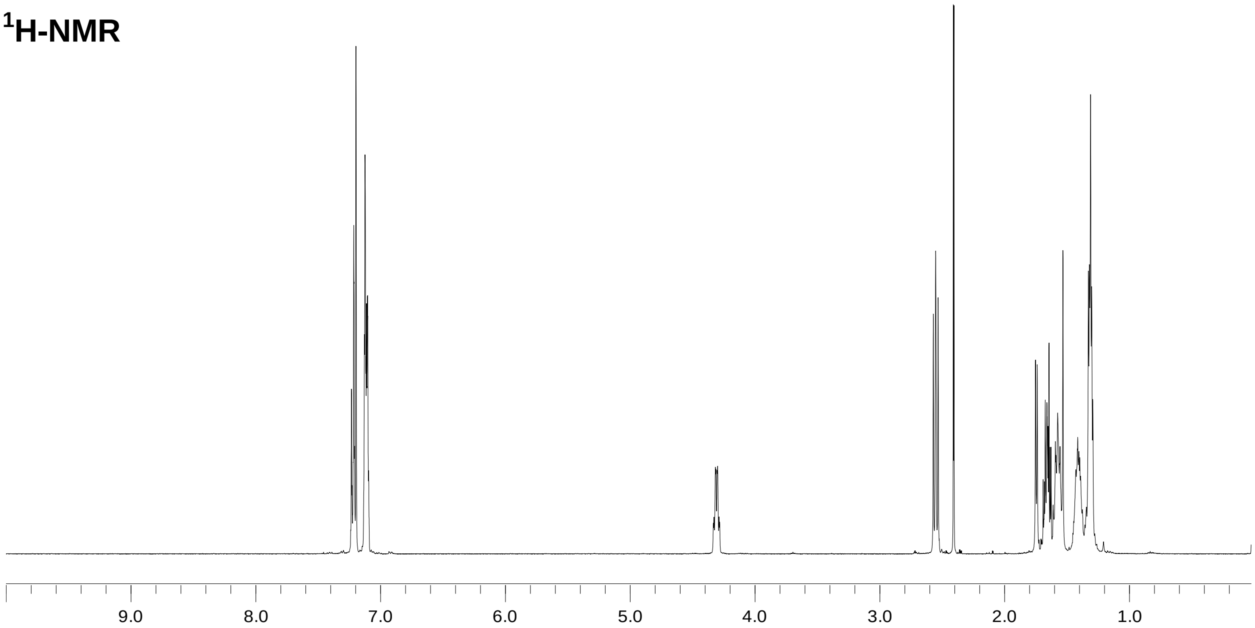

^{13}C-NMR

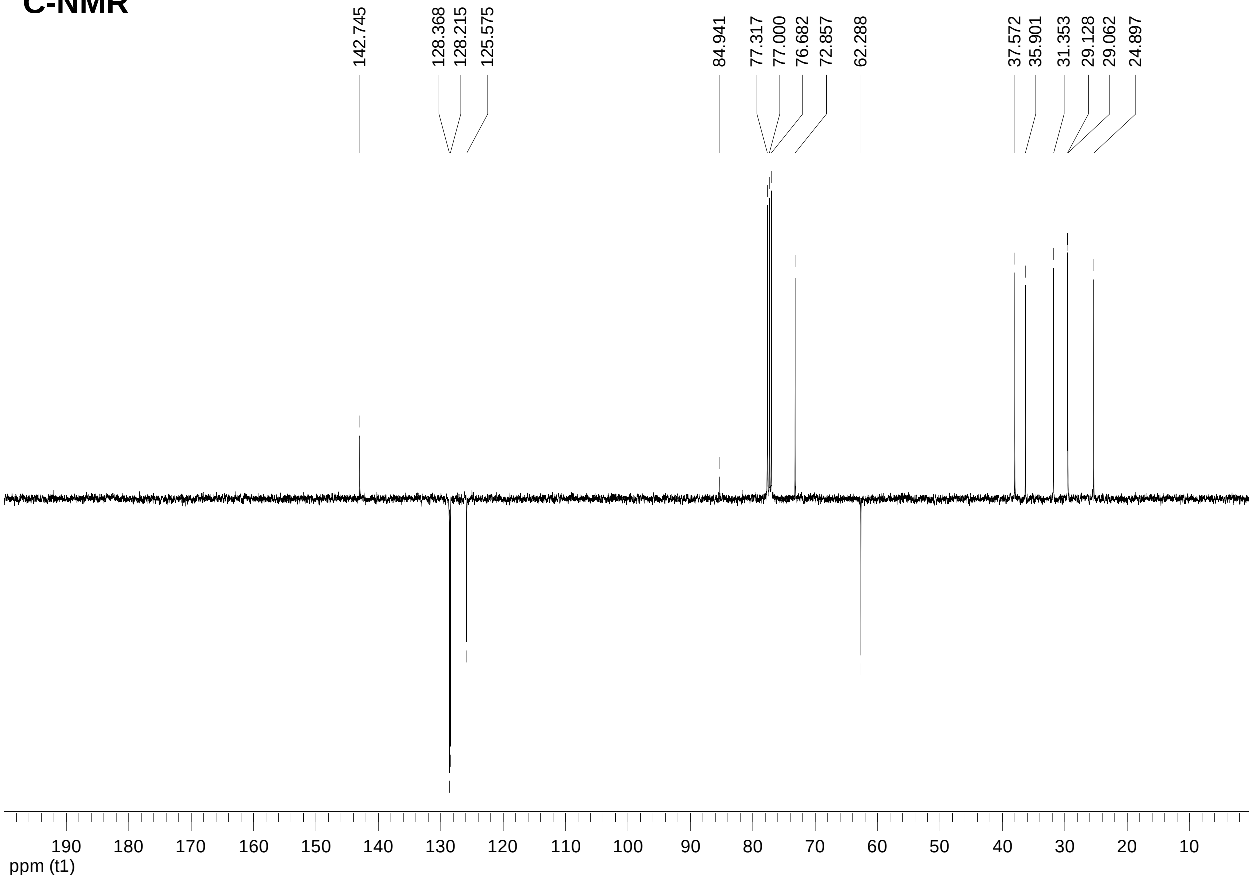

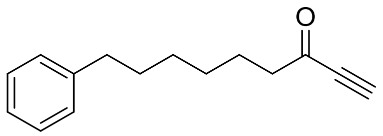

9-phenylnon-1-yn-3-one (21).

^{1}H-NMR

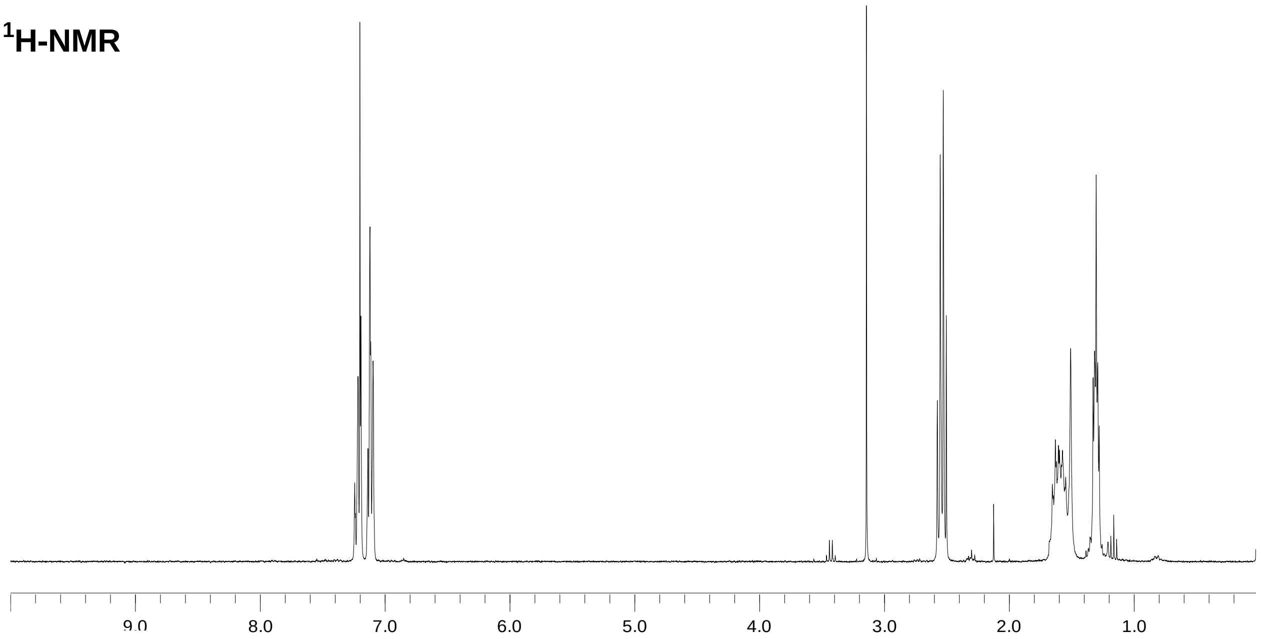

^{13}C-NMR

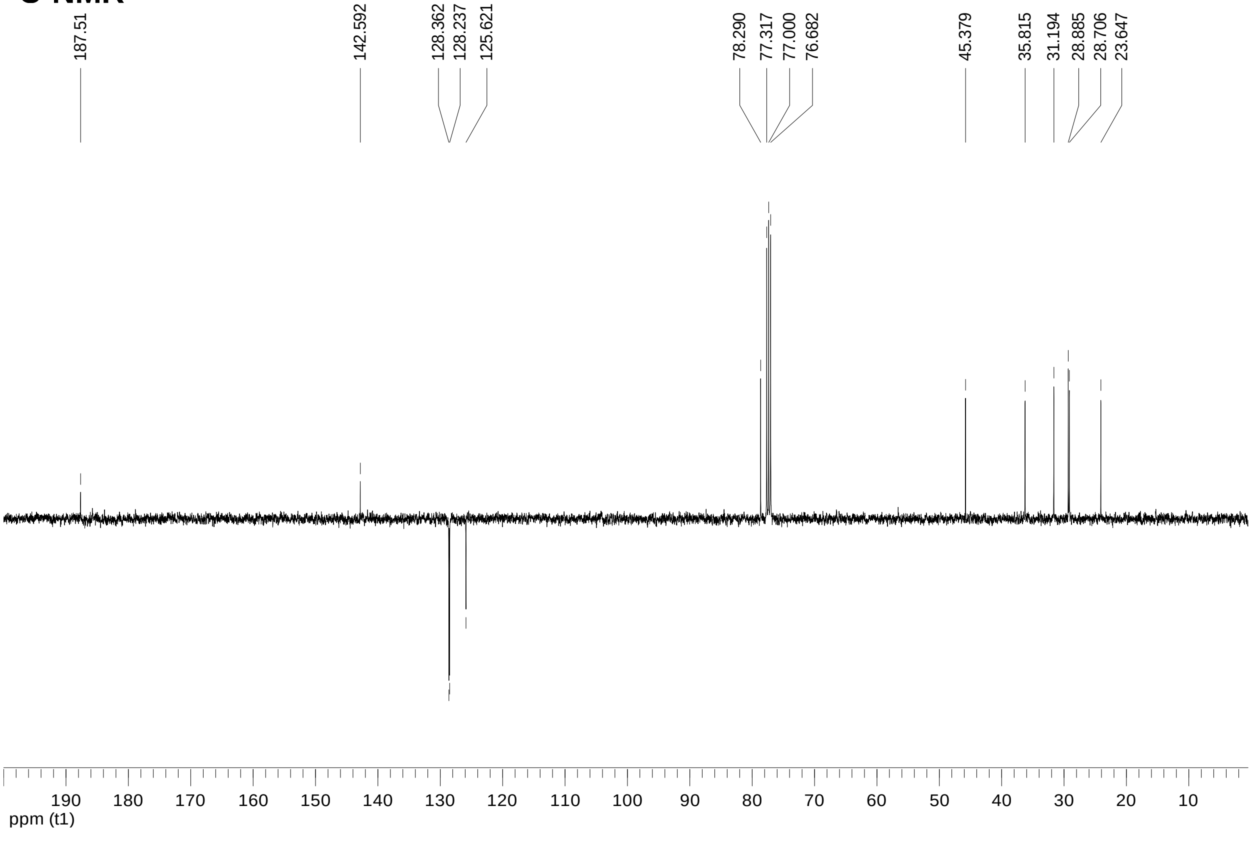

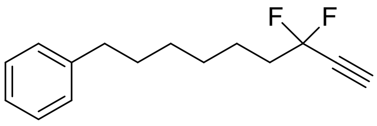

(7,7-difluoronon-8-yn-1-yl)benzene (22).

^{1}H-NMR

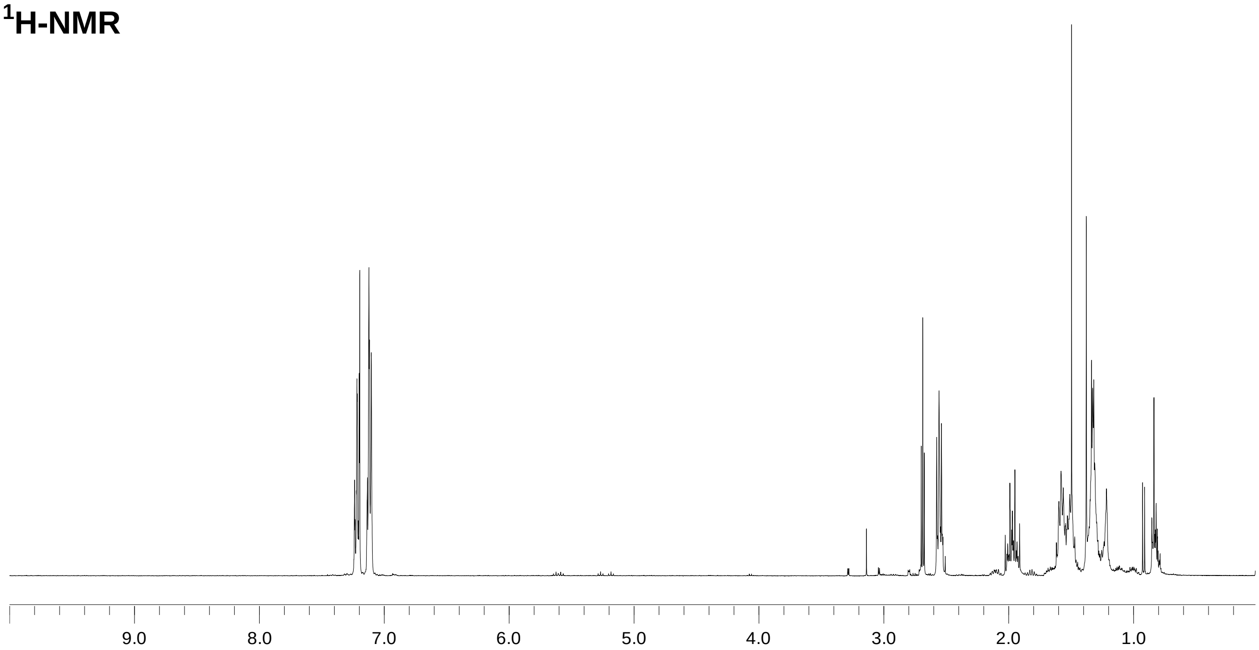

^{13}C-NMR

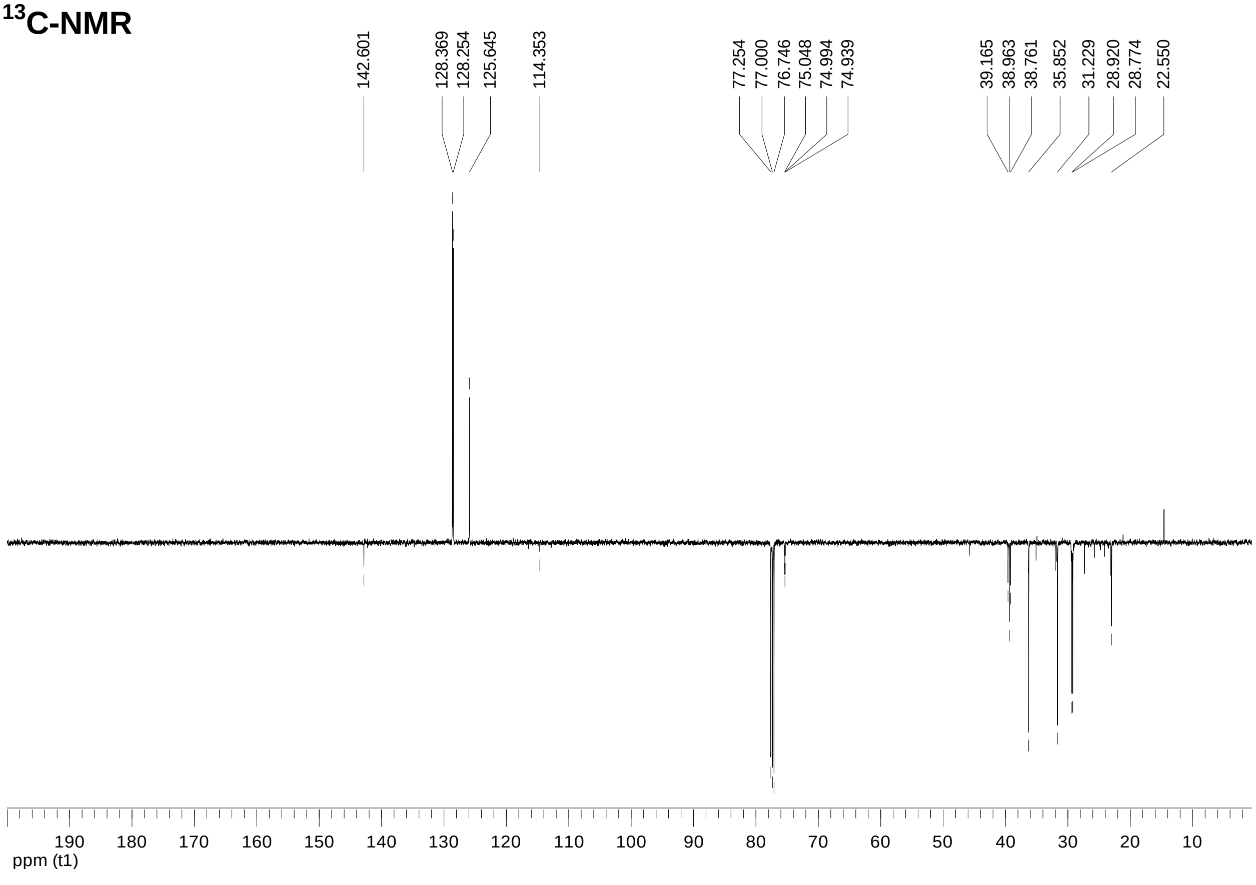

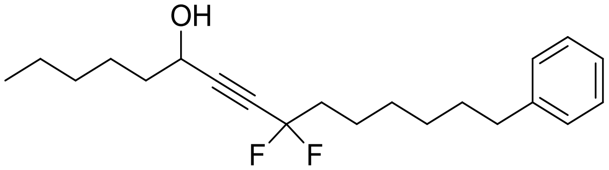

9,9-difluoro-15-phenylpentadec-7-yn-6-ol (23).

^{1}H-NMR

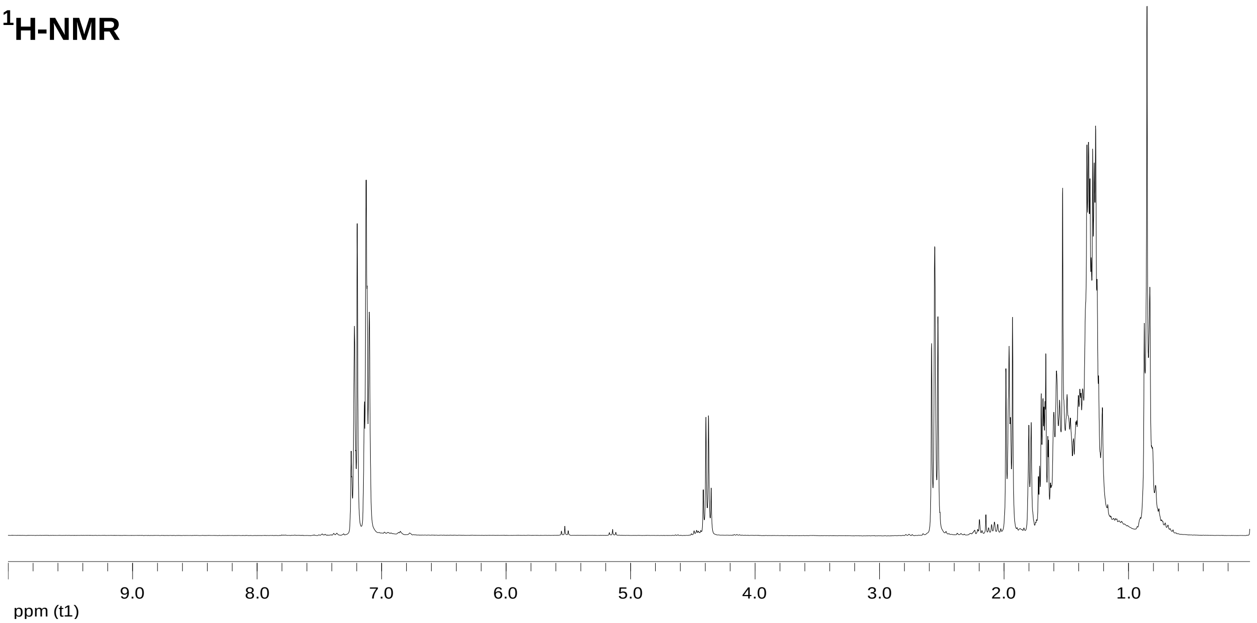

^{13}C-NMR

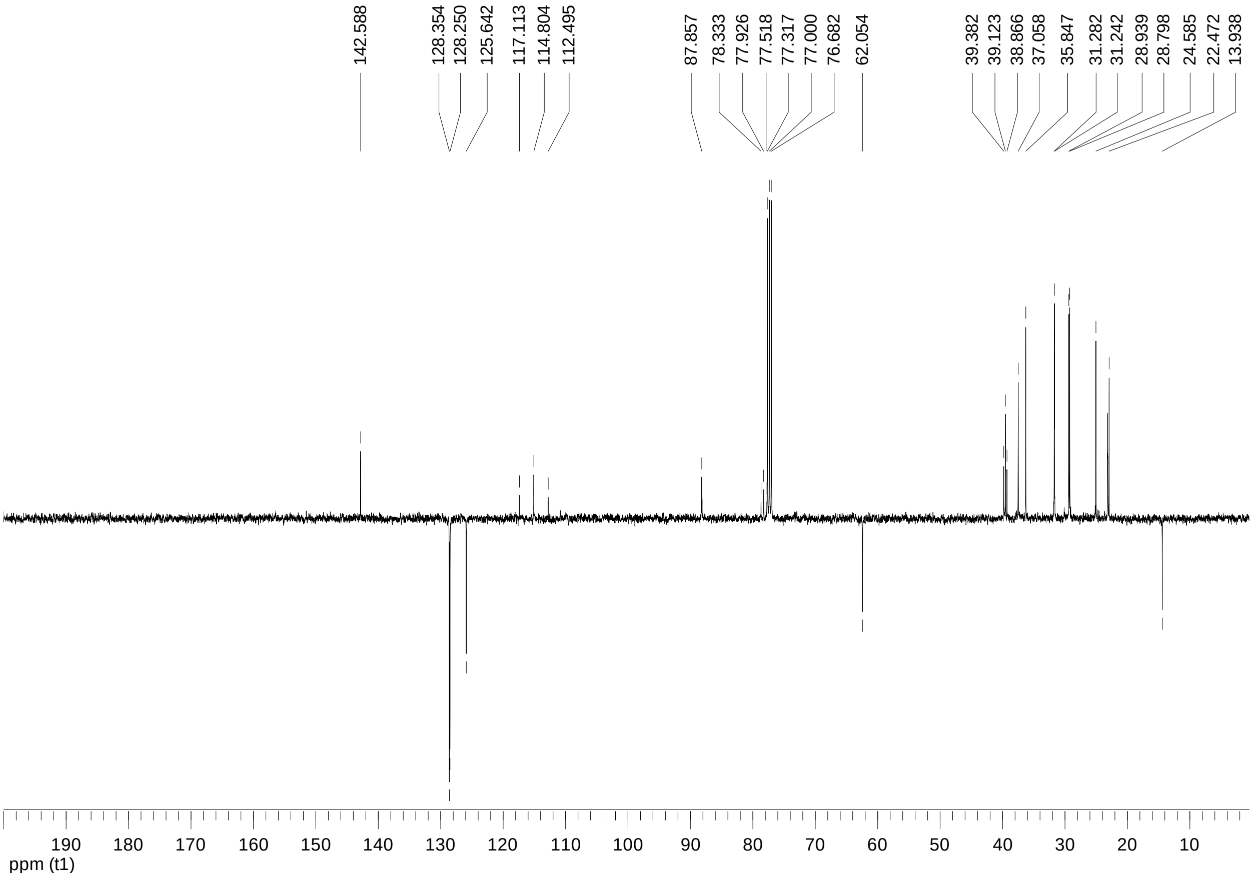

9,9-difluoro-15-phenylpentadec-7-yn-6-one (24).

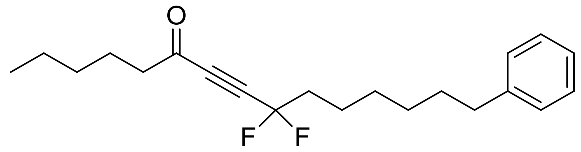

^{1}H-NMR

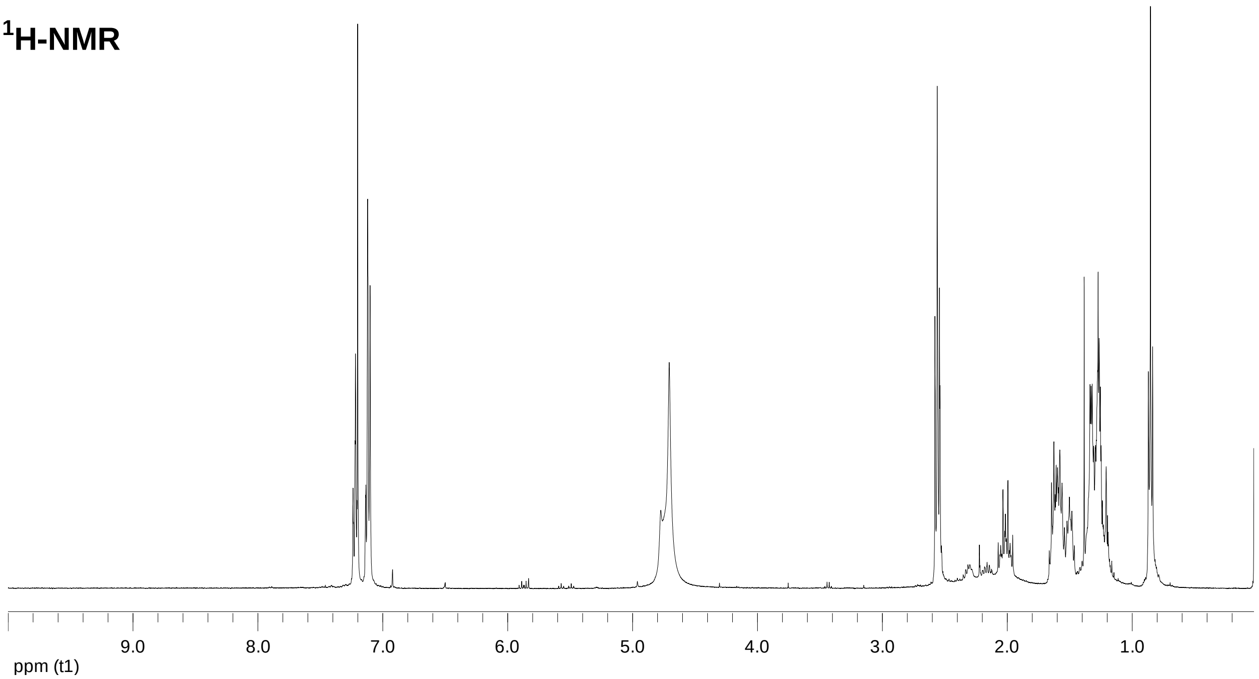

^{13}C-NMR

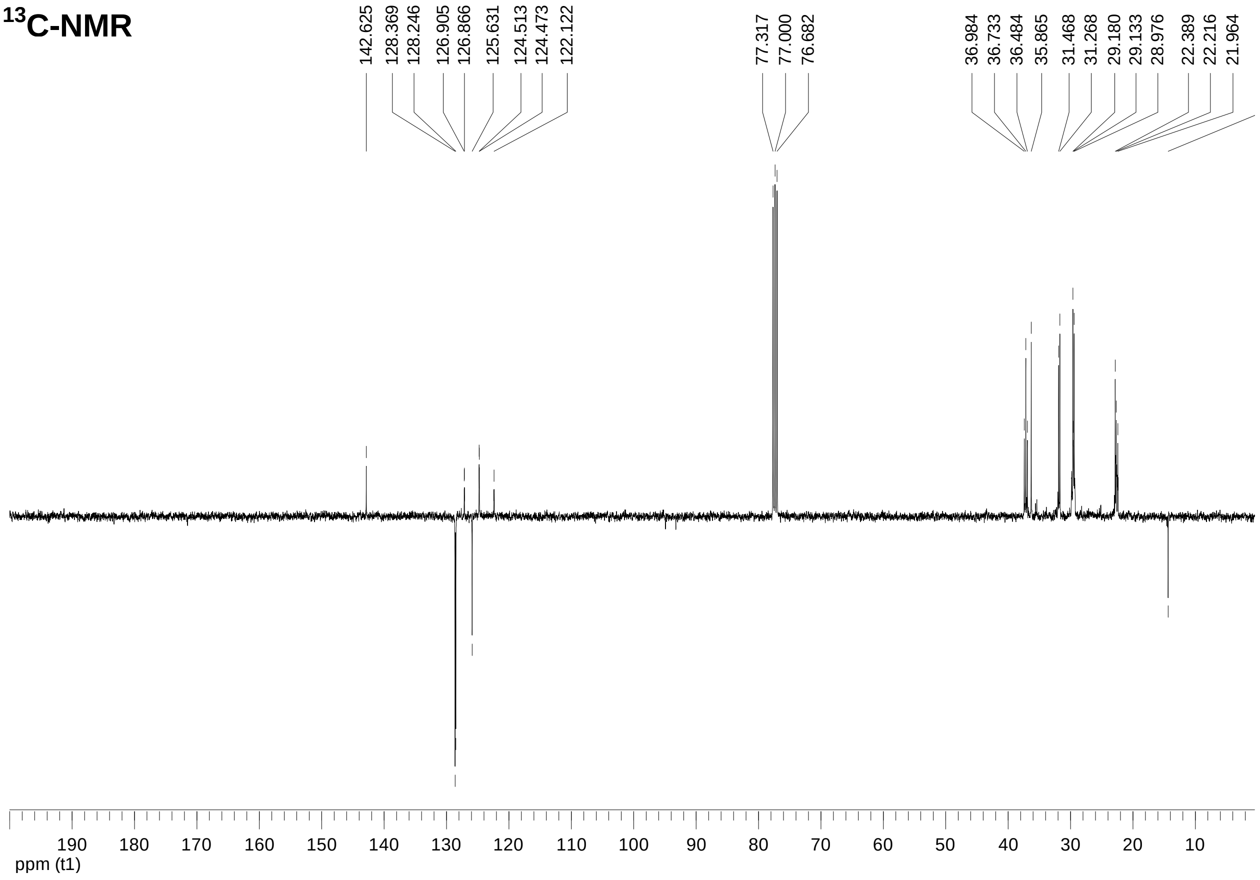

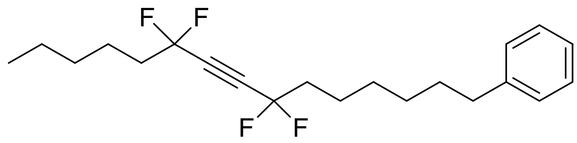

(7,7,10,10-tetrafluoropentadec-8-yn-1-yl)benzene (25).

^{1}H-NMR

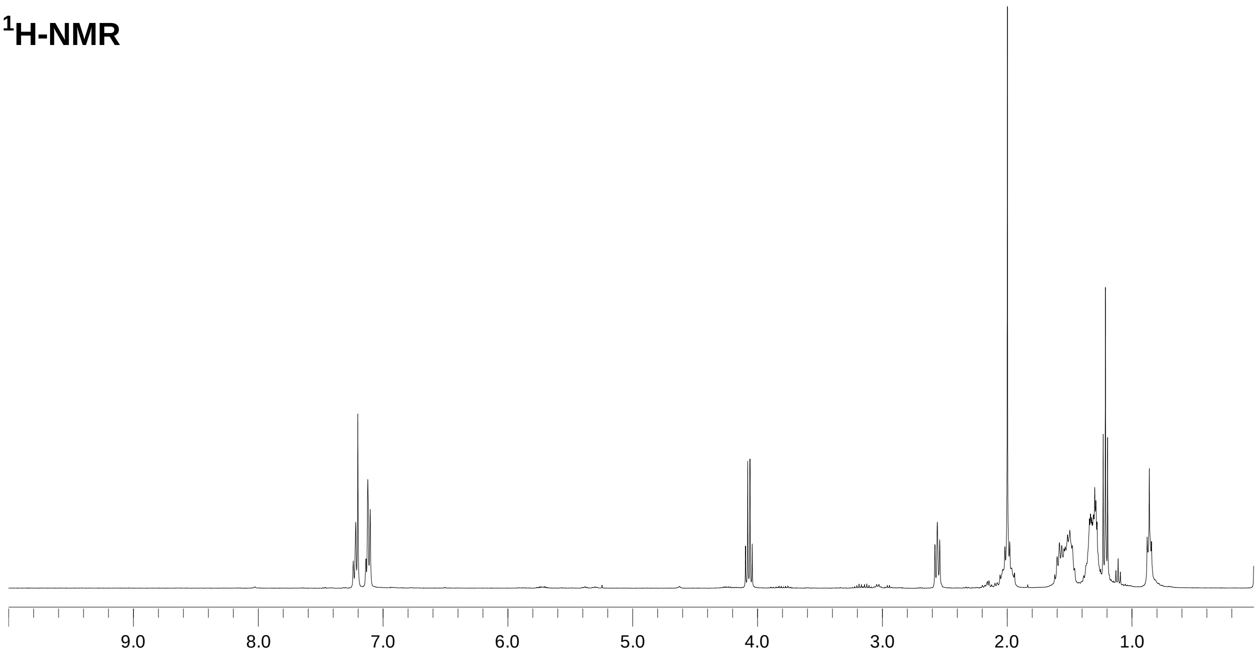

^{13}C-NMR

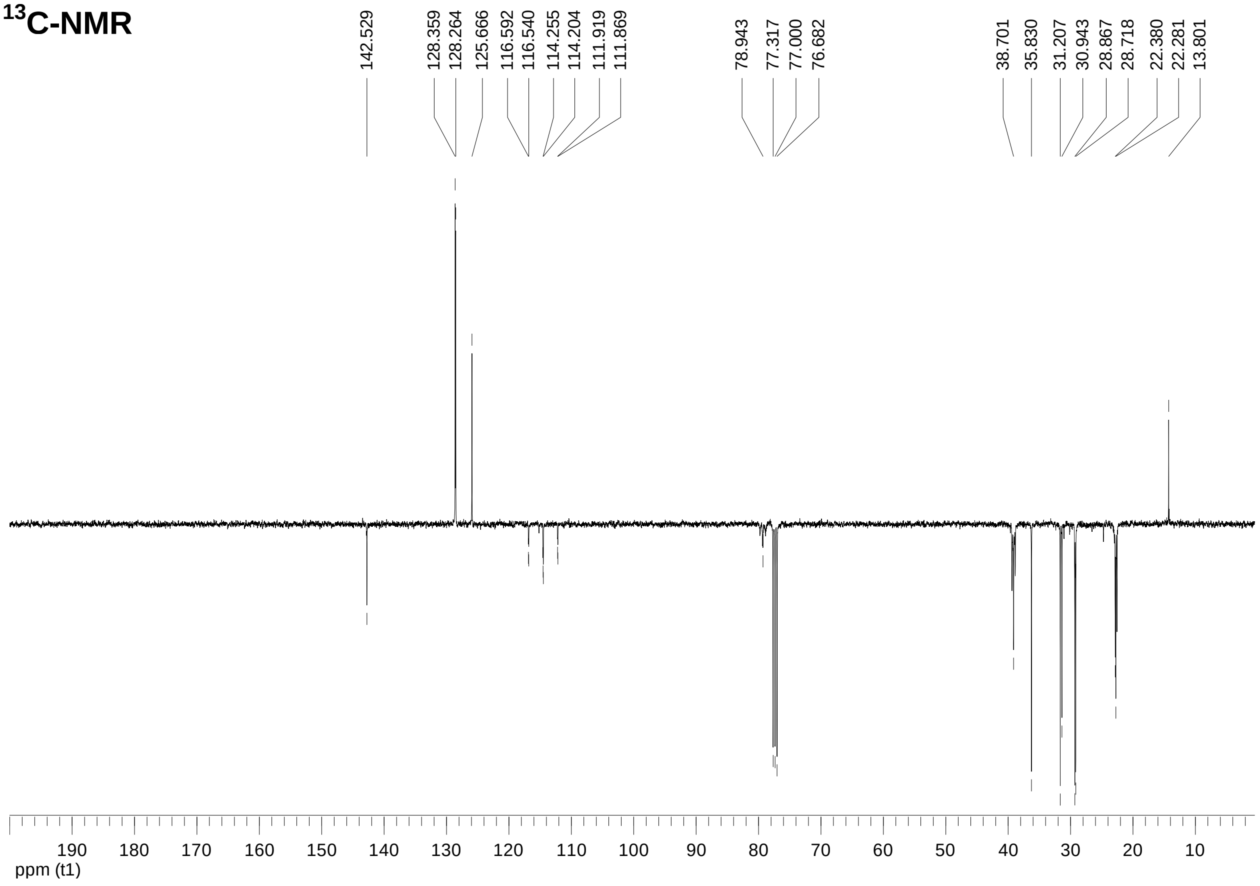

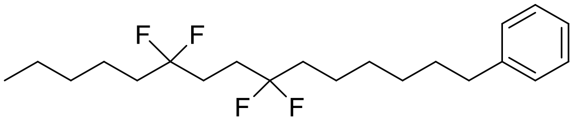

(7,7,10,10-tetrafluoropentadecyl)benzene (26).

^{1}H-NMR

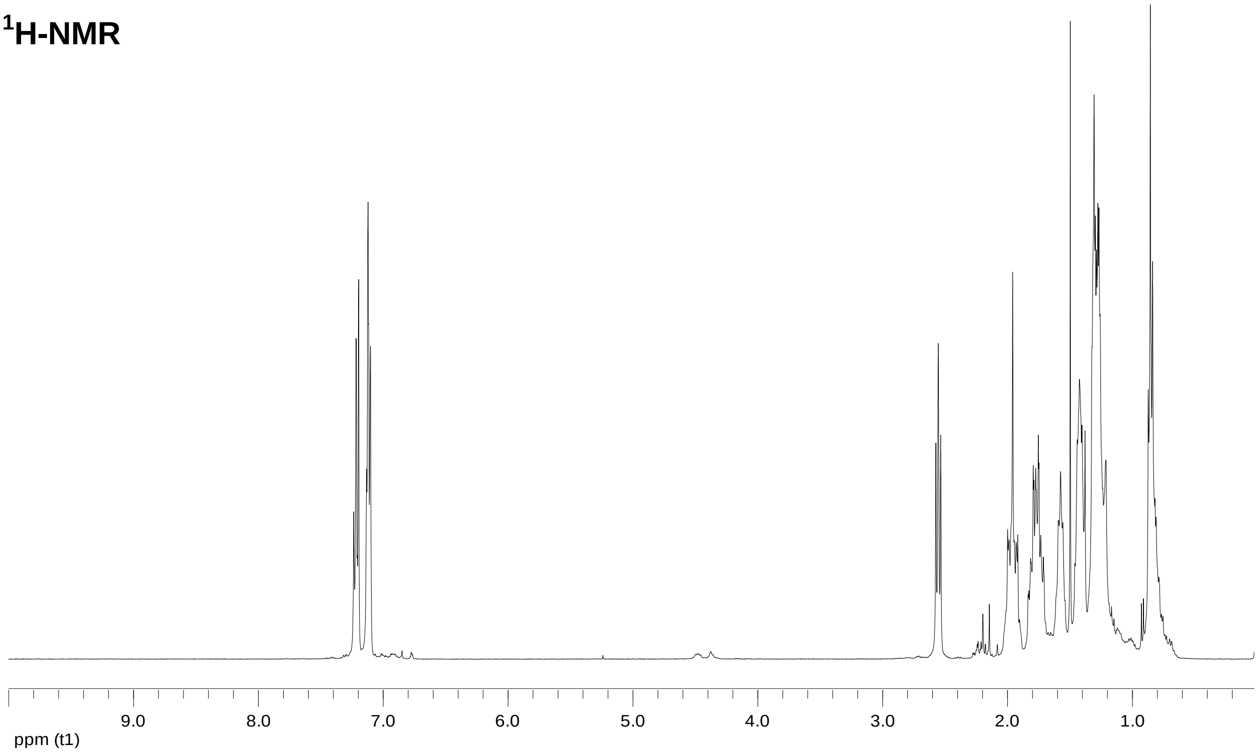

^{13}C-NMR

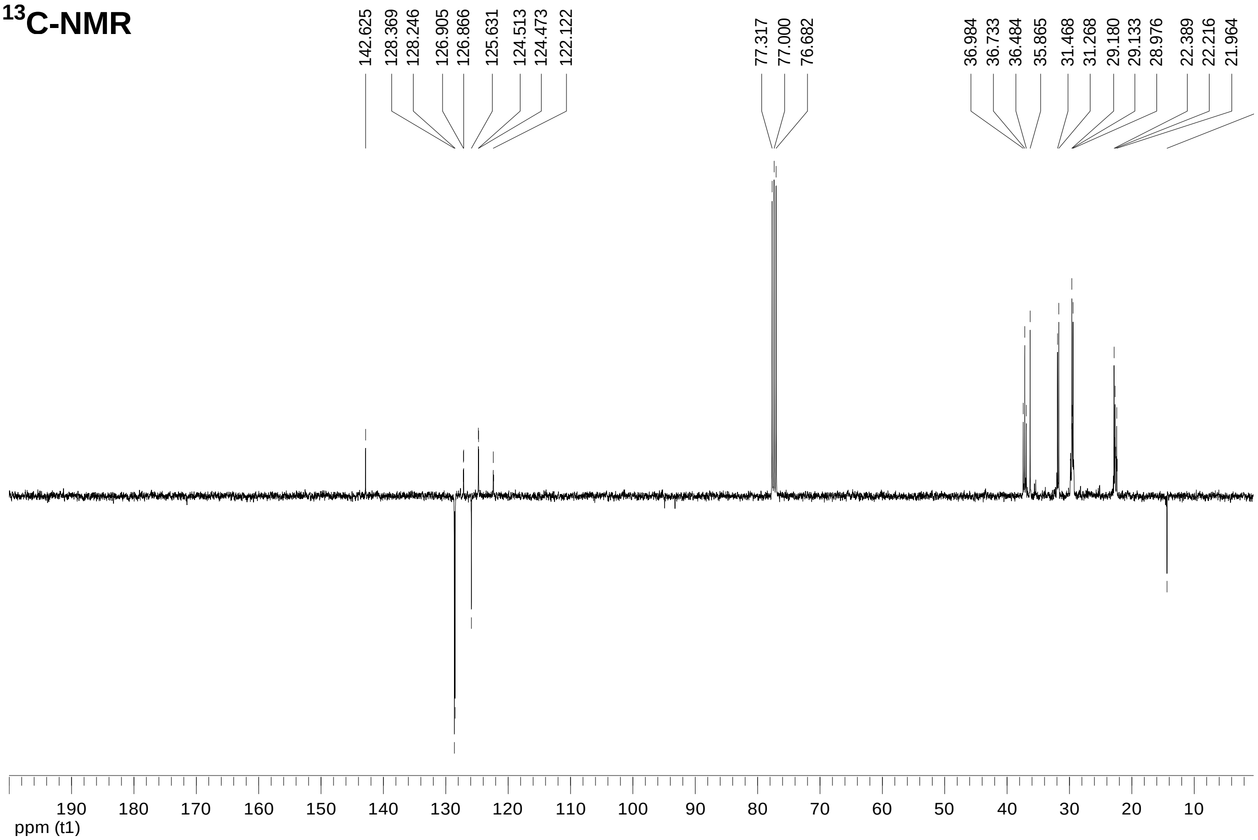

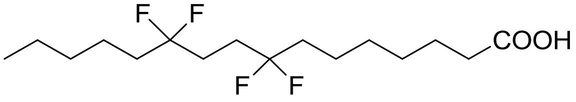

8,8,11,11-tetrafluorohexadecanoic acid (6c).

^{1}H-NMR

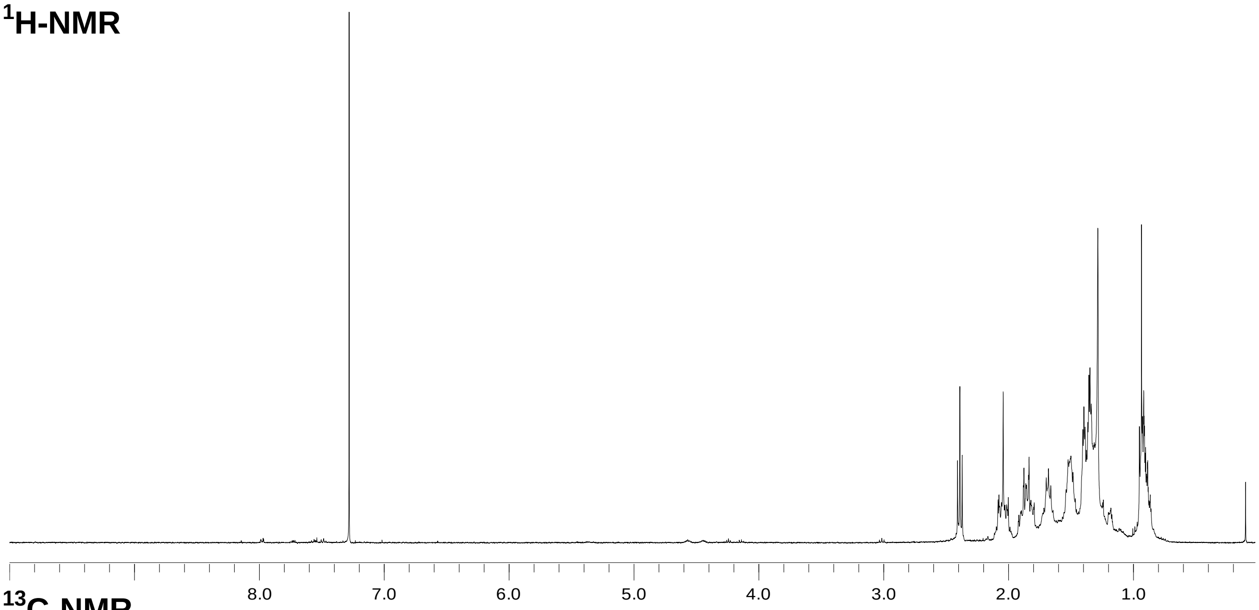

^{13}C-NMR

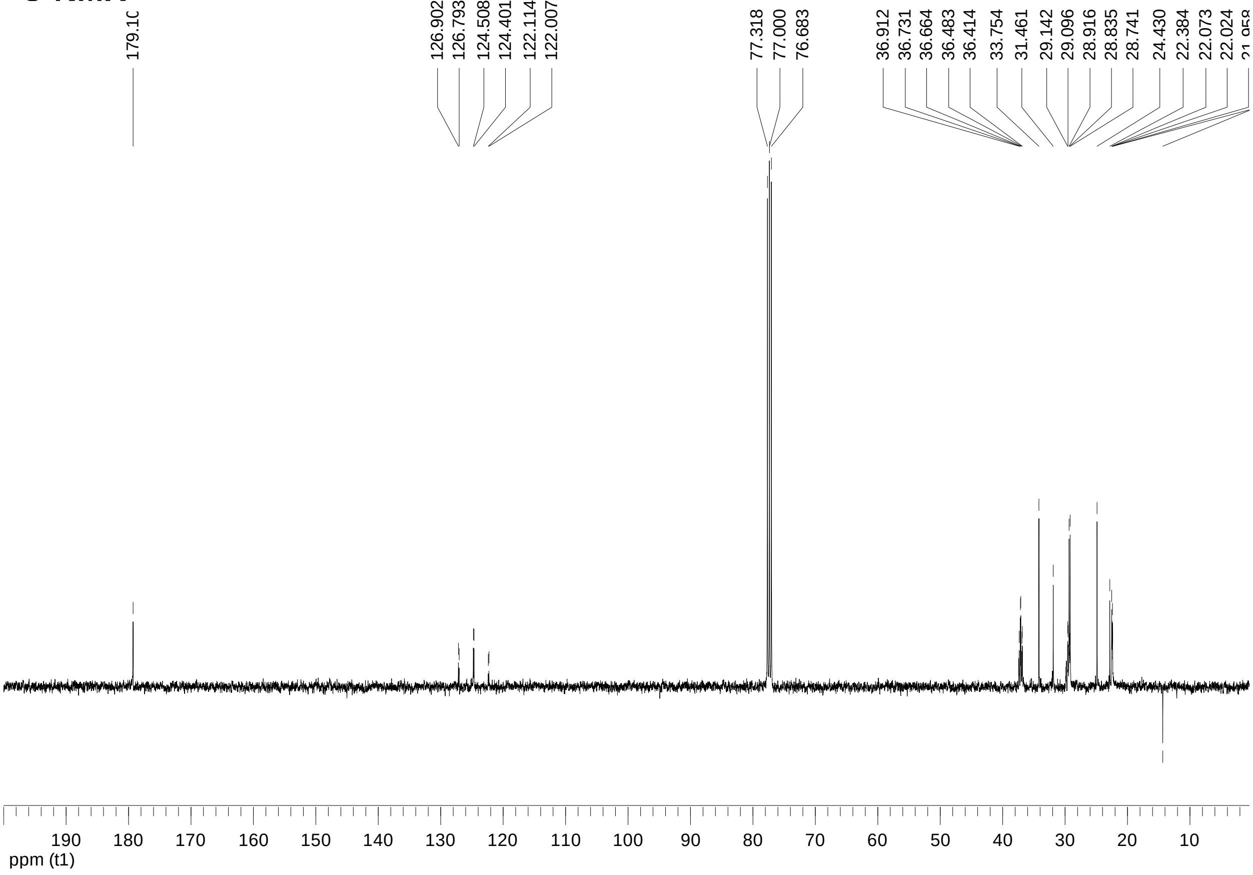

^{19}F-NMR

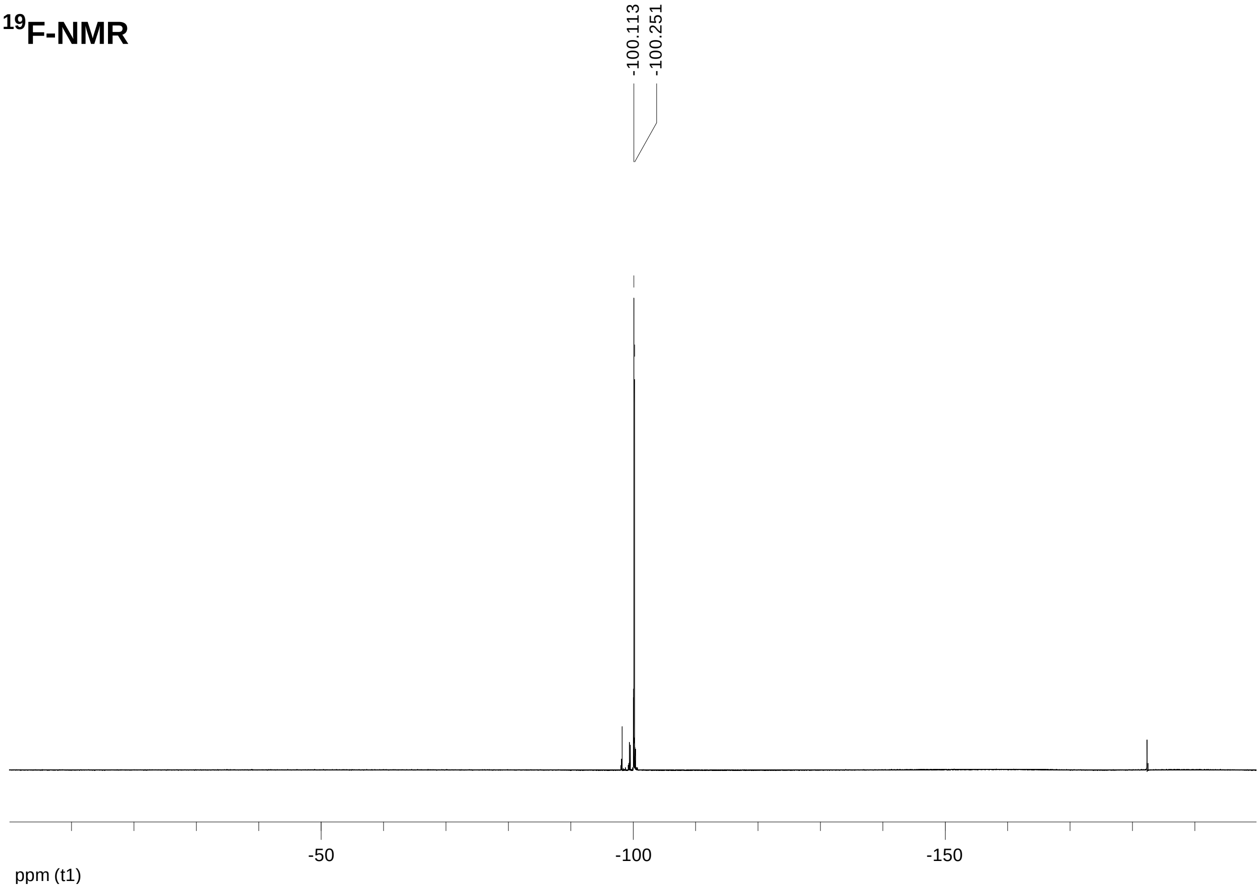

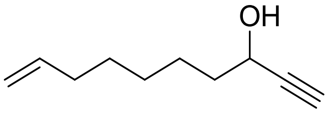

9-Decen-1-yn-3-ol (29).

^{1}H-NMR

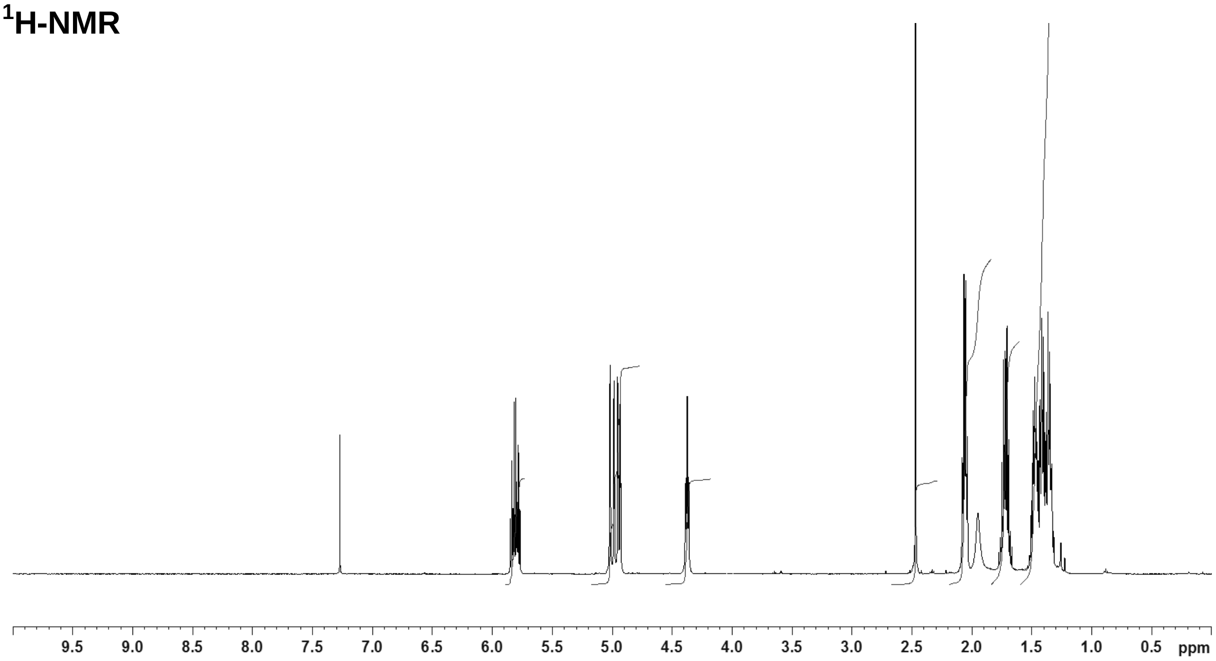

^{13}C-NMR

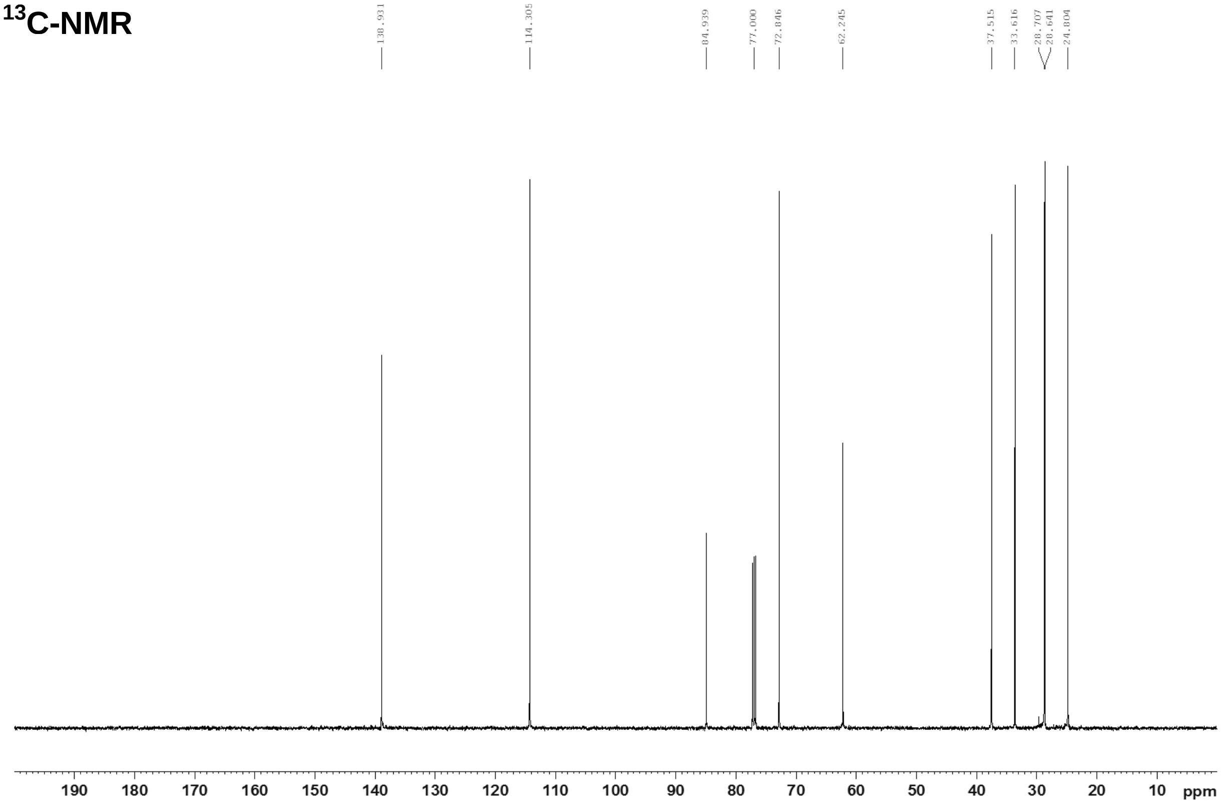

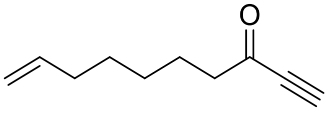

9-Decen-1-yn-3-one (30).

^{1}H-NMR

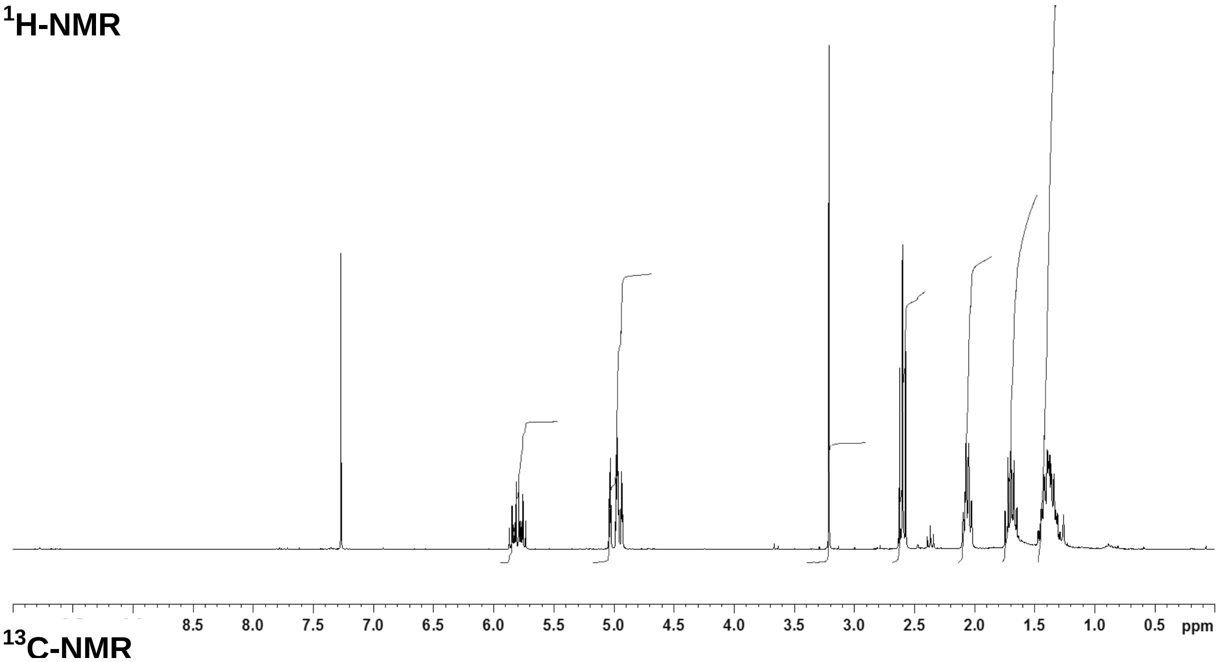

^{13}C-NMR

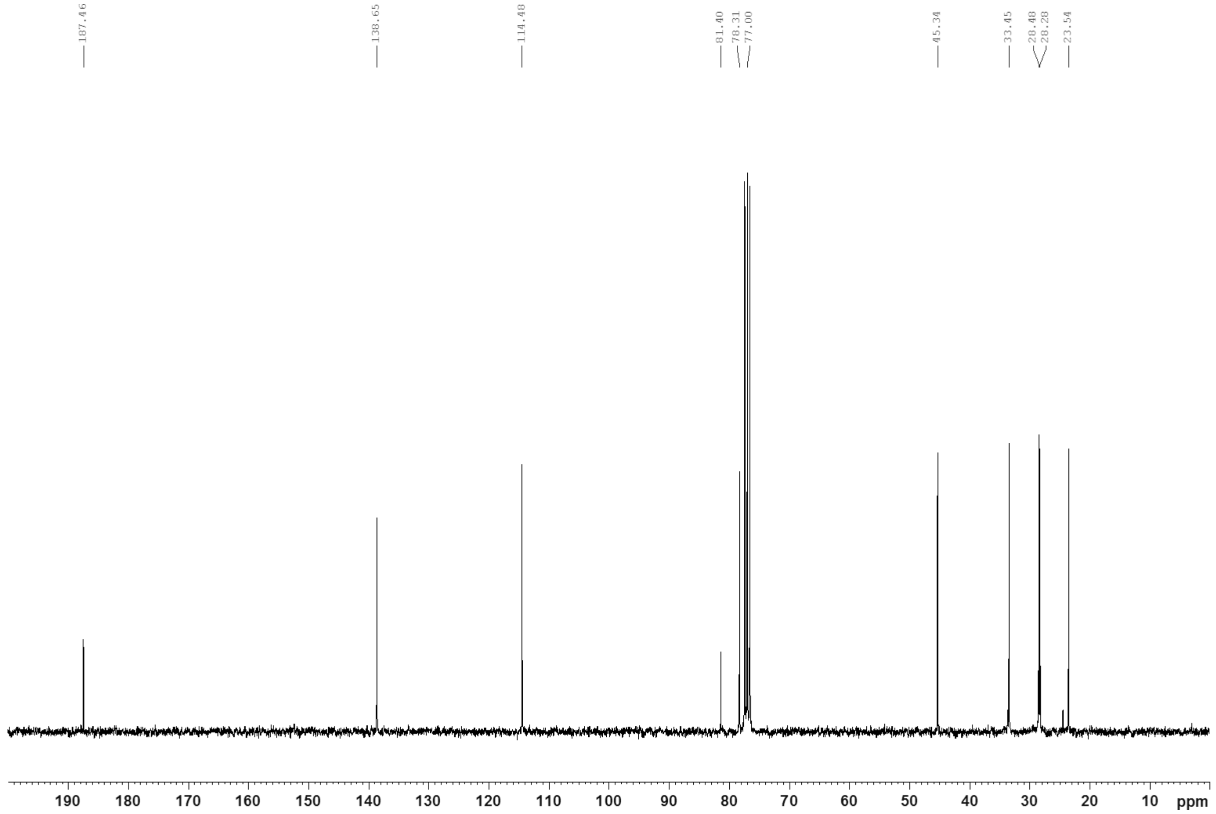

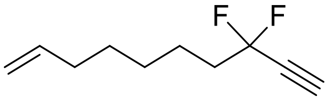

8,8-Difluoro-1-decen-9-yne (31).

^{1}H-NMR

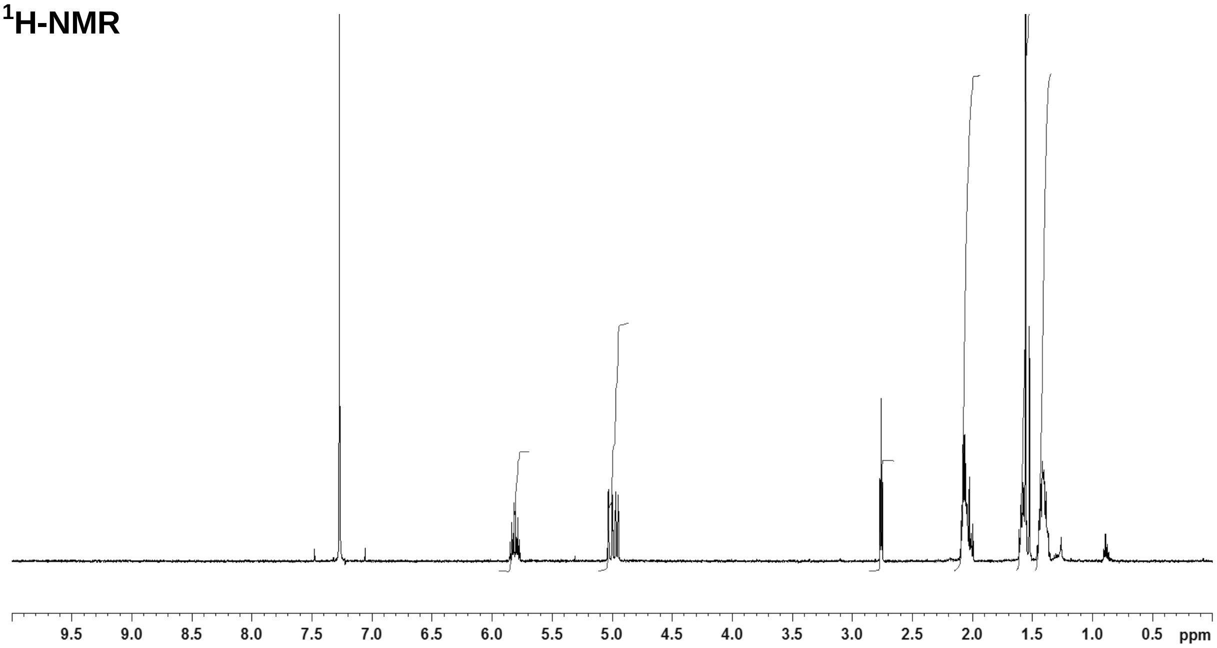

^{13}C-NMR

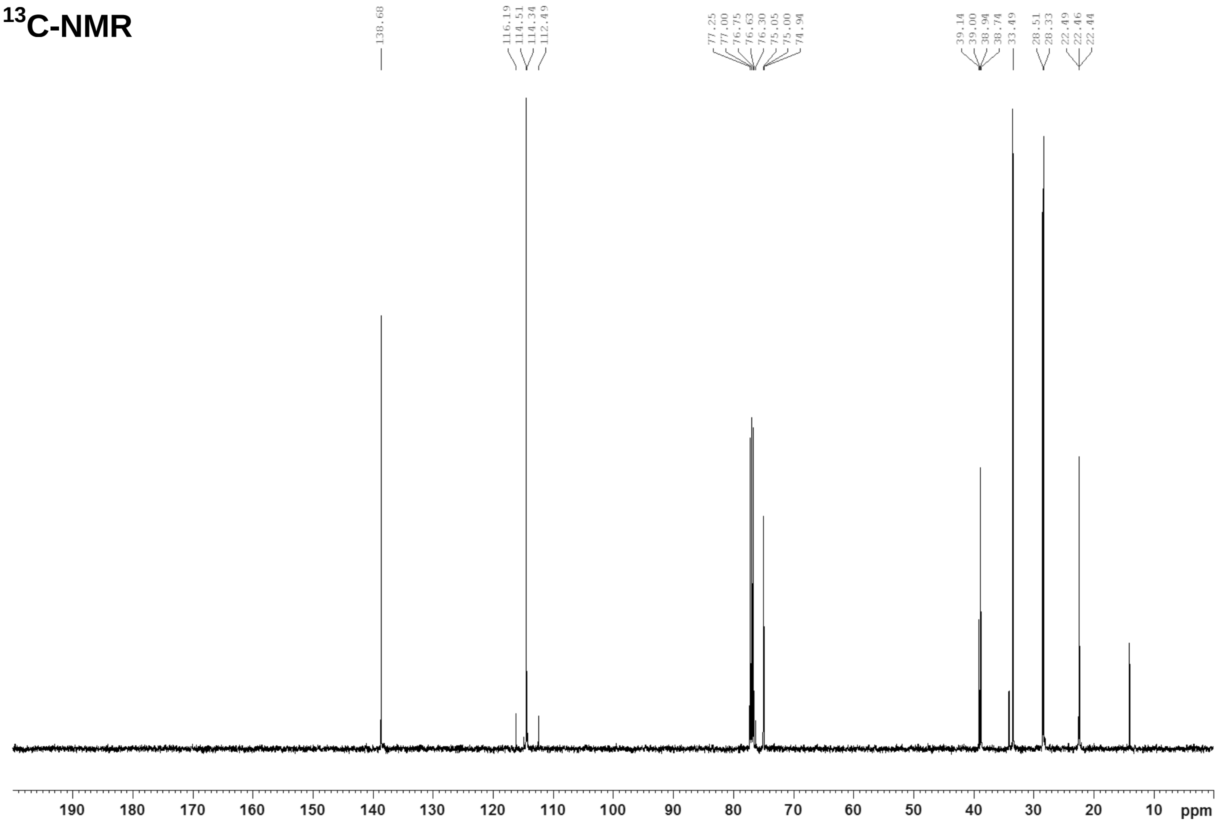

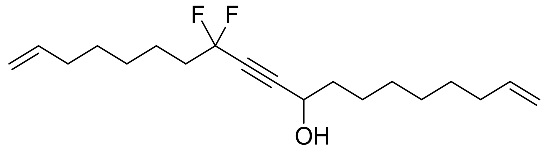

12,12-Difluorononadeca-1,18-dien-10-yn-9-ol (33).

^{1}H-NMR

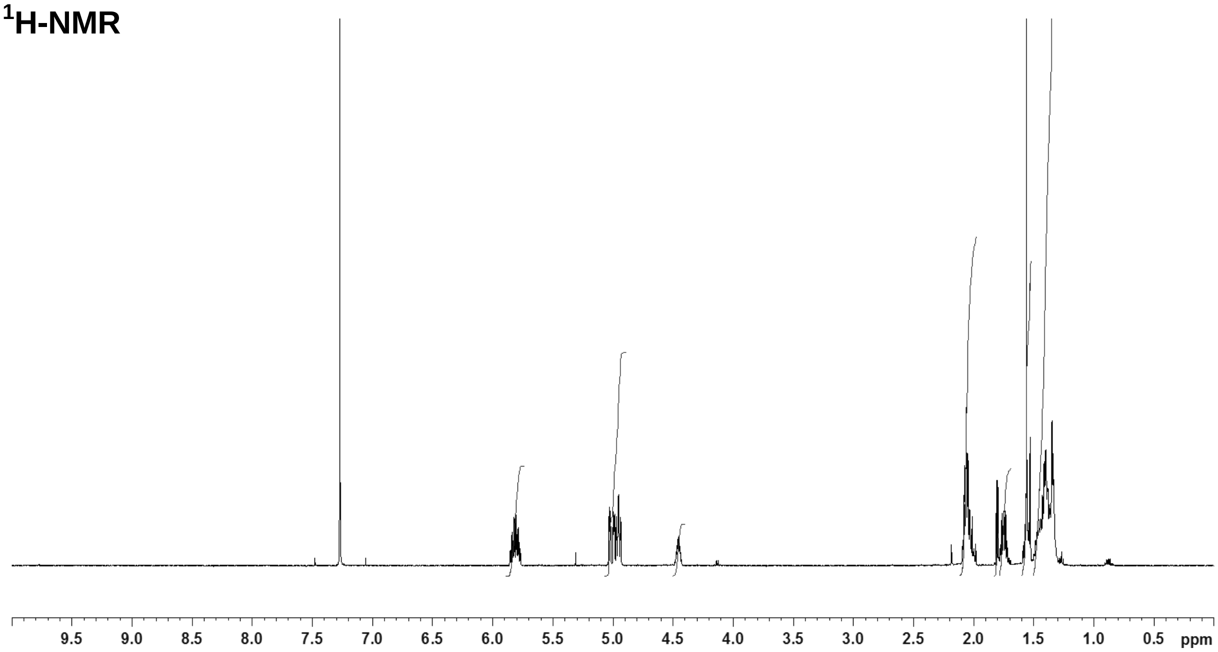

^{13}C-NMR

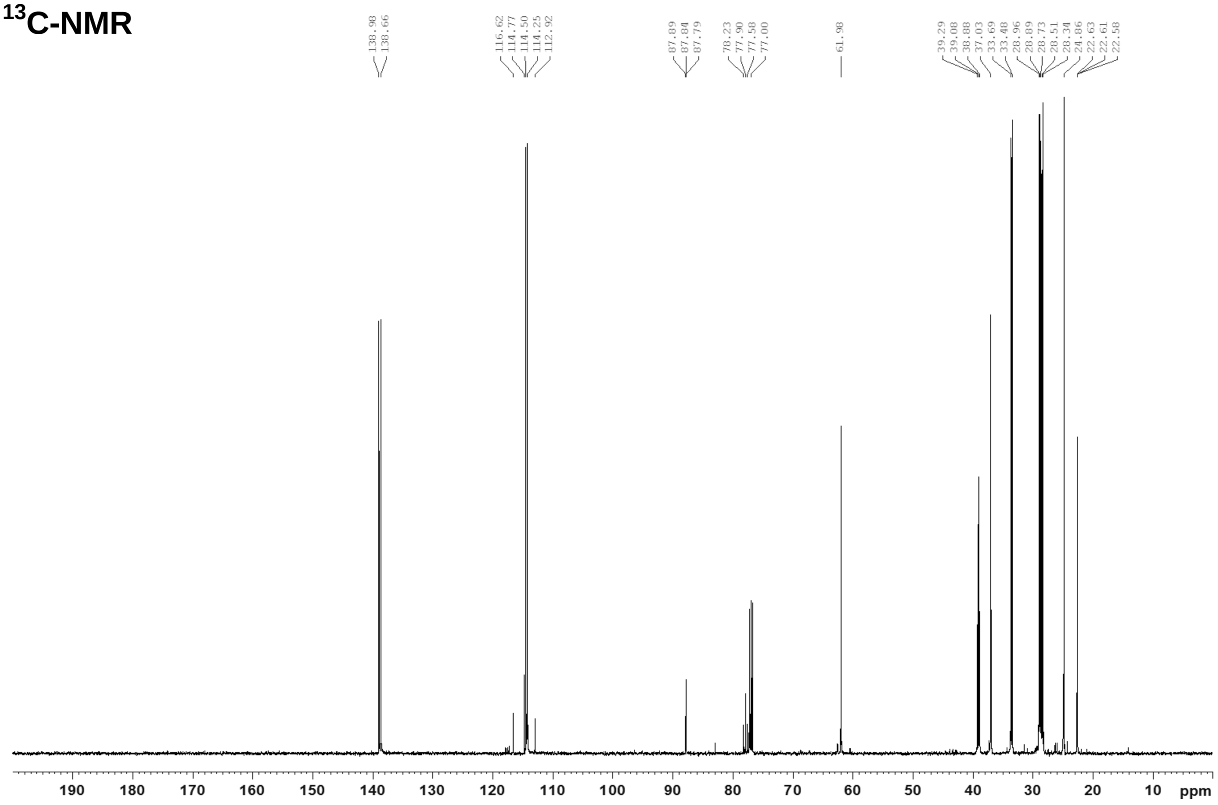

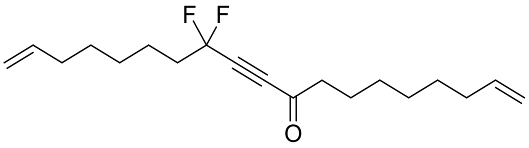

12,12-Difluorononadeca-1,18-dien-10-yn-9-one (34).

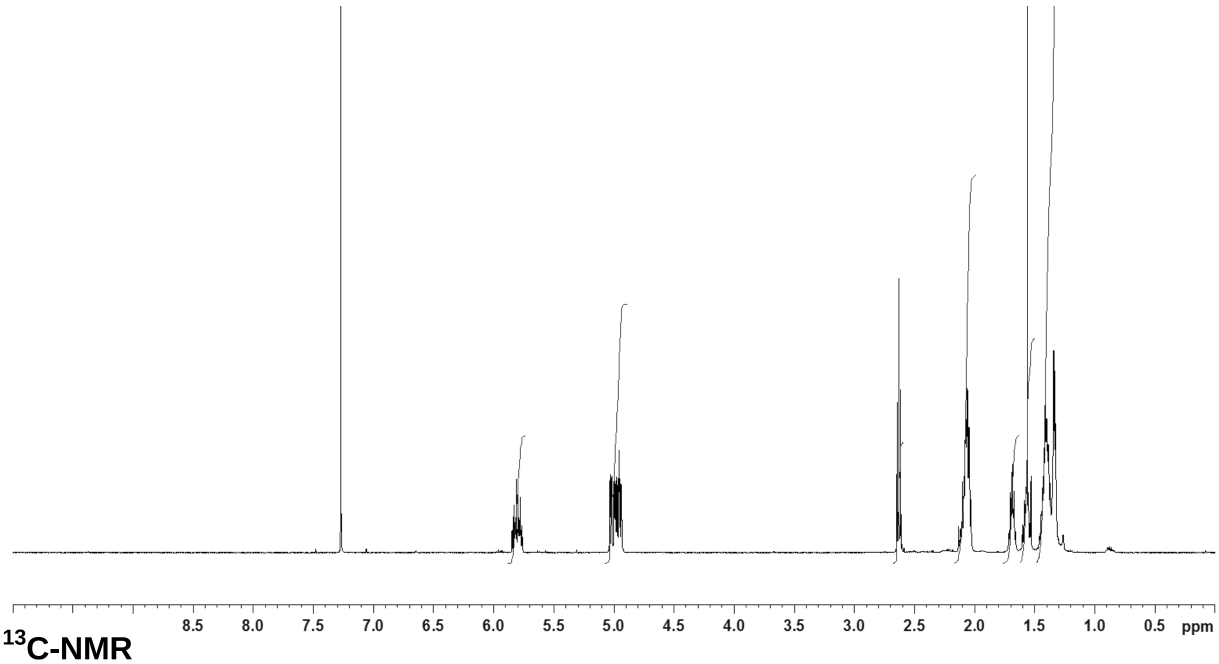

^{13}C-NMR

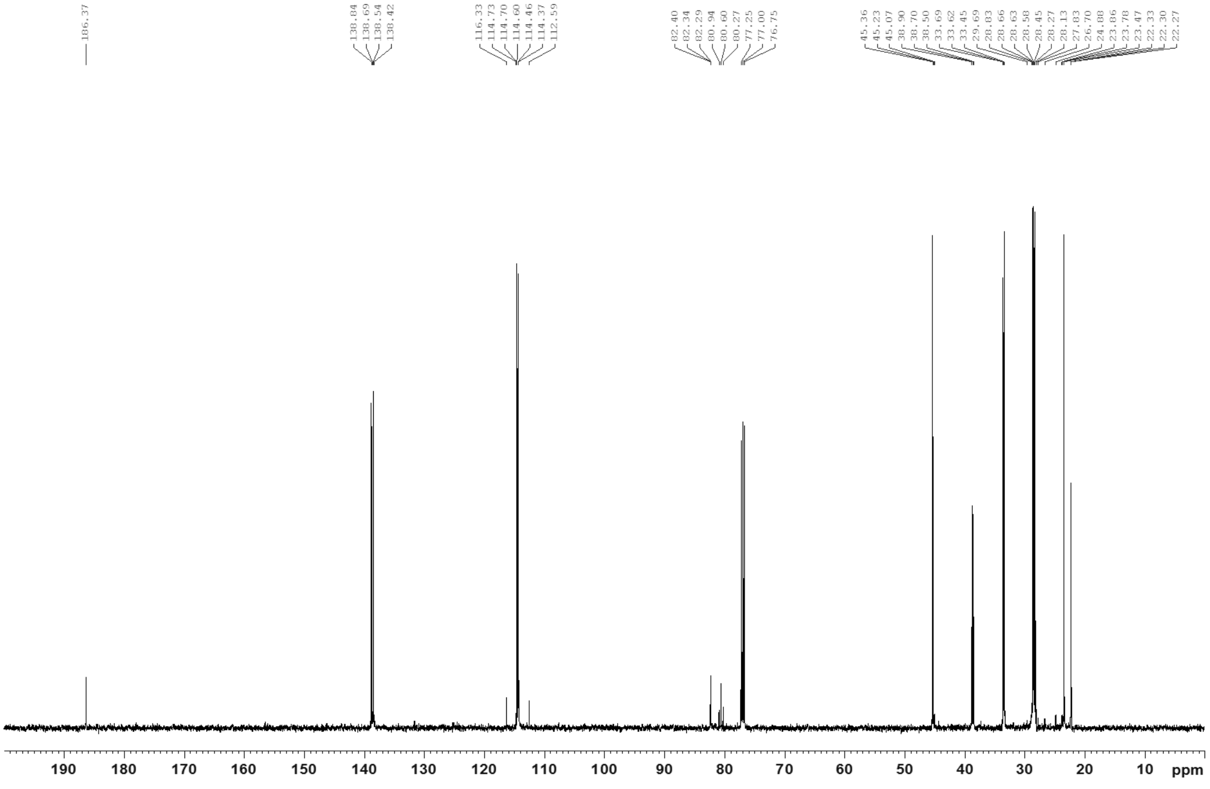

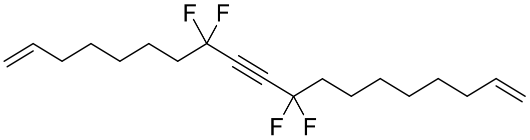

8,8,11,11-Tetrafluorononadeca-1,18-dien-9-yne (35).

^{1}H-NMR

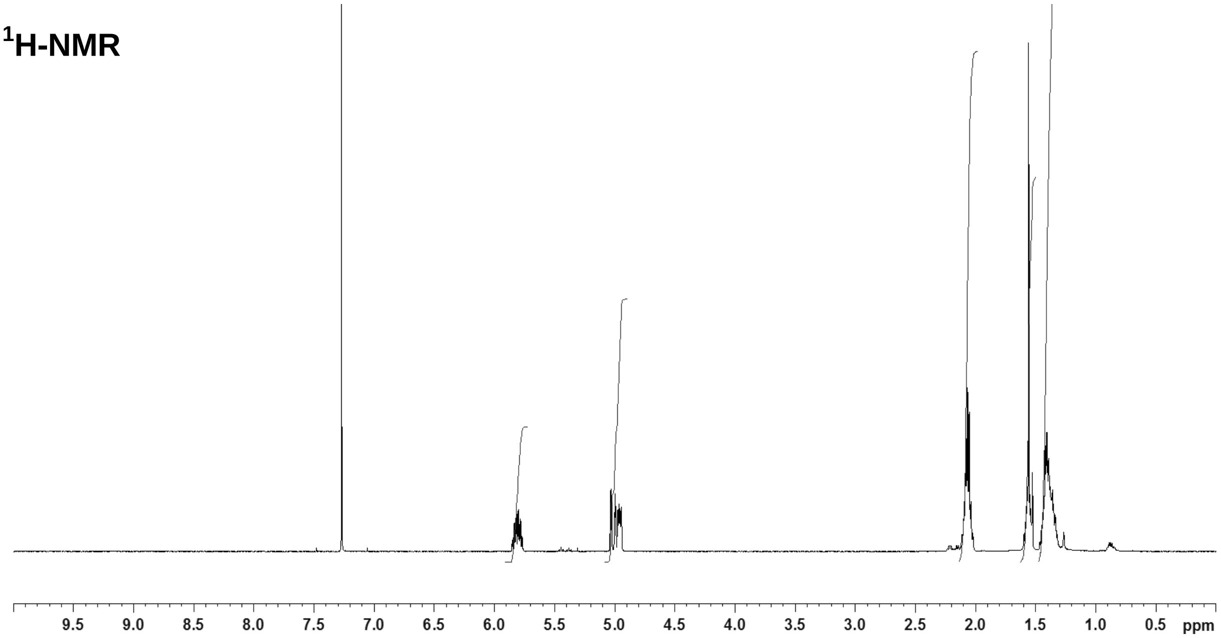

^{13}C-NMR

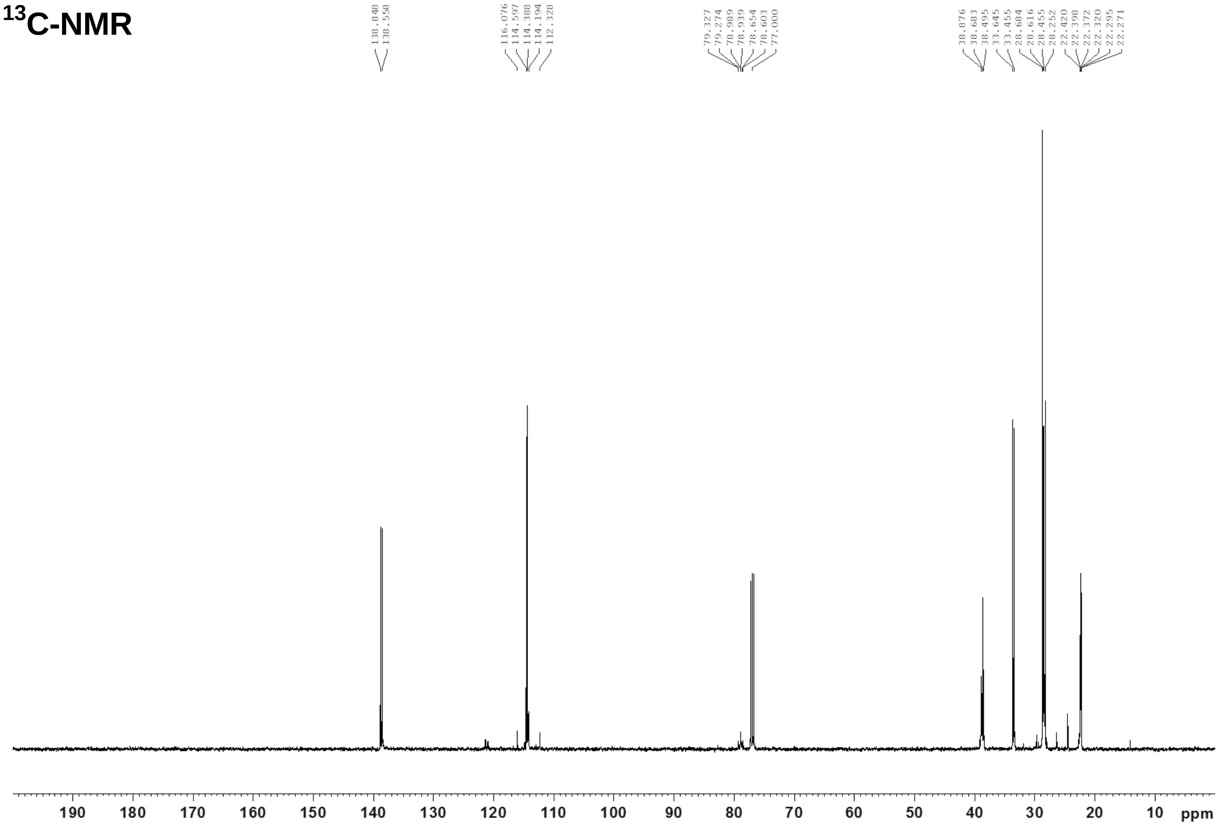

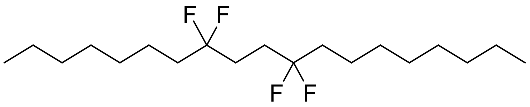

8,8,11,11-Tetrafluorononadecane (27).

^{1}H-NMR

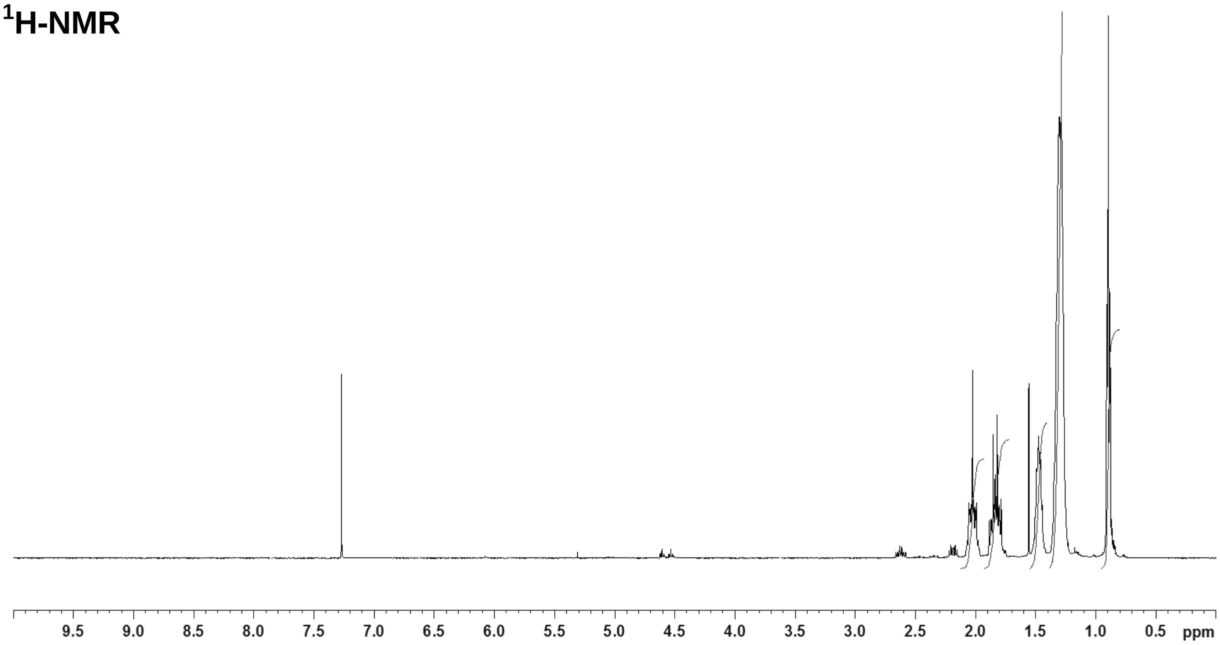

^{13}C-NMR

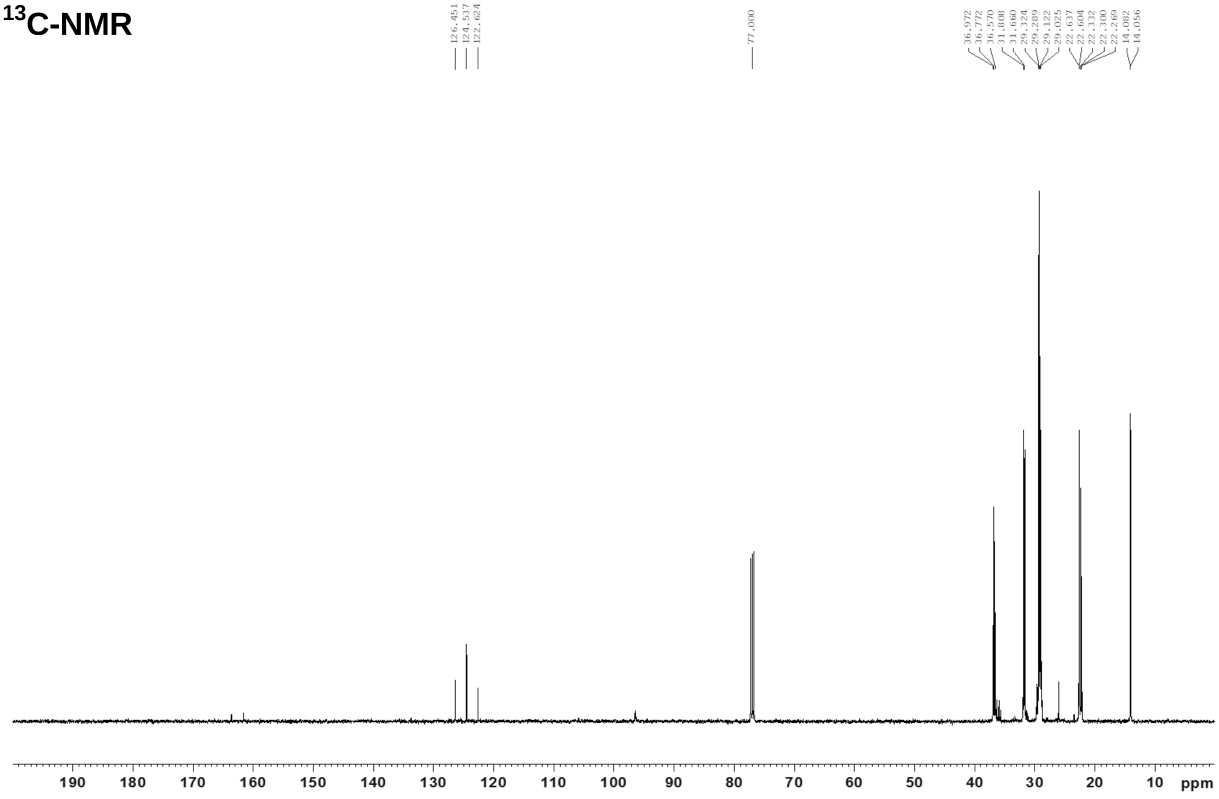

^{19}F-NMR

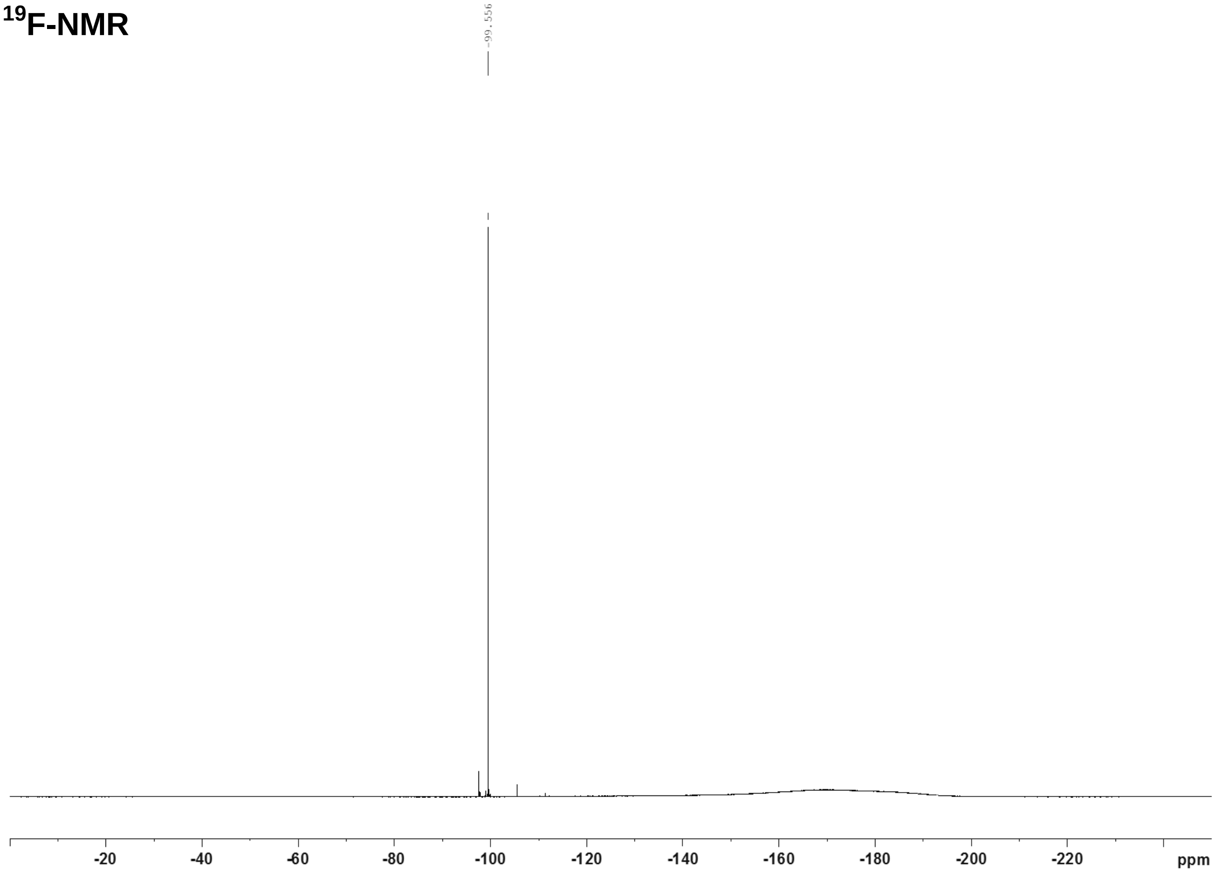

Supplement: File 1 — Experimental part. [file Beilstein_J_Org_Chem-10-18-s001.pdf]
